# The superior frontal longitudinal tract: a connection between the dorsal premotor and the dorsolateral prefrontal cortices

**DOI:** 10.1038/s41598-020-73001-7

**Published:** 2020-09-28

**Authors:** Mudathir Bakhit, Masazumi Fujii, Ryo Hiruta, Masayuki Yamada, Kenichiro Iwami, Taku Sato, Kiyoshi Saito

**Affiliations:** 1 Department of Neurosurgery, Fukushima Medical University, Fukushima-shi, Fukushima Japan; 2 Department of Neurosurgery, Aichi Medical University, Nagakute-shi, Aichi Japan

**Keywords:** Anatomy, Nervous system, Brain

## Abstract

A few studies have identified the structural connection between the premotor area and the lateral prefrontal cortex (DLPFC) as the frontal longitudinal system (FLS). This study investigated the existence of a direct segment (none U-fibre) of the superior part of the FLS (sFLS), which connects the dorsal premotor cortex (PMd) and DLPFC and analysed its asymmetry and termination point patterns. A dataset of diffusion-weighted images from 48 subjects was used for generalised q-sampling imaging tractography. Additionally, a white-fibre dissection was conducted in two right hemispheres. An analysis of spatial location, termination points, laterality, and correlation with the subjects’ gender or handedness was performed. The sFLS was found to have a deeper longitudinal bundle directly connecting the PMd and DLPFC. The bundle is referred to hereafter as the superior frontal longitudinal tract (SFLT). The SFLT was reconstructed in 100% of right and 88% of left hemispheres. It exhibited variable patterns in different subjects in their posterior terminations. In addition, it was found to possess a complicated spatial relationship with the adjacent bundles. The SFLT was revealed successfully in two cadaveric right hemispheres, where the posterior terminations were found to originate in the PMd independent of the superior longitudinal fasciculus.

## Introduction

The brain’s frontal lobe structural connectivity, namely the connection between the dorsal premotor area (PMd) and the dorsolateral prefrontal cortex  (DLPFC) is not well understood due to lack of sufficient data unfolding its details in humans. Several animal tracing, functional MRI, and probabilistic tractography analysis reports support the assumption of its existence^1–4^. An early study of primate brain tracing concluded that several premotor areas are interconnected with the prefrontal cortex, and the major site for interactions is the ventral part of the premotor area. In addition, the prefrontal cortex is interconnected with only a portion of the arm representation in three premotor areas (supplementary motor, cingulate motor and PMd)^1^. In the macaque monkey, Luppino et al. found that tracer injections in the PMd produced labelling in the prefrontal and agranular cingulate cortices in all cases. The dorsorostral part of the rostral PMd was a target of inputs from the frontal eye field, the dorsolateral prefrontal regions located dorsally (DLPFd) and ventrally to the principal sulcus, and from cingulate areas. In contrast, the remaining part of the rostral PMd is only a target of afferents from the DLPFd. Also, the ventro-rostral part of the caudal PMd receives inputs from the DLPFd and cingulate gyrus^2^.

Using probabilistic tractography in humans, Tomassini et al. identified a dorsal and ventral subregion of the premotor cortex (PMd and PMv). They revealed higher probabilities of connection for the PMd compared with the ventral subregion in both hemispheres for the cingulate gyrus, cingulate sulcus, and superior parietal lobule. For the left hemisphere, higher probabilities were only observed for the DLPFC and interparietal sulcus^3^. Schulz et al. studied the structural connectivity of prefrontal-premotor and prefrontal-motor connections using probabilistic tractography in a group of healthy aged participants and well-recovered chronic stroke patients. They were able to reconstruct probable trajectories connecting the DLPFC with the premotor (dorsal and ventral) and supplementary motor area (SMA) in both groups. Also, the DLPFC-premotor trajectories were restricted only to premotor regions with higher connectivity for SMA than for PMd; however, they could not reconstruct a direct prefrontal-motor connection between the DLPFC and primary motor cortex^4^.

The term ‘frontal longitudinal system’ (FLS) was introduced in a published study of the short frontal lobe’s connections^5^. Catani et al. conducted a spherical deconvolution diffusion tractography of 13 right-handed healthy adults’ brain images and postmortem dissections of one right hemisphere. The FLS was found as a chain of tracts of different length, with the majority showing a longitudinal course along a direction parallel to the superior and inferior frontal sulci. The superior chain of the FLS (sFLS) connects the precentral gyrus (PCG) to the ventral part of the superior frontal gyrus (SFG) and dorsal part of the middle frontal gyrus (MFG). The inferior chain (iFLS) projects from the PCG to the ventral part of the MFG and superior part of the inferior frontal gyrus (IFG). The same authors claimed that the FLS represents an extension of the superior longitudinal fasciculus (SLF) connecting fronto-parietal regions.

Recently, a comprehensive fibre dissection study of 15 hemispheres examined the morphology, connectivity and correlative anatomy of the FLS^6^. The FLS was encountered just under the most superficial U-fibres at the depth of the middle frontal sulcus in 80% of hemispheres. The deeper layer was composed of longer fibres connecting more remote areas in the premotor and prefrontal cortices running in two separate levels, superior and inferior, resembling the superior and inferior longitudinal chain of fibres described by Catani et al. Posteriorly, the fibres of the FLS were always observed to terminate in the PMd and PMv. Anteriorly, the DLPFC received fibres in all the studied hemispheres. In most hemispheres, the FLS was macroscopically identified as an anterior extension of the SLF fibre system, while in 20% of the studied hemispheres, the SLF and FLS were recorded as two completely distinct tracts.

Here, we attempt to expand on the works introduced in the aforementioned reports by conducting a comprehensive virtual dissection of the sFLS in a large sample of healthy subjects. Using generalised q-sampling imaging (GQI) tractography, we examine its shape, termination patterns and their incidence, and spatial relationship with surrounding structures. GQI is a non-tensor tractographic modality permitting a high angular resolution-based approach that leverages high directional sampling of the diffusion imaging space to produce high-resolution imaging of white matter structures^7^. As it is a non-tensor modality, it permits visualisation of crossing fibres and accurate delineation of close-proximity fibre systems. Where diffusion tensor imaging (DTI) has failed, GQI has been shown to accurately replicate sophisticated, known neuroanatomical features, such as the crossing fibres of the optic chiasm, the decussating fibres of the corticospinal tracts (CST) in the cerebral peduncle, and the crossing point for the CST, the corpus callosum (CC), and the arcuate fasciculus (AF)^8^. Furthermore, GQI tractography has achieved the highest 92% valid connection examined by open competition in 2017; the average accuracy was 54%^9^.

After reviewing the published tractography data, we were more inclined towards the hypothesis that in addition to the superficial U-fibres system (sFLS) another deep longitudinal bundle does exist. The latter is referred to hereafter as the superior frontal longitudinal tract (SFLT), and the term ‘sFLS’ is reserved for the superficial U-fibre chain. We also hypothesised that the SFLT is a separate, frontal, intralobar tract and not a continuation of the SLF.

In addition to the GQI tractography, a cadaveric white matter dissection of two brain samples is conducted in an attempt to confirm the termination of the SFLT, and its anatomical relation to the SLF.

## Results

### GQI tractography

**Figure 1:**
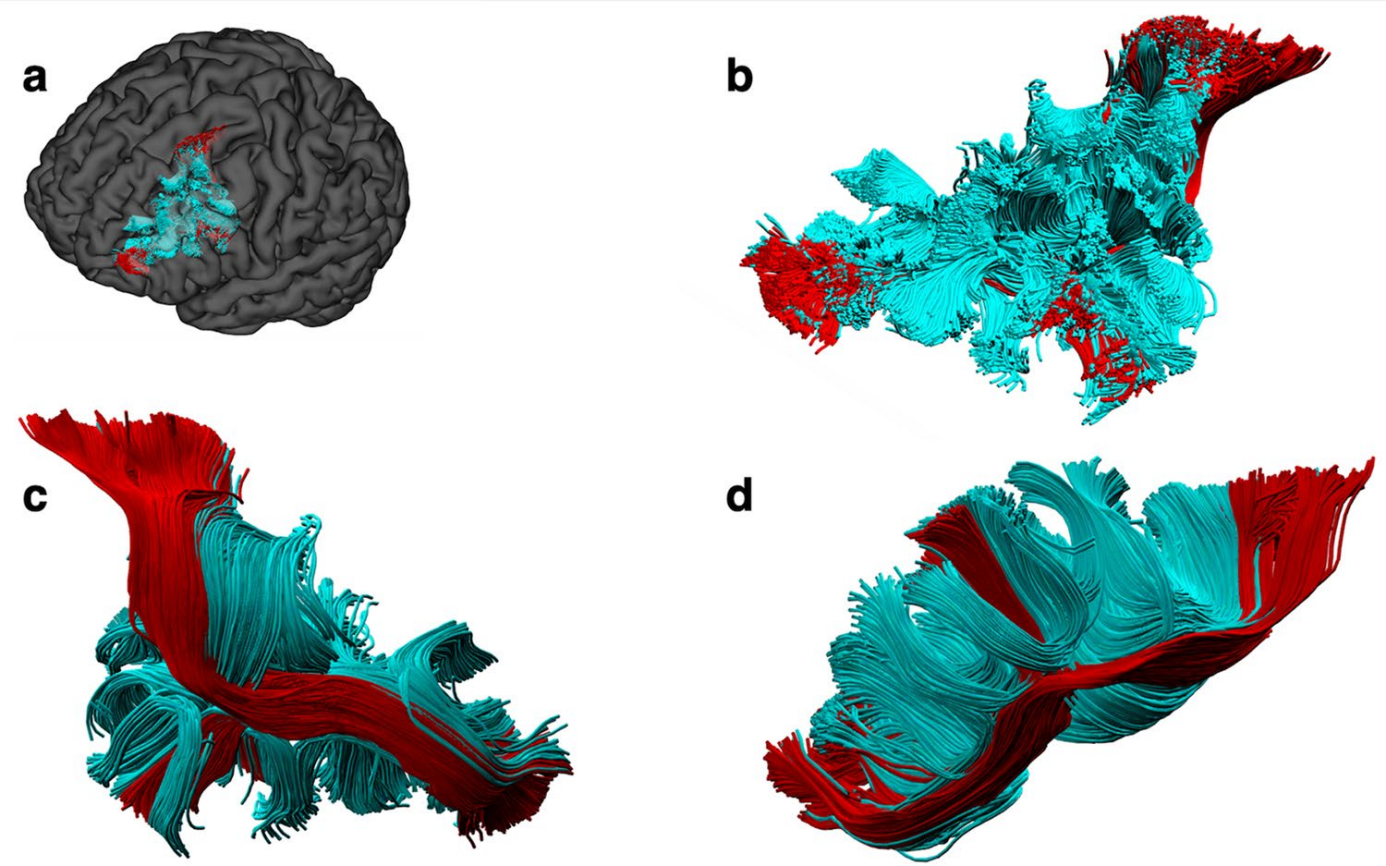
The spatial relation between the sFLS and SFLT in a single subject. The SFLT (red) directly connecting the PMd with the DLPFC lies ventromedial to a chain of U-fibres of the sFLS (cyan). (**a**) Tracts demonstrated in a left hemisphere mask. (**b**) Dorsolateral view. (**c**) Ventromedial view. (**d**) Lateral view.

GQI tractography was conducted using the imaging data of 48 healthy adults from the Human Connectome Project (HCP) database^10^. The SFLT was successfully constructed in all 48 (100%) of the right hemispheres and 42 (88%) of the left hemispheres (Supplementary Fig. S1). The SFLT can be distinguished from the superficial U-fibres of the sFLS (an example is shown in Fig. 1, and Supplementary Methods-Fig. S8). Several cases demonstrated two bundles (one running dorsally and the other ventrally), which were mostly the sFLS and iFLS, respectively^5,6^. As the inferior FLS is beyond the scope of this study, it was removed.

The average lengths (mean ± SD) of the left and right SFLT were 67.72 ± 8.65 mm and 63.70 ± 9.04 mm, respectively. The streamlines excluded all possible U-shape like streamlines and had a mean length above 50 mm. An exception was in one right hemisphere (subject # 9, mean length = 43.86 mm) that displayed a deep anatomical location with a rostro-caudal longitudinal stream (Supplementary Fig. S1).

### Spatial location

**Figure 2:**
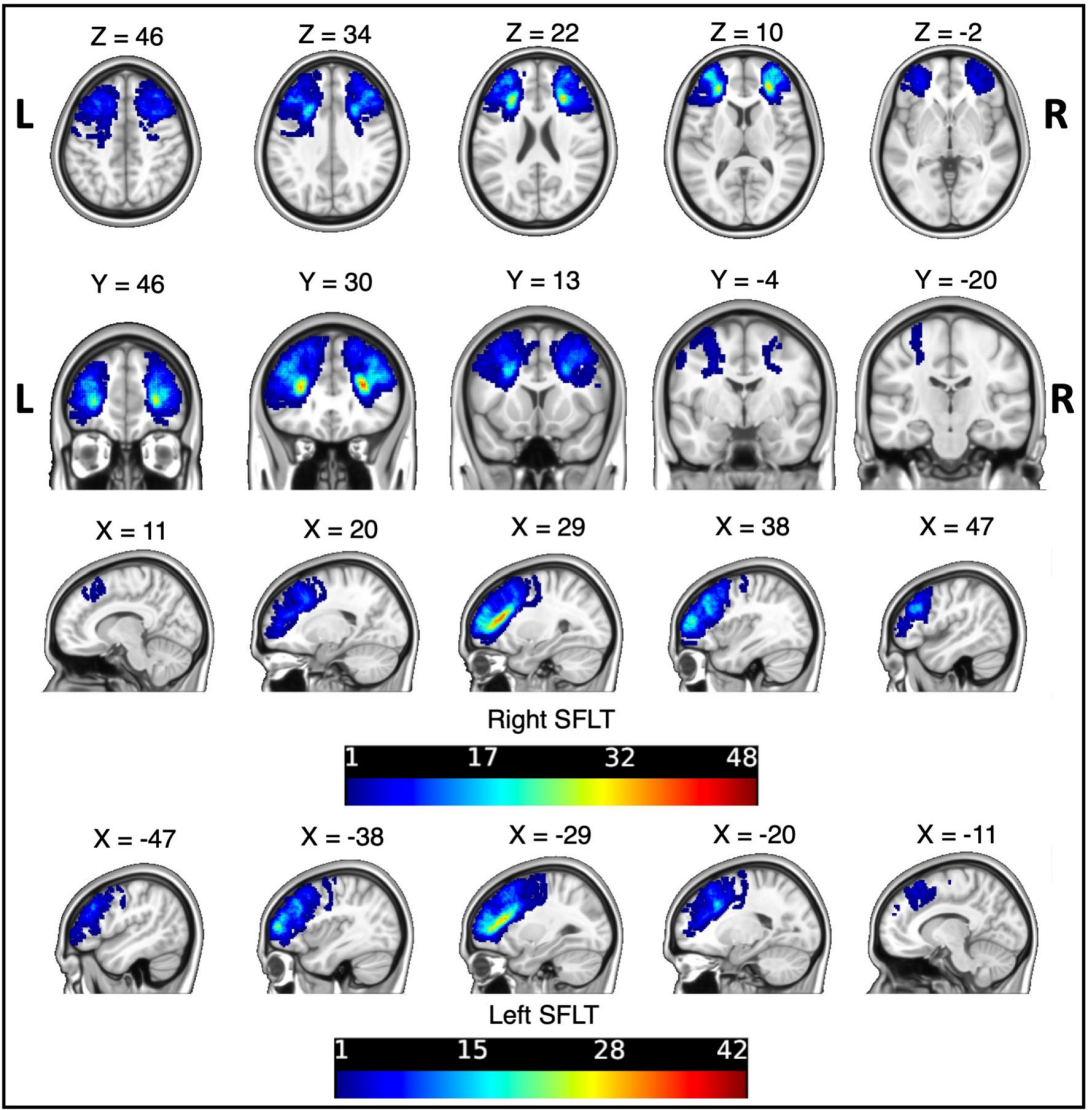
An overlap image of the SFLT’s streamline of 45 subjects and 48 subjects in the left and right hemispheres, respectively. Overlap image generated by MRIcron and displayed by Connectome Workbench software. *Colour map: JET256*.

Figure 2 shows the overlap image of the SFLT on both hemispheres in the 48 subjects. The SFLT running path of all subjects lies in the deep white matter substance of the MFG, and voxels with the higher frequency of SFLTs are located at the area antero-lateral to the anterior horn of the lateral ventricles.

### Subcomponents of the SFLT

**Figure 3:**
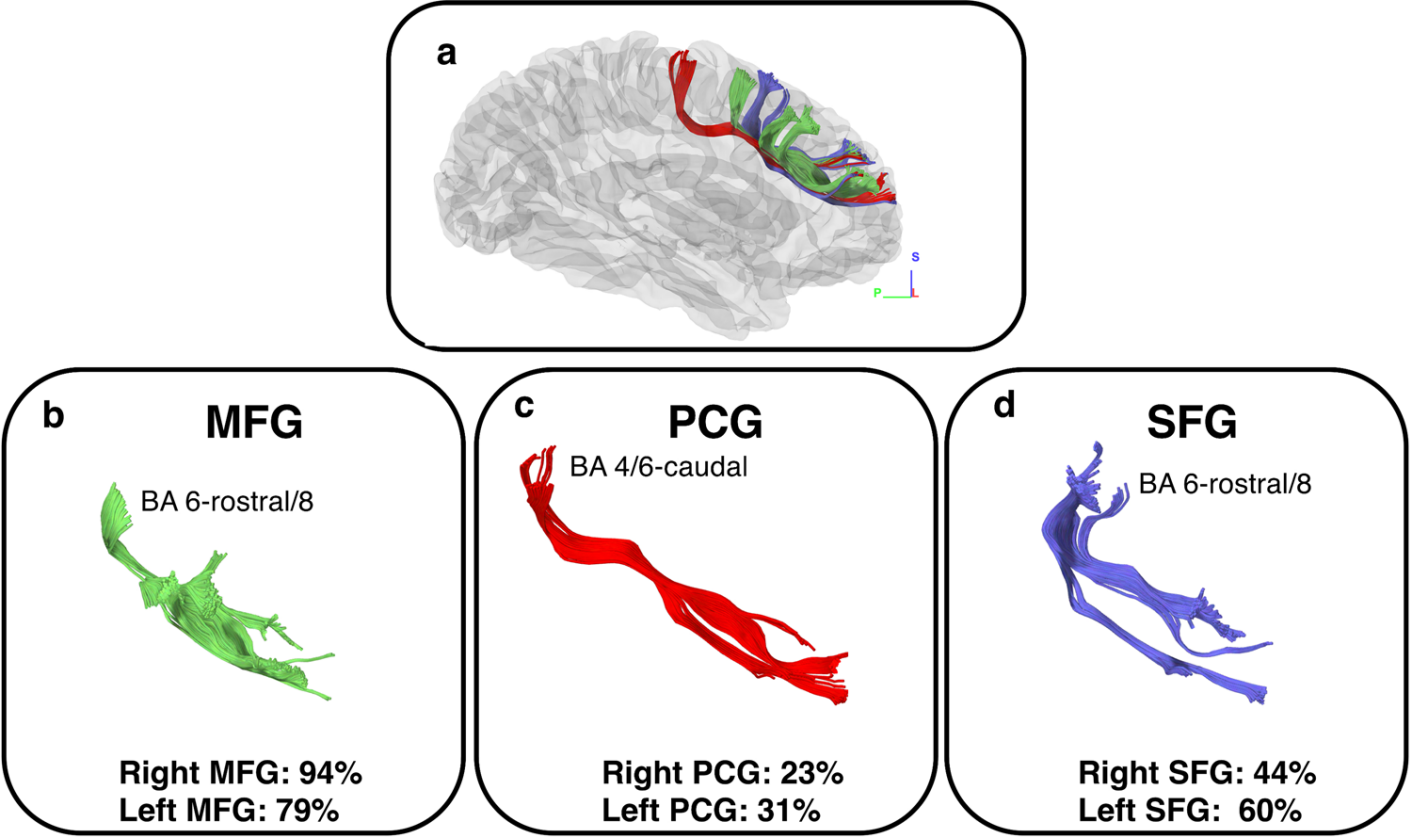
Constructed subcomponents of a right side SFLT of one subject based on caudal termination points. The subcomponents of SFLT together (**a**). The Brodmann areas (BAs) of the caudal termination points, and the occurrence rate of the MFG, PCG and SFG subcomponents are displayed (**b**–**d**, respectively).

**Figure 4:**
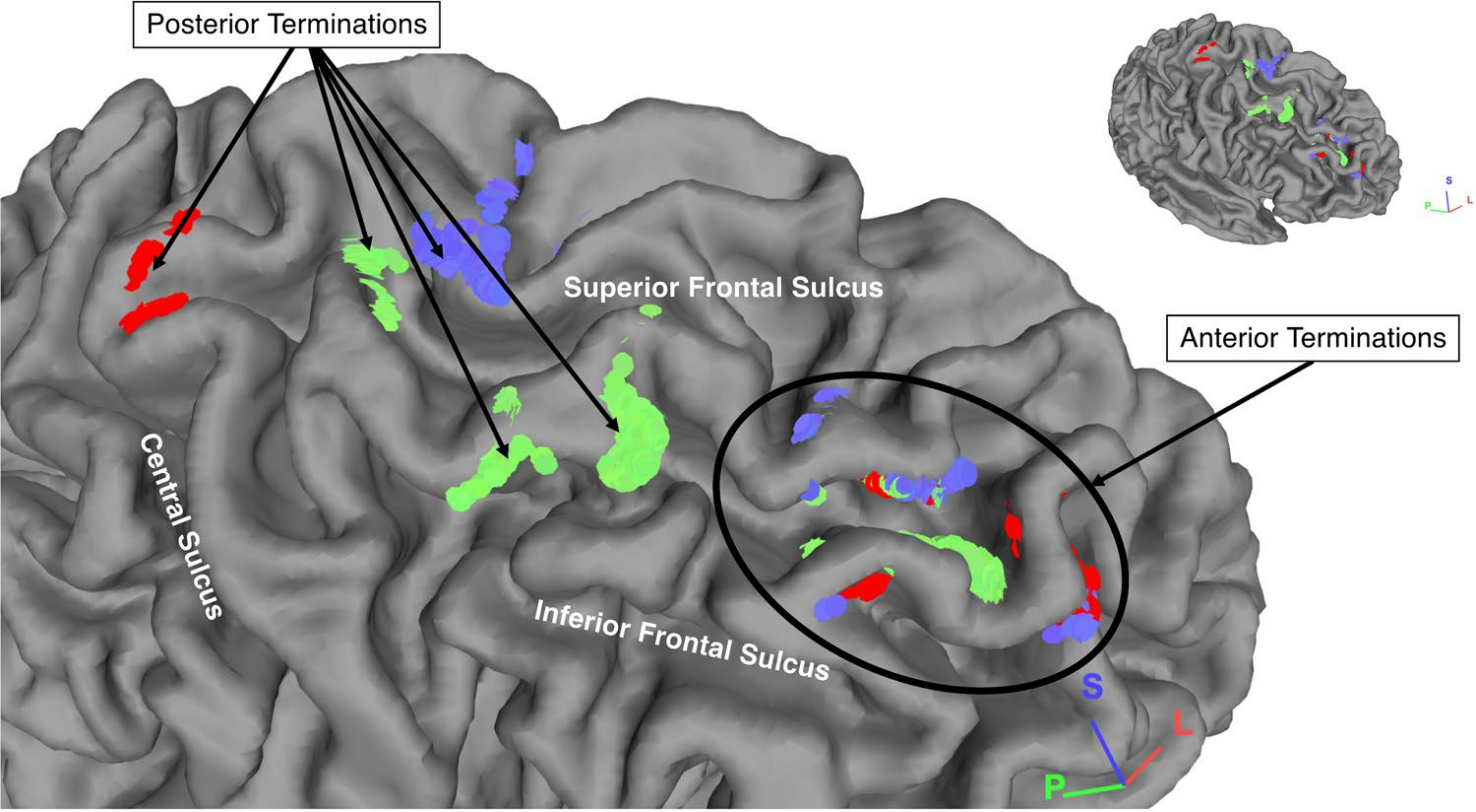
The terminations points of a right-side SFLT of one subject. The MFG, PCG and SFG subcomponents are in green, red and blue colours, respectively.

The SFLT subcomponent regions and probable Brodmann areas (BAs) of the terminations are shown in Fig. 3, and an example from one subject is shown in Fig. 4. The GQI tractography of the SFLT revealed variations in the posterior terminations. Conversely, the anterior termination was mostly the same in the anterior part of the rostral MFG (rMFG) and in the frontal pole (FP) (BA 9/46/10). The posterior terminations were in the MFG and SFG (BA 6-rostral/8) or the PCG (BA 4/6-caudal). The occurrence rate of these subcomponents varied, as demonstrated in Fig. 3. The MFG subcomponent was present in the left and right hemispheres in 38 (79%) and 45 (94%) subjects, respectively. The PCG subcomponent was present in the left and right hemispheres in 15 (31%) and 11 (23%) subjects, respectively. The SFG subcomponent was present in the left and right hemispheres in 29 (60%) and 21 (44%) subjects, respectively. Five subjects had an SFLT lacking the BA6 terminations in both hemispheres (Supplementary Fig. S1). Another 12 subjects showed SFLT with a unilateral BA6 termination.

### Spatial relations with adjacent white matter bundles

**Figure 5:**
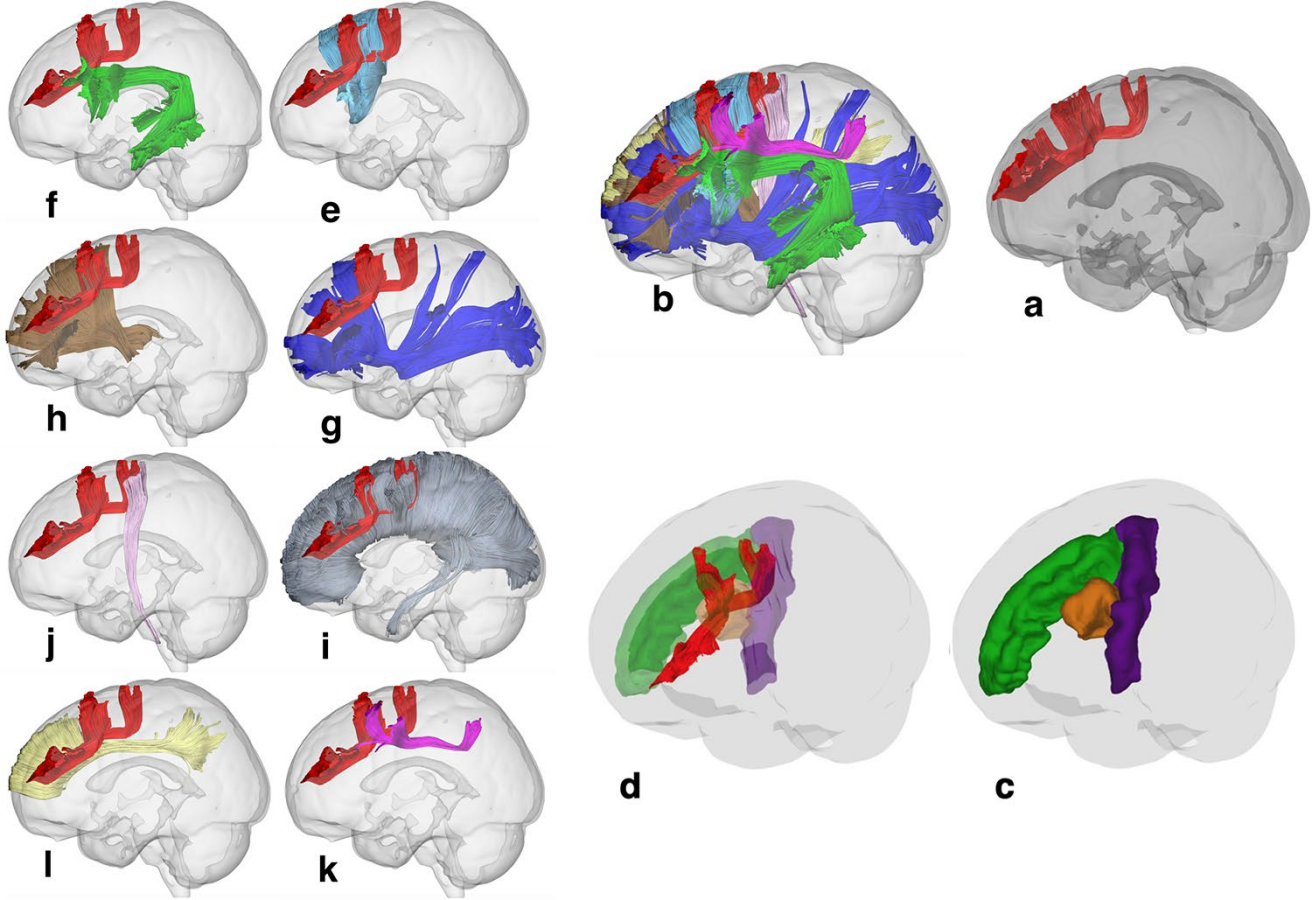
Spatial anatomical relationships of the left SFLT. (**a**) The constructed SFLT from the HCP-1021 average template. (**b**) The SFLT with all related white matter fibres (the commissural fibres are not included for the sake of clarity). (**c**,**d**) The left SFLT in relation to the PCG (violet), SFG (green), cMFG (orange) and rMFG (blue). (**e**) Frontal aslant tract. (**f**) Arcuate fasciculus. (**g**) Inferior fronto-occipital fasciculus. (**h**) Anterior projection fibres. (**i**) Corpus callosum. (**j**) Corticospinal tract. (**k**) Superior longitudinal fasciculus. (**l**) Cingulate bundle.

**Figure 6:**
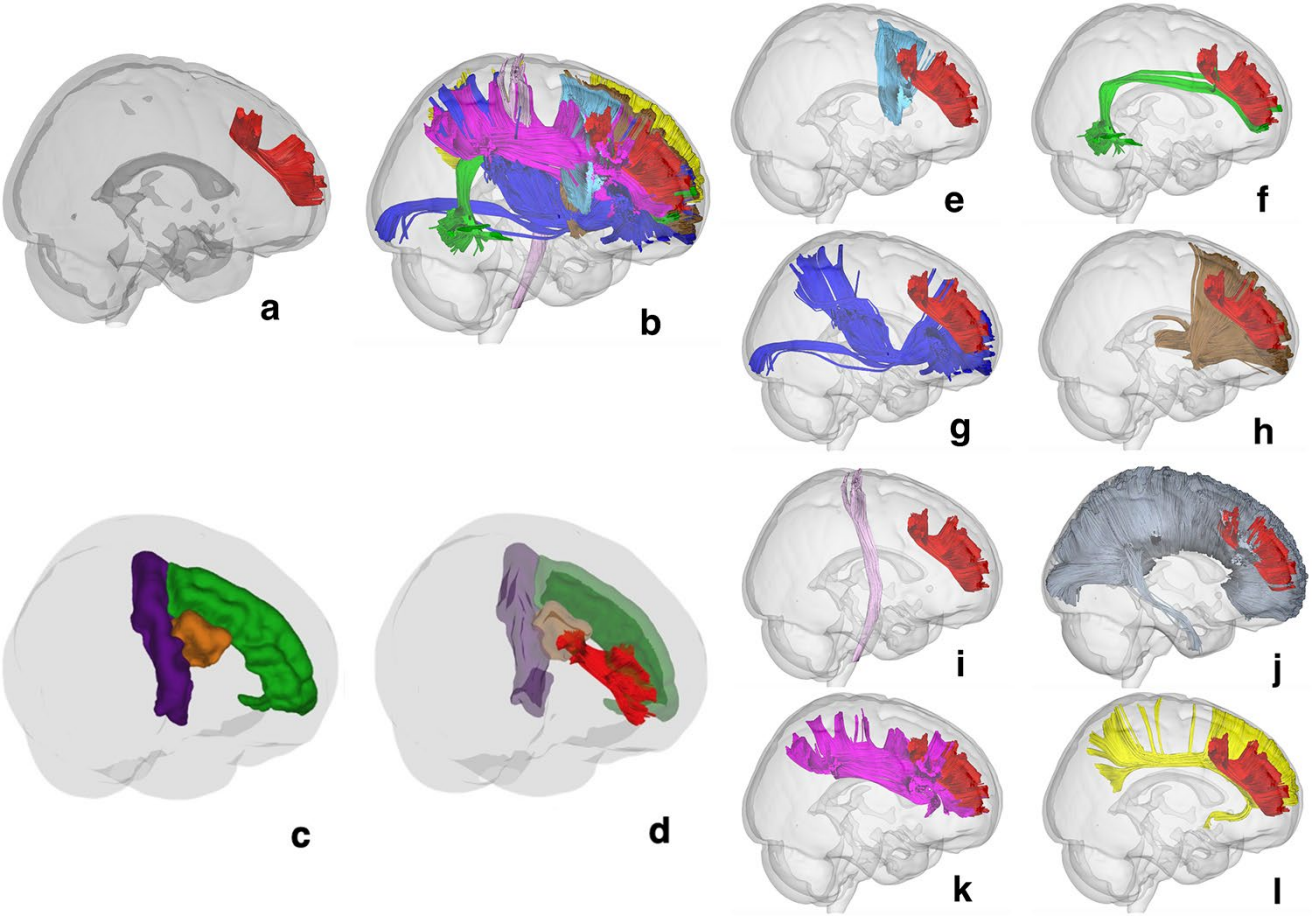
Spatial anatomical relationships of the right SFLT. (**a**) The constructed SFLT from the HCP-1021 template. (**b**) The SFLT with all related white matter fibres (the commissural fibres are not included for the sake of clarity). (**c**,**d**) The left SFLT in relation to the PCG (violet), SFG (green), cMFG (orange) and rMFG (blue). (**e**) Frontal aslant tract. (**f**) Arcuate fasciculus. (**g**) Inferior fronto-occipital fasciculus. (**h**) Anterior projection fibres. (**i**) Corticospinal tracts. (**j**) Corpus callosum. (**k**) Superior longitudinal fasciculus. (**l**) Cingulate bundle.

**Figure 7:**
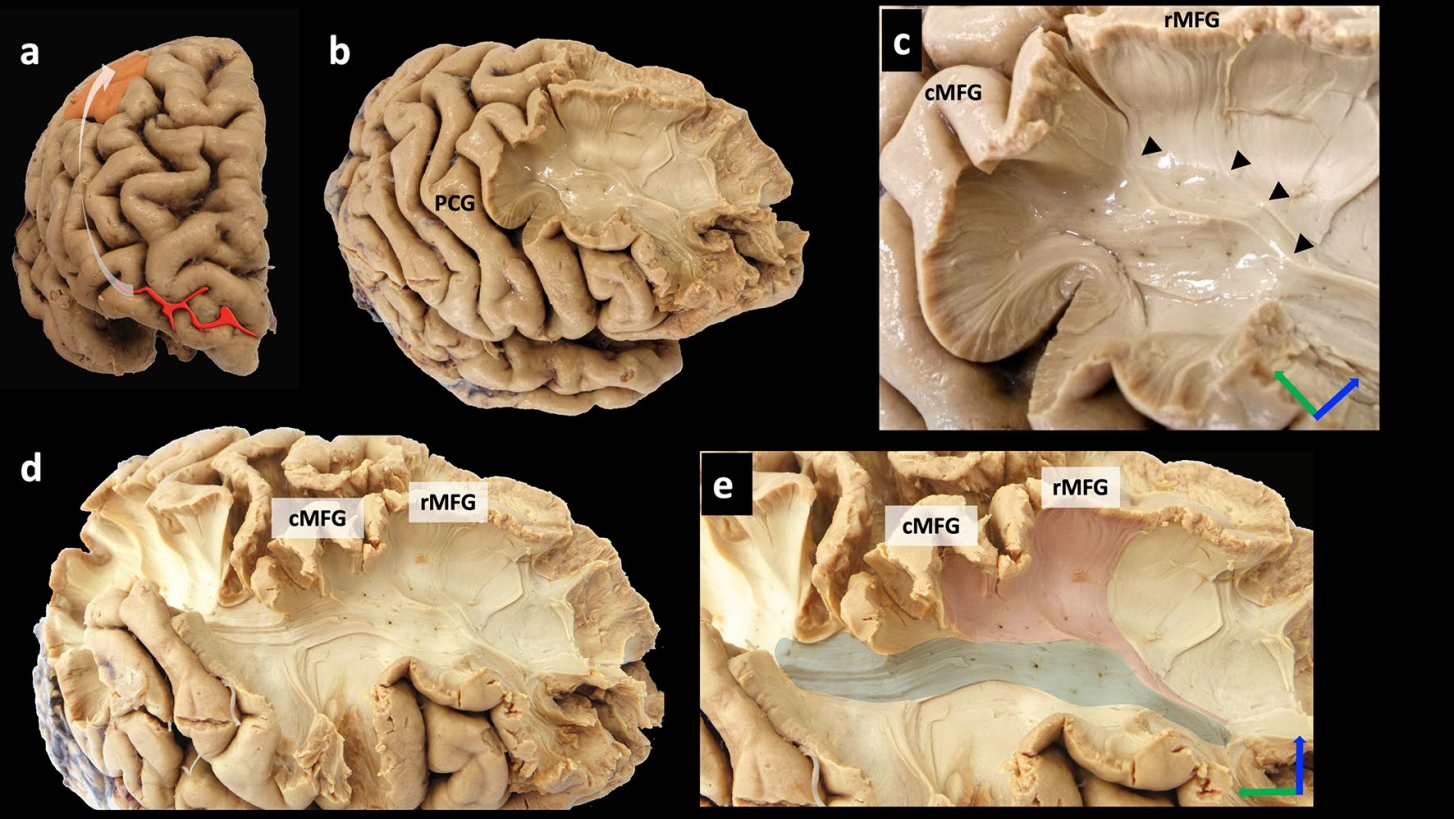
White matter dissection of a right hemisphere (sample 1). (**a**) The dissection line starts posterior to the frontomarginal sulcus (red line) towards the cMFG (orange shade). (**b**) Removing the U-fibres at the MFG reveals the SFLT is running beneath. (**c**) A closer look at the posterior end of the SFLT and how it arises from the MFG cortical region. (**d**,**e**) The spatial relationship between the SFLT (pink shade) and SLF (grey shade) is demonstrated. *Axis: superior (blue arrow); posterior (green arrow).*

A template averaged from a total of 1,021 subjects from the HCP was used to study the spatial relation of the SFLT with adjacent white matter bundles as described in Figs. 5 and 6. The SFLT revealed a complex spatial correlation with several adjacently running fibres (association, projection and commissural). In the left hemisphere, the SFLT is closely and spatially related to the following tracts: (1) the frontal aslant tract (FAT), which combines with the posterior termination of the SFLT at the PMd area (Fig. 5e), (2) the AF, of which the rostral end lies ventral to the PMd segment of the SFLT (Fig. 5f), (3) the inferior front-occipital fasciculus (IFOF), of which the frontal fibres lie medially and inferiorly to the SFLT (Fig. 5g), (4) the anterior projection fibres (APF), which lie medially and inferiorly and also share the same plane with the IFOF (Fig. 5h), (5) the CC, which intermingles with whole SFLT fibres (Fig. 5i), (6) the CST, which is a special case only in the left SFLT average template, where it combines with the dorsal PCG termination (Fig. 5j) and (7) the SLF-II lies caudal and lateral to the posterior termination of the SFLT (Fig. 5k).

In the right hemisphere, the SFLT is closely and spatially related to the following tracts: (1) the FAT, which lies medially and combines with the posterior termination of the SFLT (Fig. 6e), (2) the AF, which is smaller in volume than the left one and lies ventromedial to the SFLT (Fig. 6f), (3) the IFOF, which has the same location as the left side and lies medial and inferior to the SFLT (Fig. 6g), (4) the APF, which also shares the same position as the left side and lies medial and inferior to the SFLT (Fig. 6 h), (5) the CC, which intermingles with whole SFLT fibres (Fig. 6j) and (6) the SLF, which is a special case in the right SFLT due to the significantly larger volume of the SLF (Fig. 6k). The SFLT lies within the rostral SLF extension, especially the SLF-II. Due to the lack of PCG termination in the right SFLT, it has no spatial relation with the CST (Fig. 6i). This study also constructed the cingulate bundle (CB) in both sides to distinguish the SFLT from the CB; in both the template and in all subjects, the SFLT lies laterally to the plane of the cingulate gyrus with both the IFOF and APF between them (Figs. 5 l and 6l).

In addition, there are some differences between the right and left SFLT based on the HCP-1021 average template. The left SFLT has a part that terminates at the dorsal PCG (BA 4/6-caudal) as well as a part that comes from the junction between the SFG and the caudal MFG (cMFG) (BA 6-rostral/8). The right hemisphere has a single bundle with termination posteriorly at the junction between the cMFG and rMFG (BA 8). Both sides share a similar anterior termination in both sides in the anterior part of the rMFG and FP (BA 9/46/10).

Furthermore, the spatial relation of the SFLT with the major white fibre bundles in the 48 subjects is shown in Supplementary Fig. S1, where the SFLT (red) is clearly demonstrated as an independent tract and not a continuation of the SLF-II (blue) or the SLF-III (yellow). In addition, focusing on the SFLT and SLF spatial relation, examples from four subjects are shown in Supplementary Fig. S2. Although they may share the same termination areas, the SFLT and SLF are not shown to be a single bundle.

### Statistical analyses of the GQI indices and the laterality index (LI)

The mean values of the SFLT and its subcomponents’ normalised volumes and the fraction anisotropy (FA) means are shown in Supplementary Table S1. The mean value of the whole normalised volume of the SFLT streamlines in the right hemisphere and the left hemisphere in the whole number of subjects was 0.66% ± 0.39% and 0.63% ± 0.4%, respectively. Regarding the normalised volume’s LI, 14 (29%) subjects had right laterality, 12 (25%) subjects revealed left laterality and 22 (46%) subjects had symmetrical laterality (Supplementary Fig. S3 a).

The mean value of the whole SFLT streamlines’ FA mean in the right hemisphere and the left hemisphere in all subjects was 0.62 ± 0.07 and 0.55 ± 0.22, respectively. Regarding the FA mean’s LI, 6 (13%) subjects had right laterality and 42 (88%) subjects revealed symmetrical laterality (Supplementary Fig. S3b). No subject had a left FA mean laterality. Neither the SFLT nor its subcomponent normalised volume’s LI or FA mean’s LI correlated with the subjects’ gender or handedness groups (p > 0.05) (Supplementary Table S2).

To classify the SFLT into types, the MFG and SFG subcomponents were merged as a single variable since they were likely to share the same BA (Supplementary Table S3). A two-step cluster analysis was conducted based on the presence or absence of a subcomponent with a posterior termination at the BA 4/6-caudal (PCG subcomponent) and the BA 6-rostral/8 (MFG and SFG subcomponents). The outcome was four types of SFLT (as presented in Supplementary Fig. S4: Silhouette measure = 0.9). The SFLT types did not exhibit a correlation with the subjects’ gender or handedness groups (p > 0.05) (Supplementary Table S4).

### White fibre dissection

Based on the prior knowledge of the results obtained using tractography, the dissection was planned first to start from a point posterior to the frontomarginal sulcus and second, to dissect in the direction towards the cMFG (Fig. 7a). After carefully removing the U-fibres in the two right samples, the dissection followed the tracts running rostrocaudally beneath the MFG substances and towards the termination area, which was found to be at the cMFG in the first sample (BA 6-rostral) (Fig. 7c).

**Figure 8:**
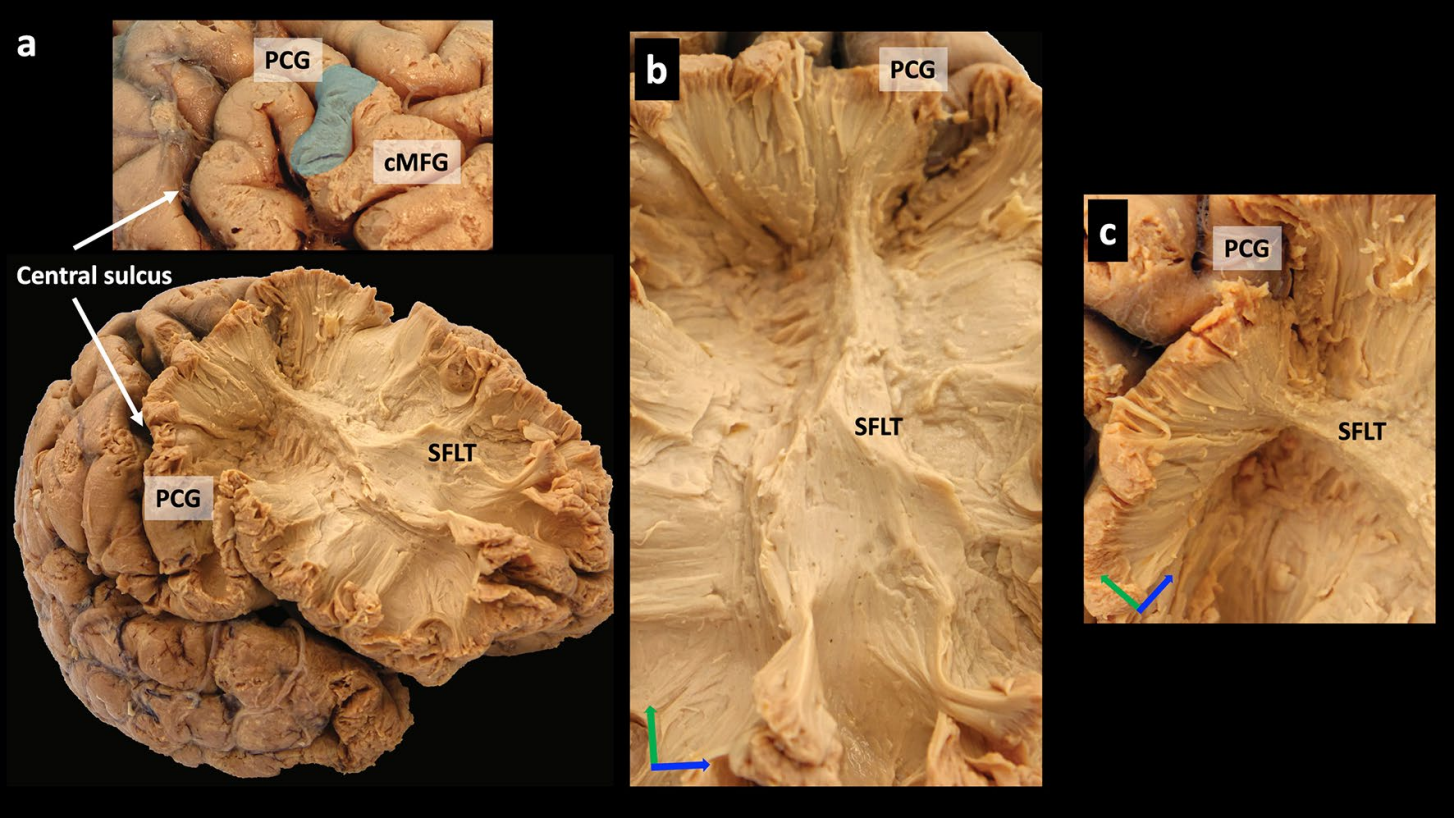
White matter dissection of a right hemisphere (sample 2). (**a**) After dissection of most of the frontal lobe and sparing a limited part of the PCG, the SFLT posterior end is revealed as it arises from the transition zone between the PCG and cMFG (blue shade). (**b**) A closer image showing the entire SFLT from an anterior view. (**c**) A closer image at the posterior end of the SFLT. *Axis: superior (blue arrow); posterior (green arrow).*

Additionally, in this sample, the examination explored the anatomical relationship with the adjacently running SLF and confirmed that each bundle possessed its own separate posterior terminations and that the SFLT in this sample was not a continuation of the SLF (Fig. 7d,e). In the second sample, the dissection was extended posteriorly to reach the PCG. A thin band of white fibres was observed to run from the cortex of the transitional zone between the PCG and cMFG (BA 6-caudal), which curves anteriorly beneath the MFG substances towards the DLPFC and FP (Fig. 8a–c).

## Discussion

The current work extensively studied the anatomy of the SFLT using GQI tractography with a limited white fibre dissection, which focused on the anatomical terminations’ subcomponents and their asymmetry; this aspect, in particular, received little attention in previous reports^4–6^. In all the samples studied, the SFLT was found to be a frontal intralobar tract in distinction from the superficial U-fibres of the sFLS, which differs from some previous reports^5,6^. In their analysis of the short frontal fibres, Catani et al. described the FLS as a chain of U-shaped connections that resemble a prolongation of the SLF connecting the premotor cortex to the DLPFC ^5^. In this study’s opinion, the advantage of processing a multi-shell diffusion image with a higher directional sample (more than 90) and the ability to use modern tractography models (such as GQI) facilitated a better outcome in the tract-rendering process and overcame several potential challenges (such as the effects of crossing and kissing fibres)^8^.

The status of the FLS as more than mere U-fibres was confirmed by the extensive microdissection study recently presented by Komaitis et al.^6^ They confirmed the FLS in all 15 brain samples that were studied, which represents a proportion similar to that achieved in the present study using tractography (100% and 88% of the right and left hemispheres, respectively) (Supplementary Table S1).

**Figure 9:**
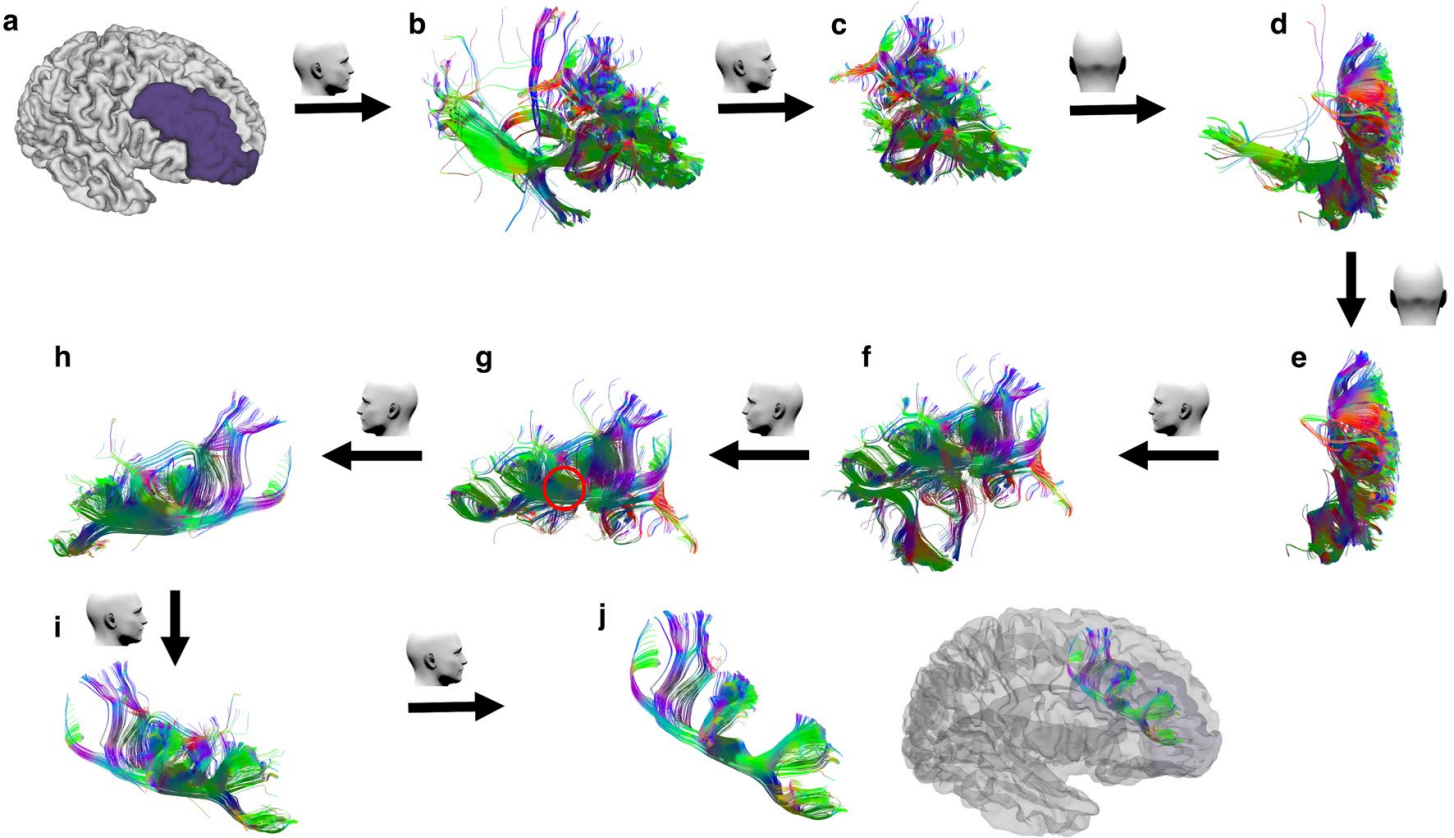
The SFLT rendering steps in the right side using DSI studio. (**a**) The hemispheric white matter ROI is set as a seed region, and the merged rMFG and FP are set as an end region. (**b**) Raw result of the GQI tractography. (**c**–**f**) Irrelevant association, projection and commissural fibres are removed. (**g**) The SFLT can be seen from the medial view running caudal-rostral (red circle). Using the select tool, we focus on this bundle. (**h**–**i**) Remove all remaining U-fibres. (**j**) A completely rendered SFLT.

Furthermore, Komaitis et al. found that in 80% of samples, where a prominent middle frontal sulcus was present, the FLS was encountered just under the most superficial U-fibres at the depth of the sulcus. In the remaining 20% of cases, where the middle frontal sulcus was absent, the FLS was seen to lie just medially to the MFG’s superficial U-fibres. Besides, the FLS fibres were observed to travel at two discrete levels, the sFLS and iFLS, as described by Catani et al.^5^ These descriptions of the relationship of the FLS to the superficial U-fibres correspond with that observed during this study’s tractography process (Fig. 9, and Supplementary Methods file). We confirmed the distinction between the superficial U-fibres of the sFLS and the direct long fibres of the SFLT (Fig. 1, and Supplementary Methods-Fig. S8). The SFLT, when present, lies deep to the sFLS’ U-fibres chain. This finding bears a resemblance to the anatomical findings in the case of the inferior longitudinal fasciculus (ILF) and the occipito-temporal projection system (OTPS), where the ILF represents a longitudinal association fibre running deep to the OTPS’ chain of short U-fibres^11^.

Furthermore, the location of the SFLT was studied in 48 subjects, and the site was mapped in a standard space template (Fig. 2). This study is in agreement with Komaitis et al.; the voxels with the higher frequency of SFLTs are located beneath the MFG’s substances and located antero-lateral to the anterior horn of the lateral ventricles. The constructed tracts mainly shared the same location of the SFLT body but varied in their terminations, especially the posterior part.

The SFLT termination points were studied in the subjects’ space using the semi-automated FreeSurfer Desikan-Killian (DK) atlas as a reference. In agreement with both the spatial location analysis above and the results from the Komaitis et al. study, the present study’s data indicates a similar anterior termination point (in the rMFG and FP at BA 9/46/10). There was a significant variation between subjects regarding the posterior termination points, which were located in one of three regions: the MFG, PCG or SFG (Figs. 3, 4, and Supplementary Table S1). Therefore, regarding incidence, the MFG was the most frequently occurring subcomponent among the three with a right-side preference of 94% compared with an incidence of 79% in the left side. Regarding the SFG subcomponent, a left-side preference with a 60% incidence was observed and detected in 44% of the right hemispheres. Finally, the PCG displayed a left-side preference with a 31% incidence, far less than the other two subcomponents, and was constructed in only 23% of the right hemispheres. Although this study classified these subcomponents according to their termination in the DK atlas, most subjects demonstrated that the MFG and SFG subcomponents were significantly adjacent within one probable zone of the BA (the BA 6-rostral/8). In contrast, the PCG subcomponent was separate and (when available) situated in the BA 4/6-caudal. The BA arrangement was chosen in this study as a variable in the subsequent cluster analysis.

This paper reviewed an already published HCP-1021 average tractography template^12^, which revealed differences in shape between the right and left hemispheres; the left hemisphere’s SFLT was the only one with both the PCG and SFG subcomponents, while the right hemisphere’s SFLT was mainly an exclusive MFG subcomponent. This description corresponds to the incidence rate findings since the PCG and SFG subcomponents revealed a higher incidence in the left hemispheres (Fig. 3, and Supplementary Tables S1, S2). Moreover, the template explored the surrounding white matter, and the SFLT on both hemispheres revealed a close, complex relationship with several white matter bundles (as shown in the results section and Figs. 5 and 6). The major difference between both hemispheres in this aspect was the relationship between the PCG subcomponent and the CST (which was absent for the right hemisphere), and the relationship between the right SFLT (the MFG subcomponent) with the SLF (which was absent for the left hemisphere) in the template example. In 20% of the hemispheres studied by Komaitis et al., the SLF and FLS were recorded as two completely distinct tracts in a ratio far lower than that discovered by the present study since it was possible to construct the SFLT in 100% of the right hemispheres. Komaitis et al. reported that, in most (80%) cases, they identified the FLS as the anterior extension of the SLF’s fibre system. In contrast to these results, all the SFLTs constructed in this research were located within the frontal lobe and shared no termination pattern like the familiar ones usually found in the SLF (Supplementary Figs. S1 and S2, and Supplementary Methods-Fig. S10 )^13,14^.

The SFLT (as a whole) or its subcomponents demonstrated no specific laterality in either normalised volume or FA mean values, and most subjects possessed symmetrical organisation. One exception was the MFG subcomponents’ normalised volume LI (Supplementary Table S2). The latter showed 44%, 21% and 17% of subjects with a right-side, left-side, and symmetrical LI, respectively. A cluster analysis was undertaken to reveal any possible SFLT subtypes based on the laterality of these subcomponents. After rearranging them according to their probable BA, four different types were identified (shown in Supplementary Fig. S4); 39.6% of the population was projected to have a bilateral SFLT with a posterior termination from BA 6-rosrtal/8 (Type 1) only, while 12.9% were assumed to possess only a unilateral right side tract with the same termination (Type 3). Types 2 and 4 had a bilateral BA 6-rosrtal/8 and a unilateral BA 4/6-caudal subcomponent in the right and left hemispheres, respectively. No subtype expressed the co-existence of all the BA subcomponents bilaterally. Furthermore, we analysed if there was an asymmetrical distribution of the SFLT’s normalised volume LI, FA LI and its four types in the gender and handedness groups. Neither LI indices nor the SFLT’s types exhibited a correlation with either gender or handedness (Supplementary Tables S2 and S4).

This study concluded with a limited white matter dissection. The SFLT was located in both right hemispheres’ samples, and their posterior terminations were as follows: one at the MFG (BA 6-rostral/8) in the first sample (Fig. 7) and another at a zone between the PCG and cMFG (BA 6-caudal) (Fig. 8). In the first sample, the distinctiveness of the posterior terminations between the SFLT and the adjacently running SLF was confirmed.

Regarding the BA subcomponents, there are limited similarities between this study’s tractography results (incidence in subjects) and the white fibre dissection study (incidence in hemispheres) done by Komaitis et al. The right BA 6-rostral/8 subcomponent was found in 100% of cases in both studies. The remaining BA distribution did not show other similarities and can be seen in Supplementary Table S3.

Based on this study’s findings and previously published research, the SFLT represents a connection between the DLPFC and PMd cortex. The BA4/6-caudal subcomponent seems to exist in humans at a much lower rate than BA6-rostral/8, which might explain why other similar studies in humans could not confirm its existence^4^ ; however, such an interpretation should be made with caution since no direct connection between the caudal PMd (PMd proper) and DLPFC was detected in animal studies^1,15^. The available data concerning a human direct structural pathway between the rostral PMd and DLPFC are still limited, apart from a few functional studies involving healthy participants^3^, well-recovered chronic stroke patients^4^ and postmortem examinations^6^. Regarding the motor network, the DLPFC has been reported as primarily connected to the PMd area, PMv and SMA. Brain-tracing studies in animals have revealed similar connections^1,2,15^.

Regarding the SFLT’s possible functional rule, crucial structures for motor and cognitive skills are concomitantly activated during motor and cognitive tasks, including the PMd and DLPFC^16,17^. The activation of these brain structures is stronger in more difficult (as opposed to easy) tasks^16^. When performing a difficult (i.e., complex) motor task, higher-order cognitive resources such as executive tasks are required to perform the action successfully. Furthermore, the rostral PMd (pre-PMD) and DLPFC may contribute to executive function through sequence generation and attentional selection, respectively, and the functional coupling between them seems to play a pivotal role in integrating these executive processes^18^. Therefore, the SFLT might serve as the structural backbone in these functions. Additional studies involving functional connectivity and task-related functional MRI (fMRI) analysis regarding the SFLT’s spatial correlation may be necessary to confirm this assumption.

As demonstrated in this work, the superior segment of FLS possesses a deeper longitudinal segment, representing a direct connection between the PMd and DLPFC; consequently, it is proposed that this connection is named the superior frontal longitudinal tract (SFLT) while preserving the term ‘superior frontal longitudinal system’ (sFLS) for the superficial chain of U-fibres, representing the indirect connection between the PMd and DLPFC (Fig. 1, and Supplementary Methods-Figs. S7, S8).

Some limitations of the present study are considered below. A few of the constructed SFLTs were significantly shorter and lacked a BA 6 termination, but based on their spatial location, they were identified as SFLT rather than being discarded (subjects’ number highlighted in yellow in Supplementary Fig. S1). Additionally, six subjects who had a unilateral SFLT comprising a chain of two bundles (subjects’ number highlighted in red in Supplementary Fig. S1) were also included as the SFLT in this study since they shared the same spatial location and were positioned deeper than the sFLS’ U-fibres; also, these chains were in most instances significantly longer than the superficial U-fibres connecting non-adjacent gyri. Of these six subjects, only one (subject number 20) had this pattern on both hemispheres.

Furthermore, the current work focused only on the SFLT and ignored the iFLS, as it was assumed that multiple dissimilarities might require a separate study. Moreover, the reason for a much lower rate of the BA4/6-caudal subcomponent is not clear to us, and factors related to the tractography methodology and concepts cannot be excluded. Despite improvement in fibres tractography methods, there is still no gold standard to define the exact anatomy of fibre tracks in the human brain. We did not investigate the effects of seeding strategy, step size, angular threshold or interpolation method, although they may affect the performance of a tracking algorithm in regard to the streamlines volume and numbers^19^. Virtual dissection of the SFLT was not a straightforward process from the beginning since it lies within the vicinity of many other crossing bundles, including the CC, FAT, IFOF, AFP and especially the sFLS’ chain of U-fibres lying superficially (Figs. 1 and 9, Supplementary Fig. S1, and Supplementary Methods-Fig. S8). These groups of fibres make the process of dissection a bit challenging, specifically in the case of cadaveric white matter studies. Finally, the current work focused on the structure of the SFLT and did not include any functional imaging analysis.

In conclusion, this study demonstrated that the SFLT is a frontal intralobar tract connecting the PMd to the DLPFC and is not a mere continuation of the SLF. The tract exhibited variable patterns in its posterior terminations among the samples. It revealed a close, complicated spatial relationship with several white matter bundles, including the FAT, AF, IFOF, CST, CC and SLF.

## Materials and methods

### The dataset

Pre-processed MRIs of 48 healthy young adults, which included structural T1-weighted and diffusion-weighted images from the HCP database were imported. The subjects were 23 females (47.9%), 25 males (52.1%), 20 left-handed (41.7%) and 28 right-handed (58.3%) with a mean age of 28.19 ± 4 years (range 22–35 years).

The multi-shell diffusion scheme was collected on a 3 T scanner with a 64-channel, tight-fitting, brain-array coil. The b-values were 1000, 2000 and 2995 s/mm^2^. The number of diffusion sampling directions were 90, 90 and 90, respectively. The in-plane resolution was 1.25 mm, and the slice thickness was 1.25 mm^20^.

### GQI tractography

Using GQI tractography, the diffusion data were reconstructed with a diffusion sampling length ratio of 1.25^7^. A deterministic, fibre-tracking algorithm was also applied^19^. Tractography was conducted using the DSI studio software tool (https://dsi-studio.labsolver.org), and tractography seeds were created using the semi-automated FreeSurfer DK cortical atlas^21^. The atlas for each subject was imported from the HCP database together with the images. The ‘seed’ region was created by either the left or right cerebral white matter^22^, with a setting of 10 fixed seeds per voxel^23^. The ‘end’ region was placed on the same side of the seed and was created by merging the rMFG and FP into a single region (Fig. 9 a, and Supplementary Methods-Fig. S1a). Minimum length of streamlines was selected based on the assumption that U-fibres lies in the range between 3 to 30 mm^24^. Thus, we chose 40 mm as the minimum length to eliminate the short fibres. The normalised quantitative anisotropy threshold was set to 0.075, and the angular threshold was set to 45 degrees. Fibre trajectories were smoothed by averaging the propagation direction with 20% of the previous direction.

After confirming the parameters mentioned above, and initiating the streamlines reconstruction process, the whole streamlines that originate from the white matter ‘seed’ region and terminate in the merged rMFG/FP ‘end’ region are displayed (Fig. 9b). Then, non-relevant association, projection and commissural fibres are manually eliminated (Fig. 9c–f). The remaining bundles were approached from the ventro-medial side; using the select tool in the DSI studio software the streamlines located underneath the superficial U-fibres were selected. The target streamlines (non-U fibres) were observed running in a rostro-caudal fashion from the premotor and, sometimes, the motor regions (PCG, the posterior parts of both SFG, and the cMFG) to the DLPFC (Fig. 9g—red circle). Last, the short U-fibres were eliminated using the ‘select’ and ‘remove’ tools to reconstruct the SFLT (Fig. 9j).

An example of a detailed SFLT’s streamlines virtual dissection and the elimination of the remaining short streamlines methods is provided in the Supplementary Methods file.

After confirming the relevant streamlines’ structure, relevant indices were obtained using the DSI studio software tool, including the streamline volume and the diffusion tensor imaging FA means. A streamline’s normalised volume was added by calculating the streamline volume percentage of the whole white matter volume.

### Spatial location

Construction of a graphical group overlap images can be used to support group-level inferences concerning the number of participants in an experiment. Using this approach, a group of binary images that can be summed is then overlaid on an standard anatomical template, allowing visualization of how many subjects showed the structure of interest at each location. The overlapping process will cause some voxels to have a higher frequency of SFLTs than others. These differences in voxel frequency can be displayed by a single image known as the overlap image using a color palette.

The constructed SFLT’s streamlines were converted to 3D regions of interest (ROIs) using the DSI studio’s tract-to-ROI tool. Then, they were registered to a standard space and converted to binary masks using the FSL package (FSL, The University of Oxford, Oxford, UK). Next, an overlap image was created using the MRIcron software (www.nitrc.org/projects/mricron, University of Nottingham School of Psychology, Nottingham, UK), with all subjects’ SFLT data plotted over a standard MNI template to view common spatial locations and frequencies. The generated image was displayed with the proper colour map using the connectome workbench software (Washington University in St. Louis and associated consortium institutions, https://www.humanconnectome.org/).

### SFLT subcomponents

The constructed streamline termination points were examined for each subject in the native space using the DK atlas. Accordingly, the SFLT was broken down into subcomponents based on these termination points.

### Spatial relations with adjacent white matter bundles

To study the spatial relationship of the SFLT, a virtual dissection of the brain’s streamlines generated from an already published HCP-1021 template using DSI studio was conducted in the 48 subjects’ images. The HCP-1021 template was averaged from a total of 1,021 subjects from the HCP data^12^. The construction methods of the SLF and AF were already described by Wang et al.^13^ The construction of the remaining white fibre bundles was made by selecting different ROI’s masks from the Freesurfer DK Atlas. The FAT was constructed by selecting the fibres running between the caudal parts of both the SFG and IFG. The IFOF was constructed by selecting the frontal lobe as an ROI seed and two other ROI masks to select fibres that passed from the frontal lobe rostrally as described by Wu et al.^25^ The APF was constructed by selecting fibres running between the frontal lobe and the ipsilateral thalamus and basal ganglia ROI. The callosal commissure was constructed by selecting fibres running through the corpus callosum ROI. The CB was constructed by selecting fibres running through the cingulate cortex ROI. The frontal CST was constructed by selecting fibres running between the PCG and brainstem ROI.

### The laterality index (LI)

The LI of the SFLT normalised volume and FA mean was calculated using the equation $\frac{(left - right)}{(left + right)}$. Values between 1 and 0.2 were considered to be left-sided, those between − 0.2 and − 1 were right-sided and values below 0.2 and above − 0.1 were symmetrical^26^.

### Statistical analyses of GQI indices and the LI

Descriptive data are expressed as mean ± standard deviation. A Chi-squared test or Fisher’s exact probability test was applied to assess the correlation of the LI patterns (right, left and symmetrical) of the SFLT normalised volume and FA mean with the subjects’ gender and handedness groups. Using a two-step cluster analysis method, the SFLT was categorised according to the occurrence of its subcomponents on both sides. The Fisher’s exact test assessed the correlation of the SFLT types with the subjects’ gender and handedness groups. Significance in all tests was set at *p* < 0.05. Statistical analyses were conducted using SPSS (version 25.0) and online statistical software from VassarStats (https://vassarstats.net).

### White fibre dissection

With the prior knowledge of the GQI tractography results, this study conducted limited brain white fibre dissections. All brain samples were prepared and fixed according to the method described by Klingler et al.^27^ Two right hemispheres from anonymised donors were donated by the Department of Neuroanatomy at Fukushima Medical University, Japan.

### Ethical standards

The experiment protocol involving humans’ brain and the waiving of informed consent due to the nature of the study were approved by the local Ethics Committee of the Fukushima Medical University (number 2137, December 17th, 2018), which is in accordance and guided by local institutional policy, the Japanese national law, and the World Medical Association Deceleration of Helsinki.

## Supplementary information

Supplementary Figures.

Supplementary Methods.

Supplementary Tables.

## Data Availability

The data sample was imported from the Human connectome project database.
