# Supplementary material for: The superior frontal longitudinal tract: a connection between the dorsal premotor and the dorsolateral prefrontal cortices
Source: Sci Rep. 2020 Sep 28;10:15855. doi: 10.1038/s41598-020-73001-7 (PMC7522085; doi:10.1038/s41598-020-73001-7)

The superior frontal longitudinal tract: a connection between the dorsal premotor and the dorsolateral prefrontal cortices

Supplementary figures

Mudathir Bakhit[1, *], Masazumi Fujii[1], Ryo Hiruta[1], Masayuki Yamada[1], Kenichiro Iwami[2] Taku Sato[1], Kiyoshi Saito[1]

[1]Department of Neurosurgery, Fukushima Medical University, 1 Hikarigaoka, Fukushima, 960-1295, Japan.

[2]Department of Neurosurgery, Aichi Medical University, 1-1 Yazakokarimata, Nagakute, Aichi, 480-1195, Japan.

***Corresponding author:**

Mudathir Bakhit

Department of Neurosurgery

Fukushima Medical University

Fukushima city- Japan

Tel: +81-24-547-1268

Fax: +81-24-548-1803

Email: m-bakhit@fmu.ac.jp

Supplementary Fig. S1: SFLT and white fibre bundles spatial correlations.

- A red highlighted subject number denotes an SFLT made of a chain of two bundles. Subjects number 20 was the only one having this pattern on both hemispheres.

- A yellow highlighted subject number denotes the lack of posterior termination in the PMd (BA 6) in both sides.

- The commissural fibres were omitted for the sake of visualization of other bundles.

White fibre bundles

Superior frontal longitudinal tract (SFLT)	Superior longitudinal fasciculus II (SLF-II)
Superior longitudinal fasciculus III (SLF-III)	Arcuate fasciculus (AF)
Inferior fronto-occipital fasciculus (IFOF)	Anterior projection fibres (APF)
Frontal aslant tract (FAT)	Corticospinal tract (CST)

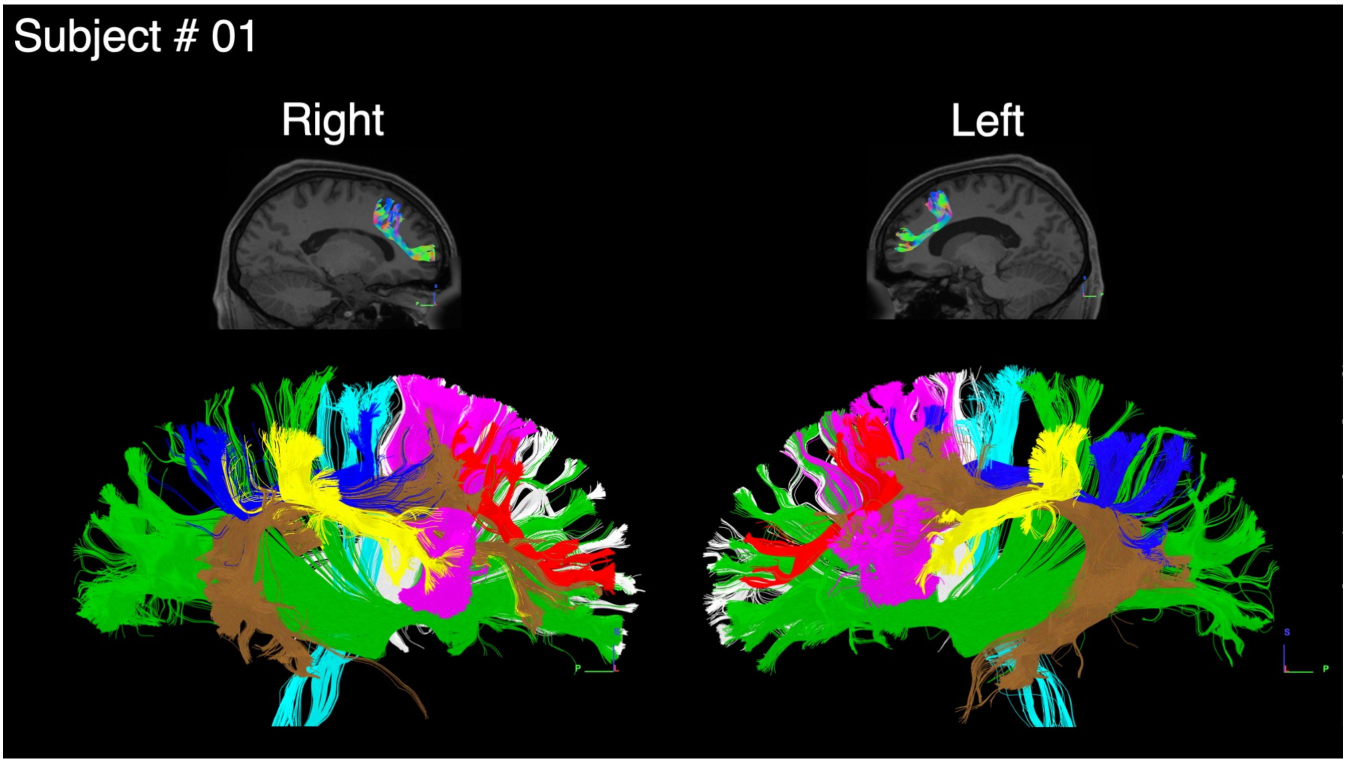
Subject # 01
Right
Left

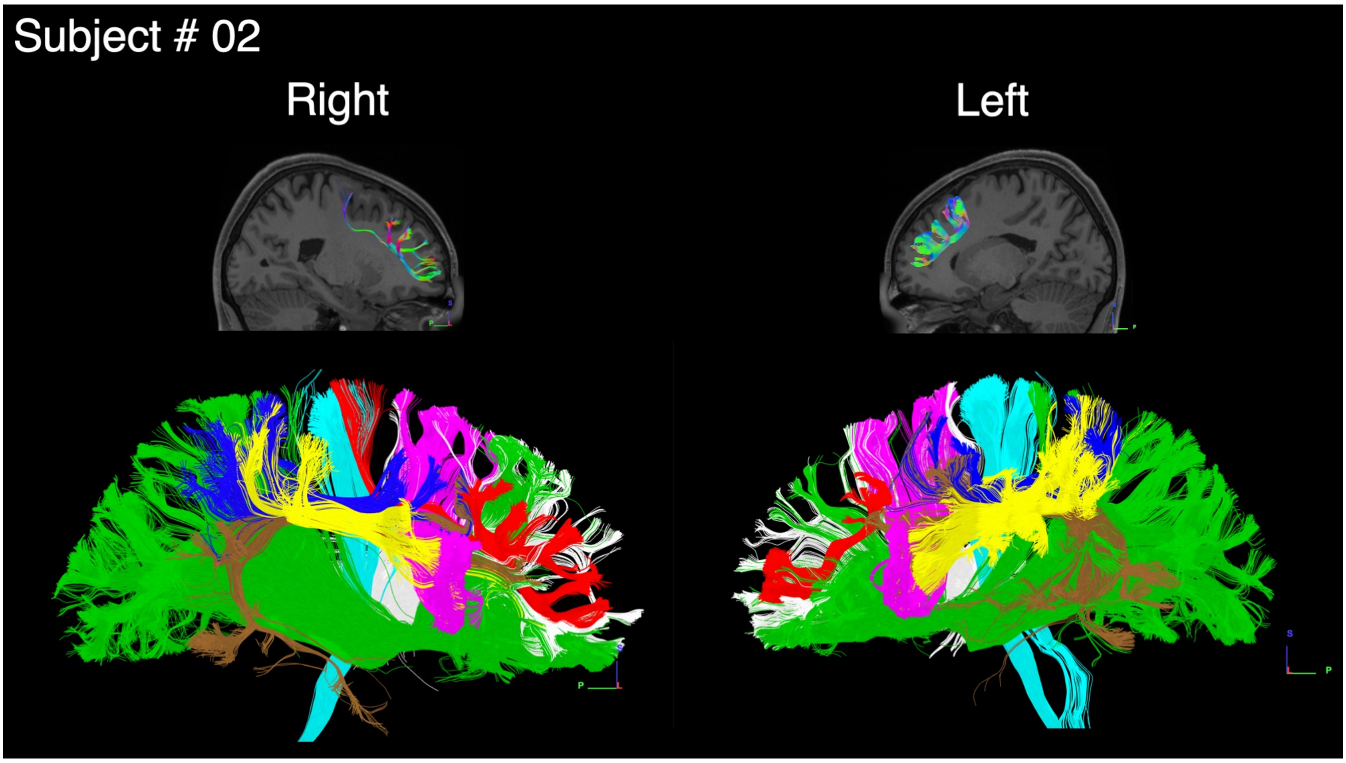
Subject # 02
Right
Left

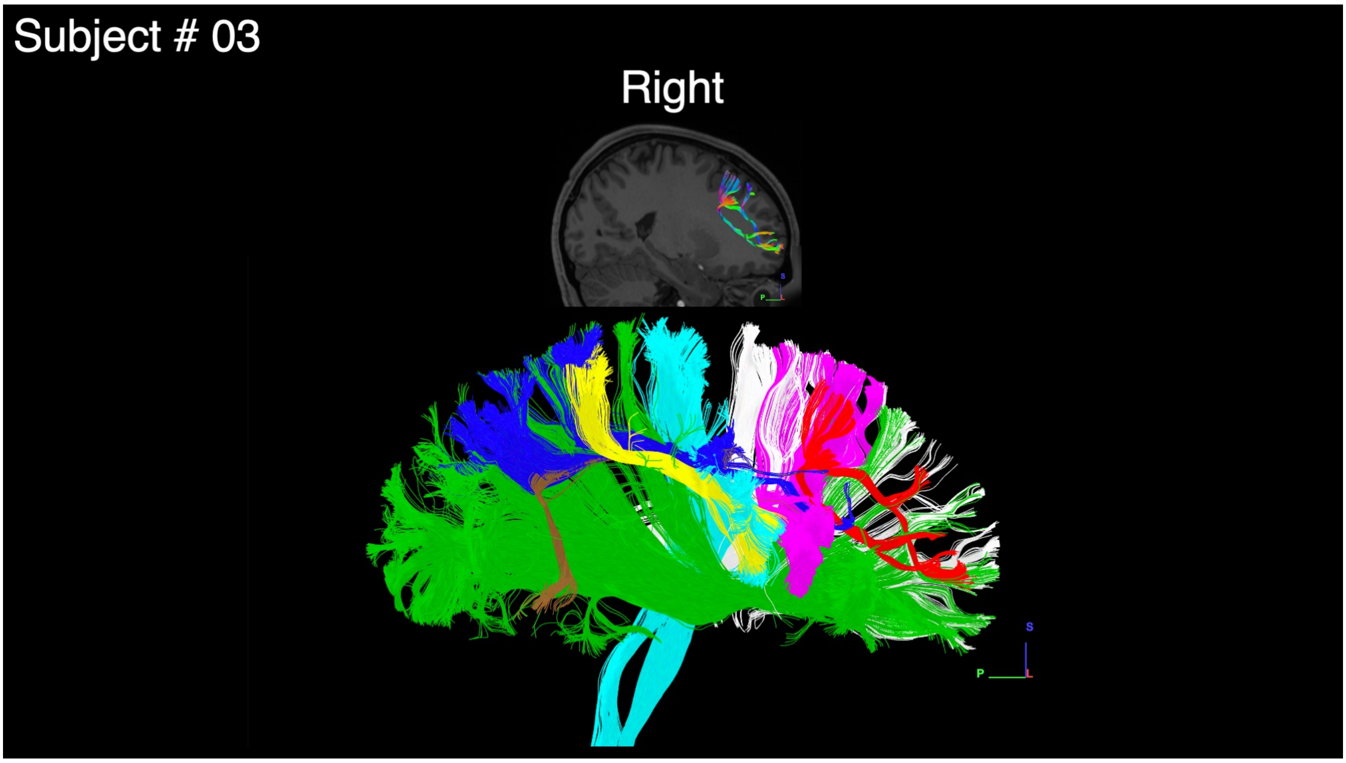
Subject # 03
Right

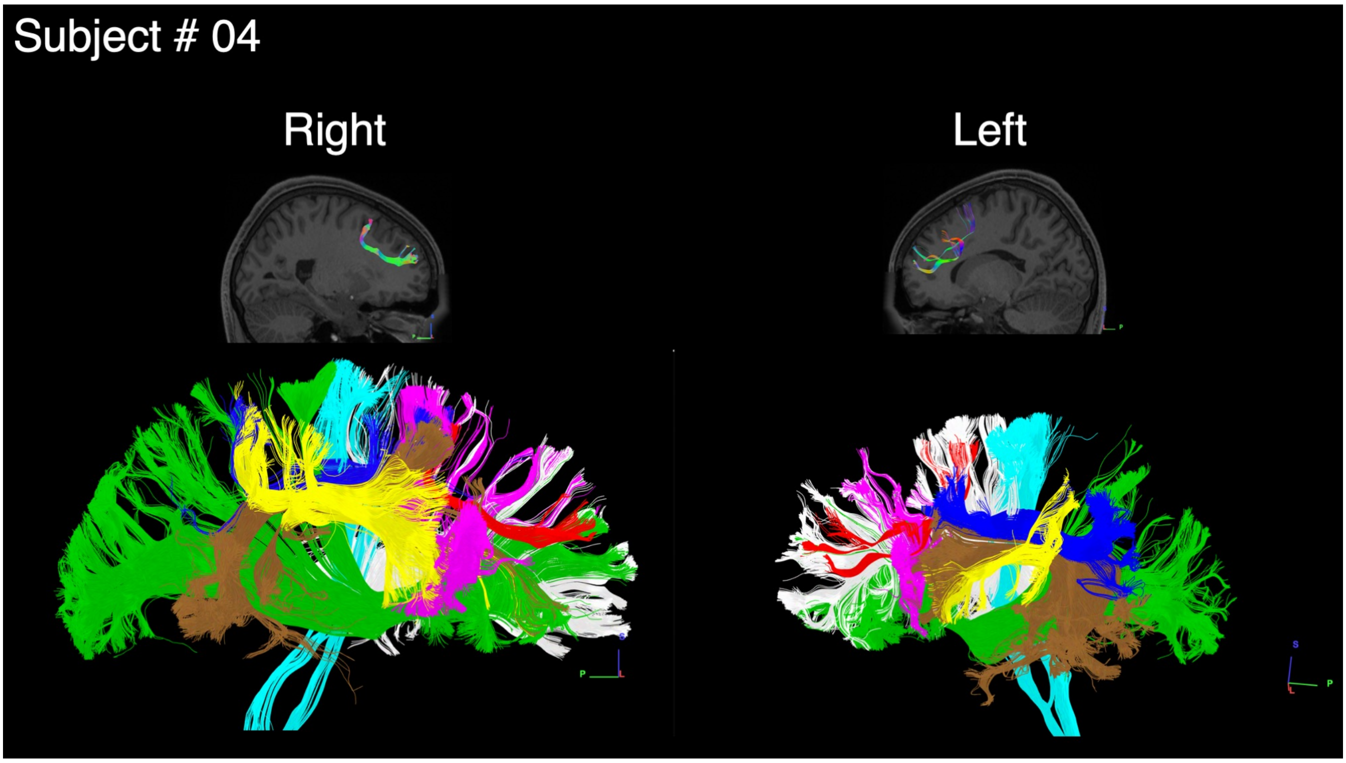
Subject # 04
Right
Left

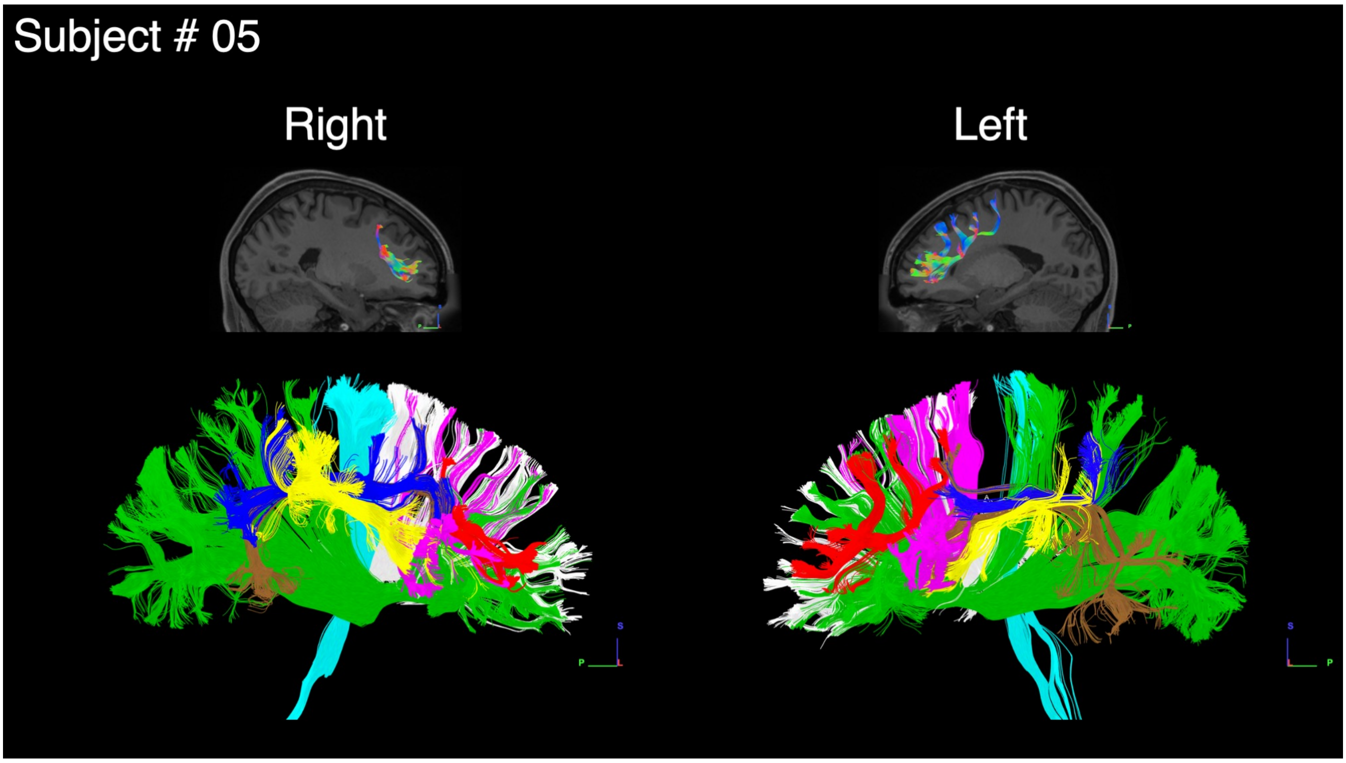
Subject # 05
Right
Left

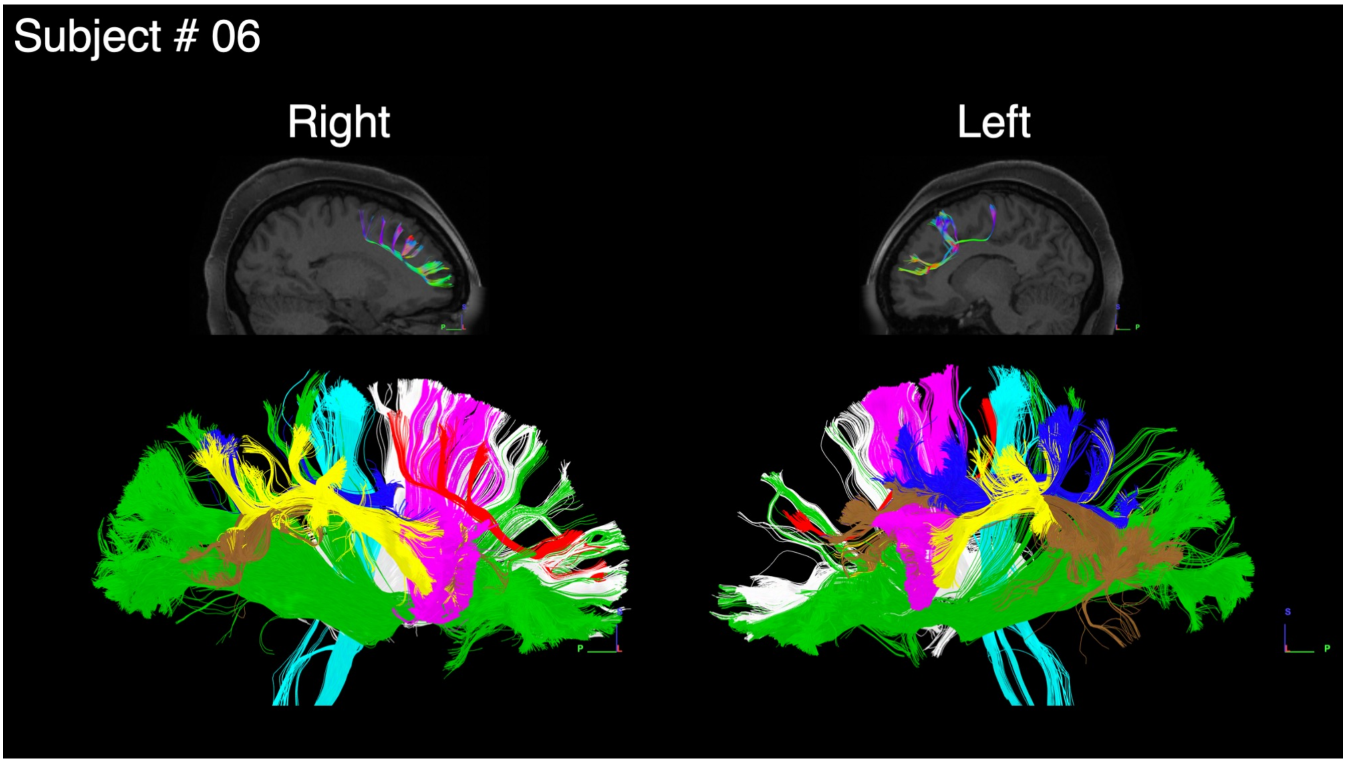
Subject # 06
Right
Left

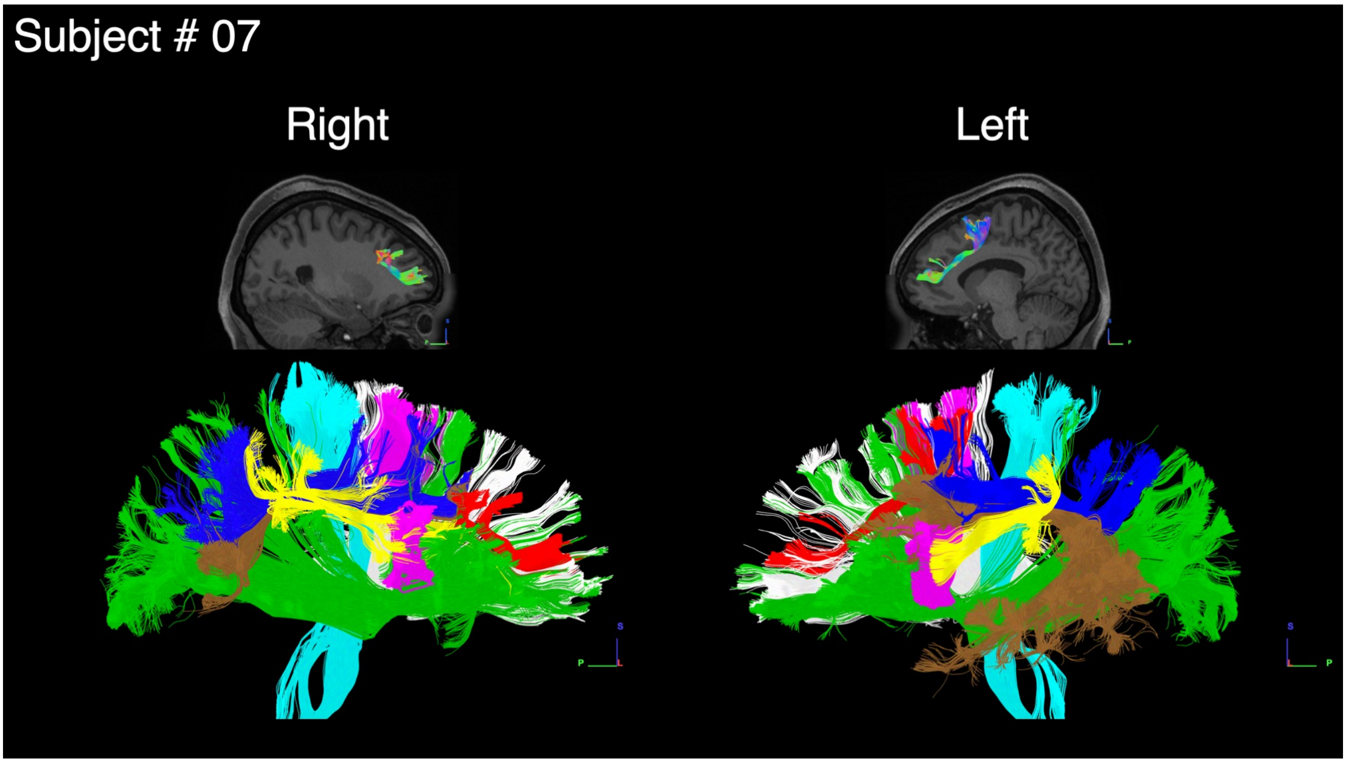
Subject # 07
Right
Left

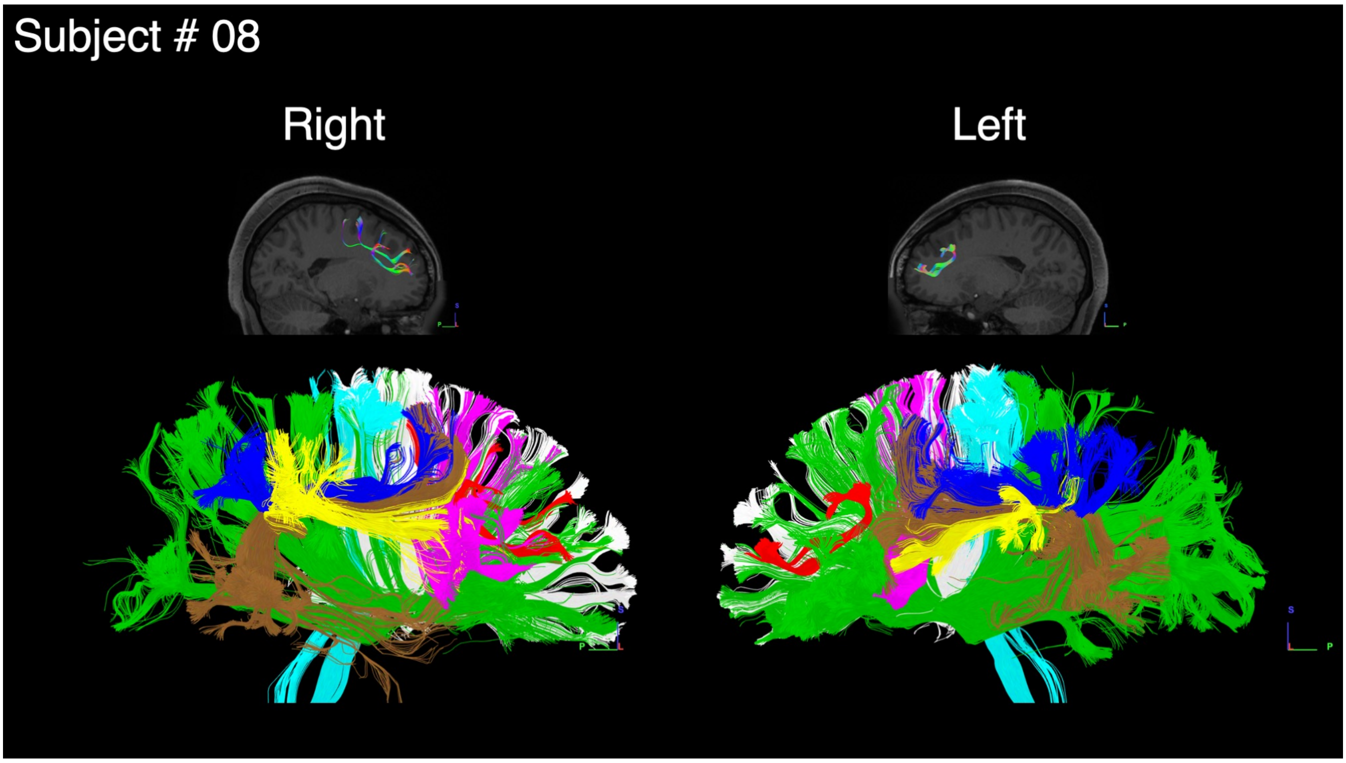
Subject # 08
Right
Left

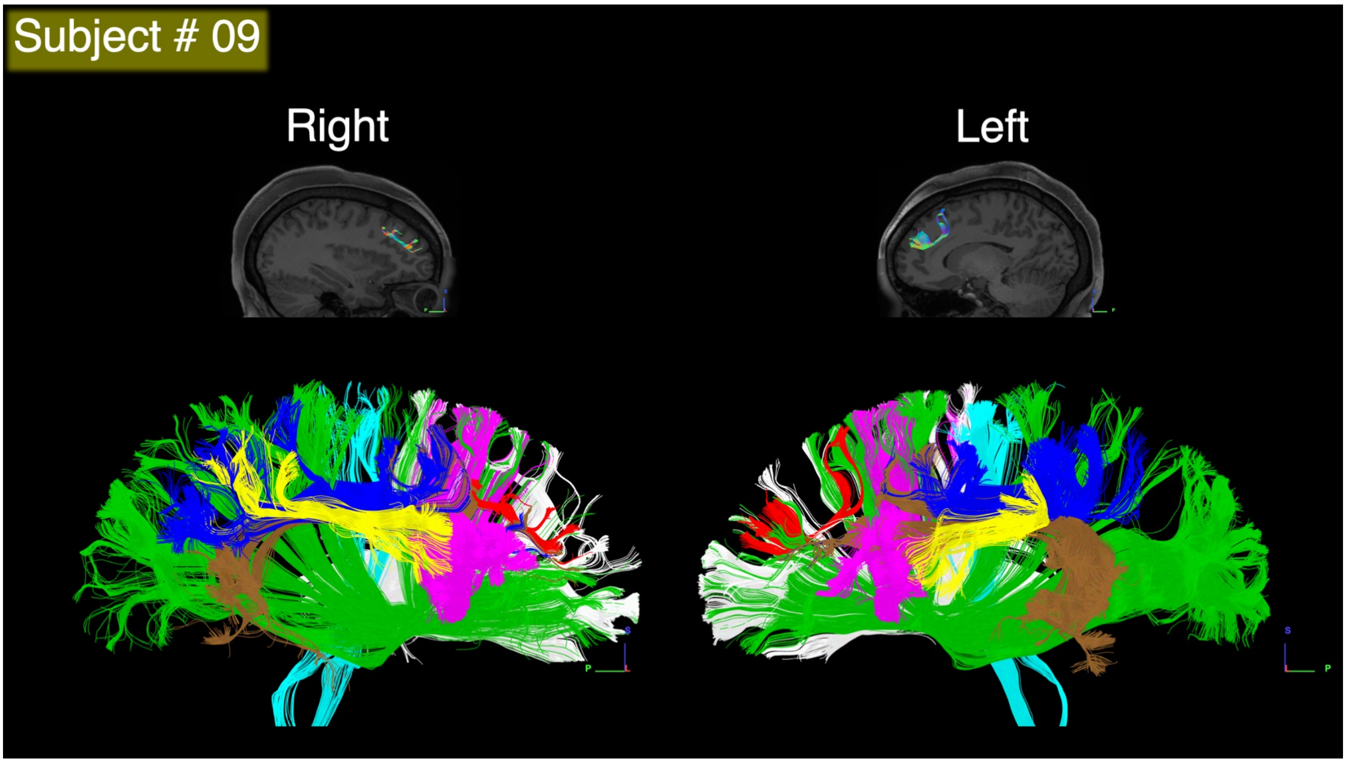
Subject # 09
Right
Left

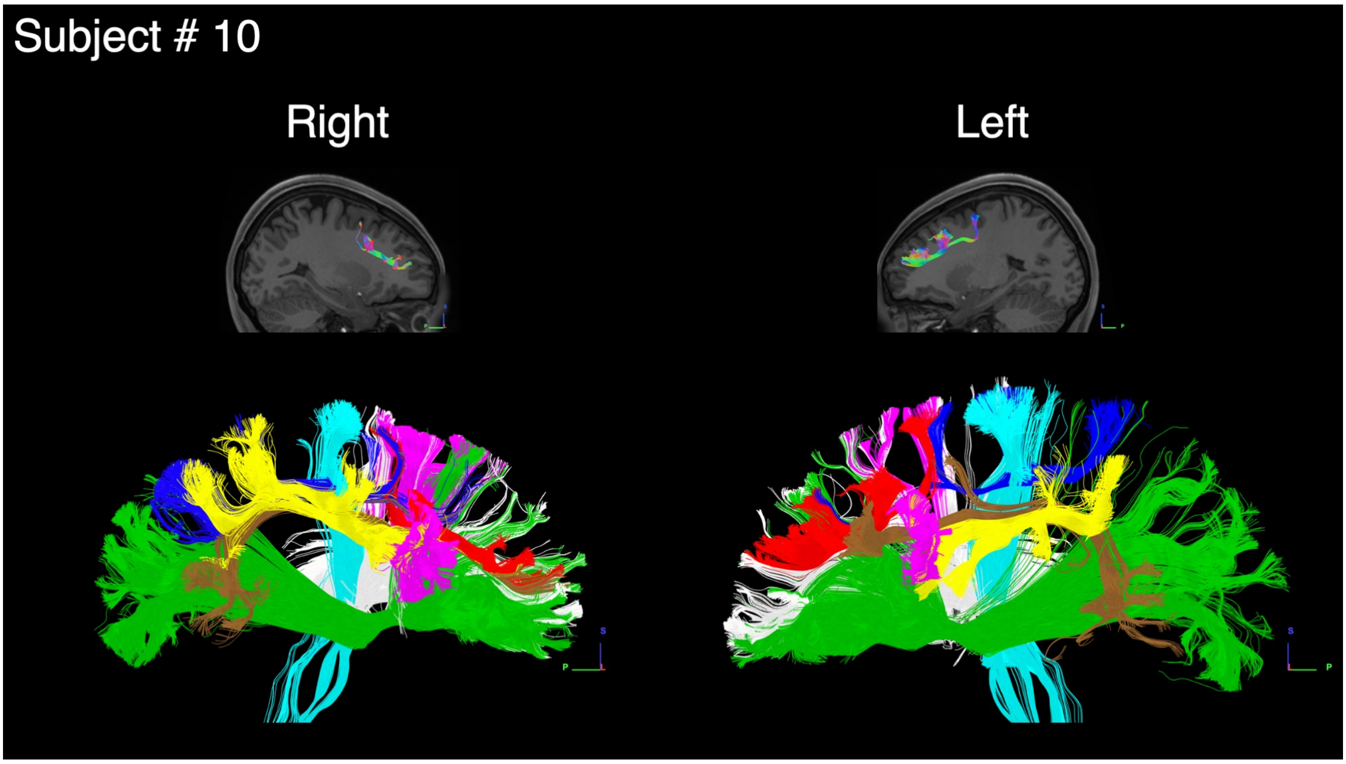
Subject # 10
Right
Left

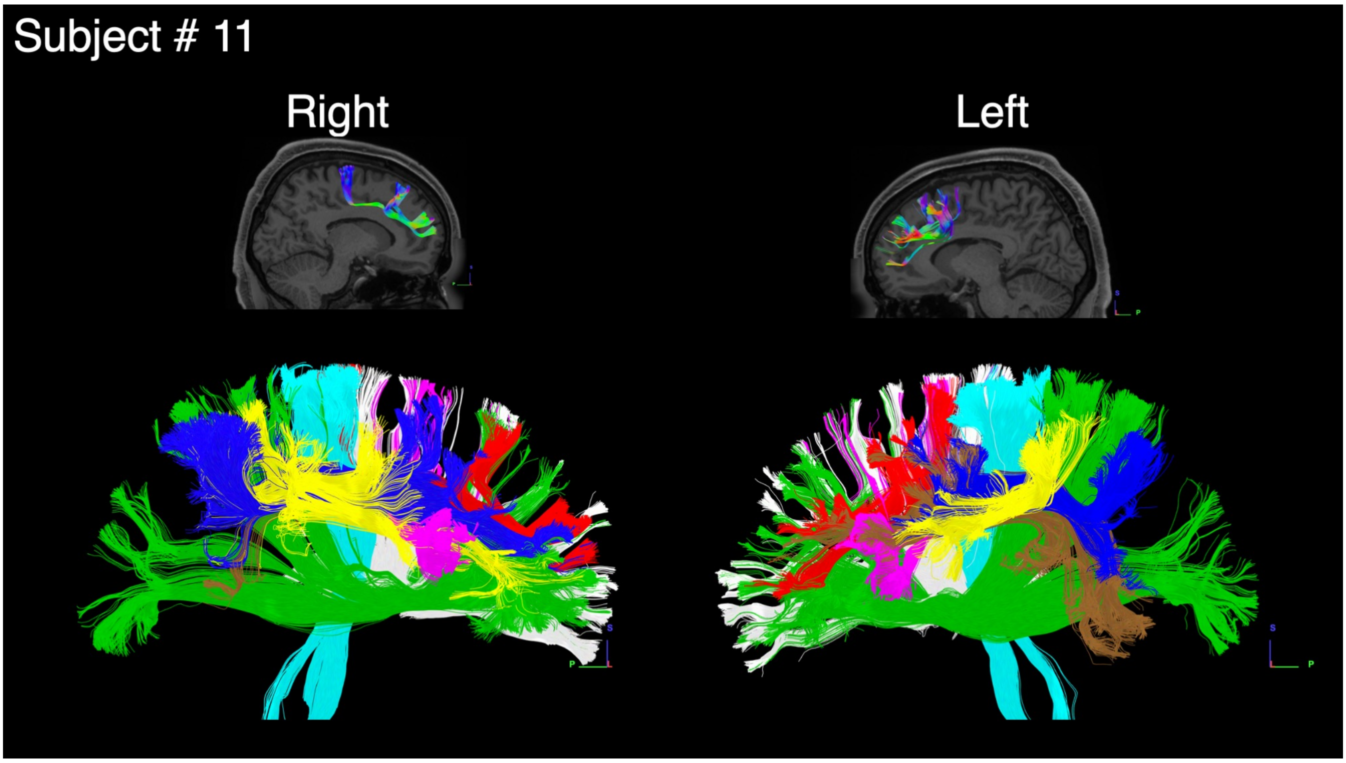
Subject # 11
Right
Left

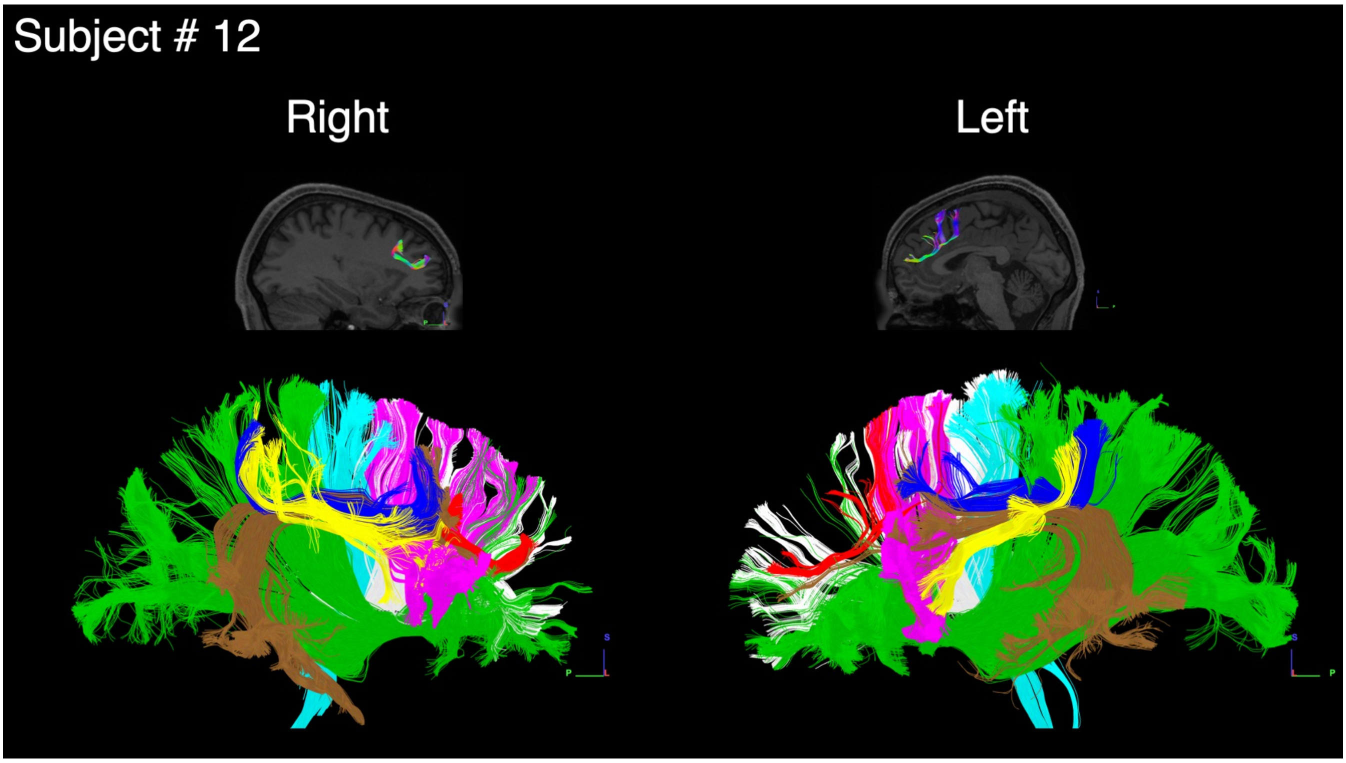
Subject # 12
Right
Left

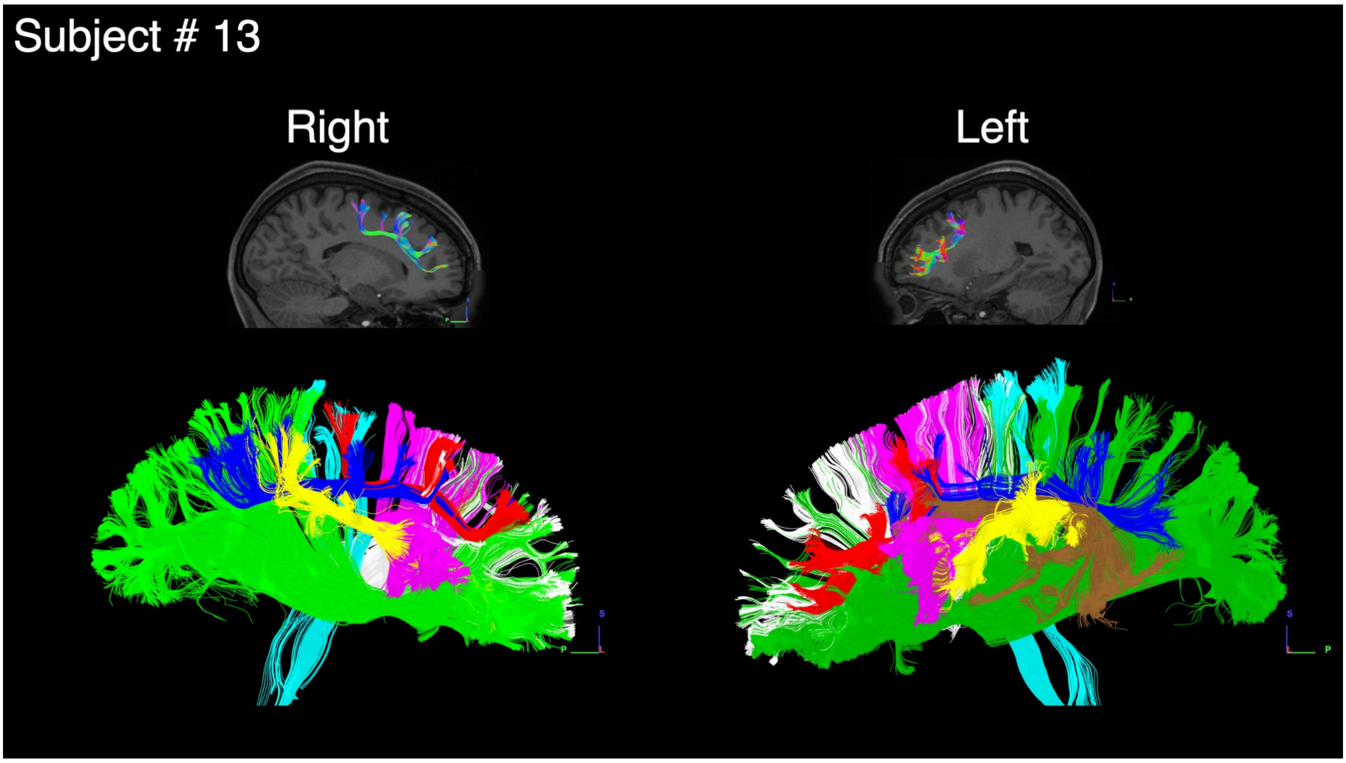
Subject # 13
Right
Left

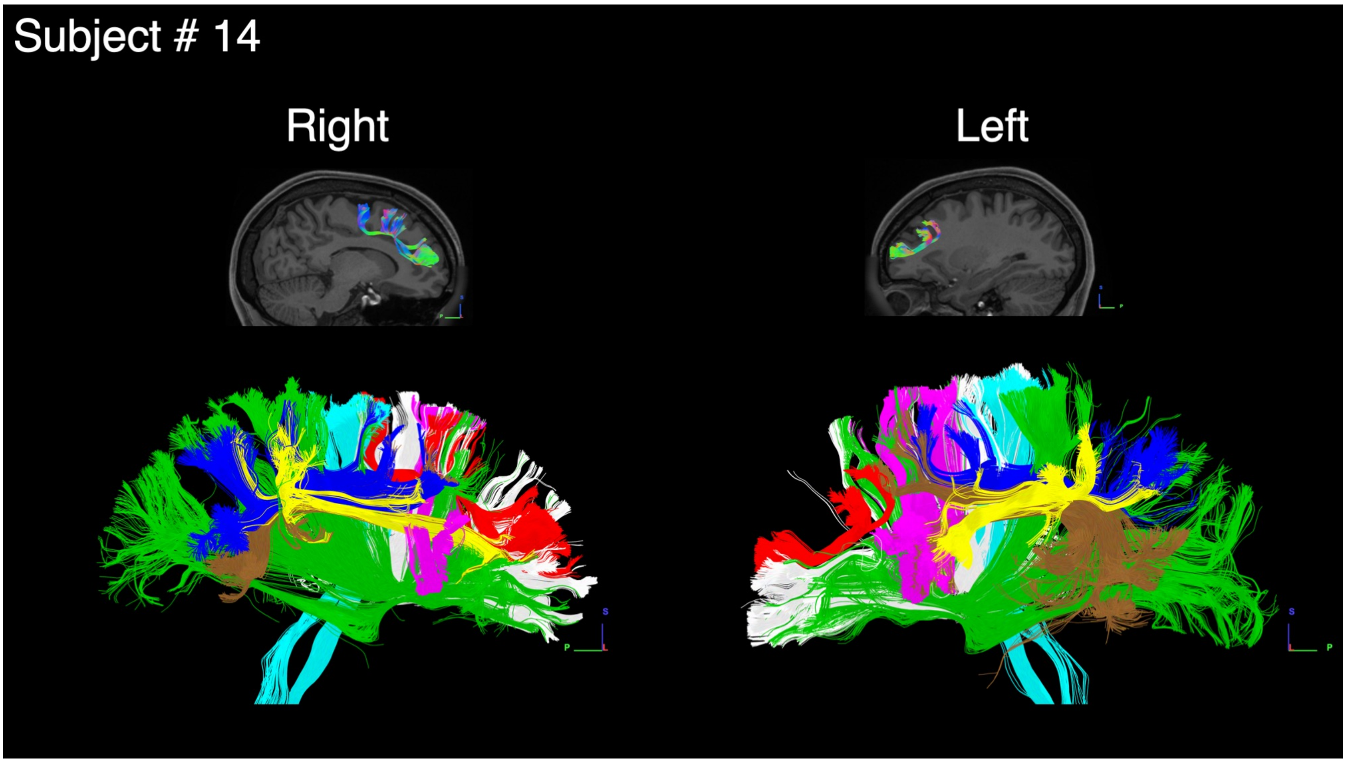
Subject # 14
Right
Left

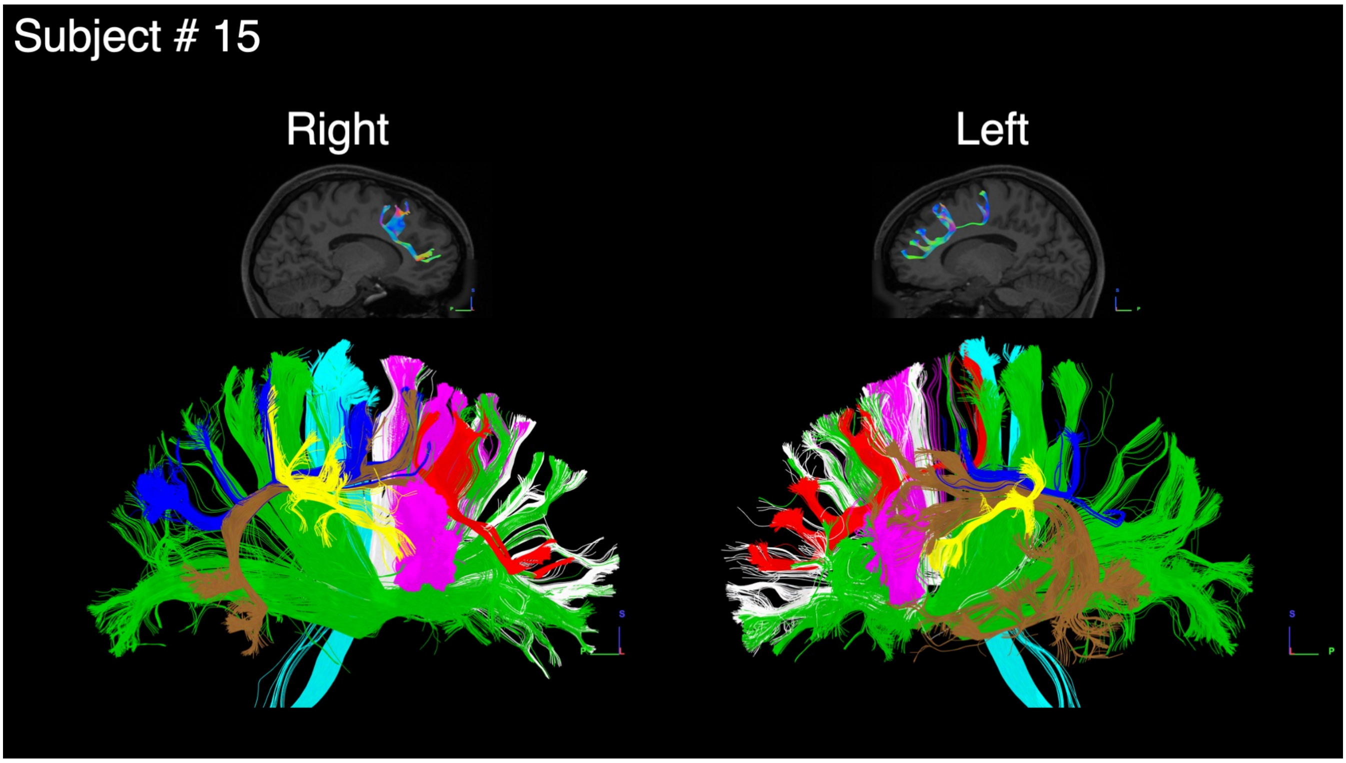
Subject # 15
Right
Left

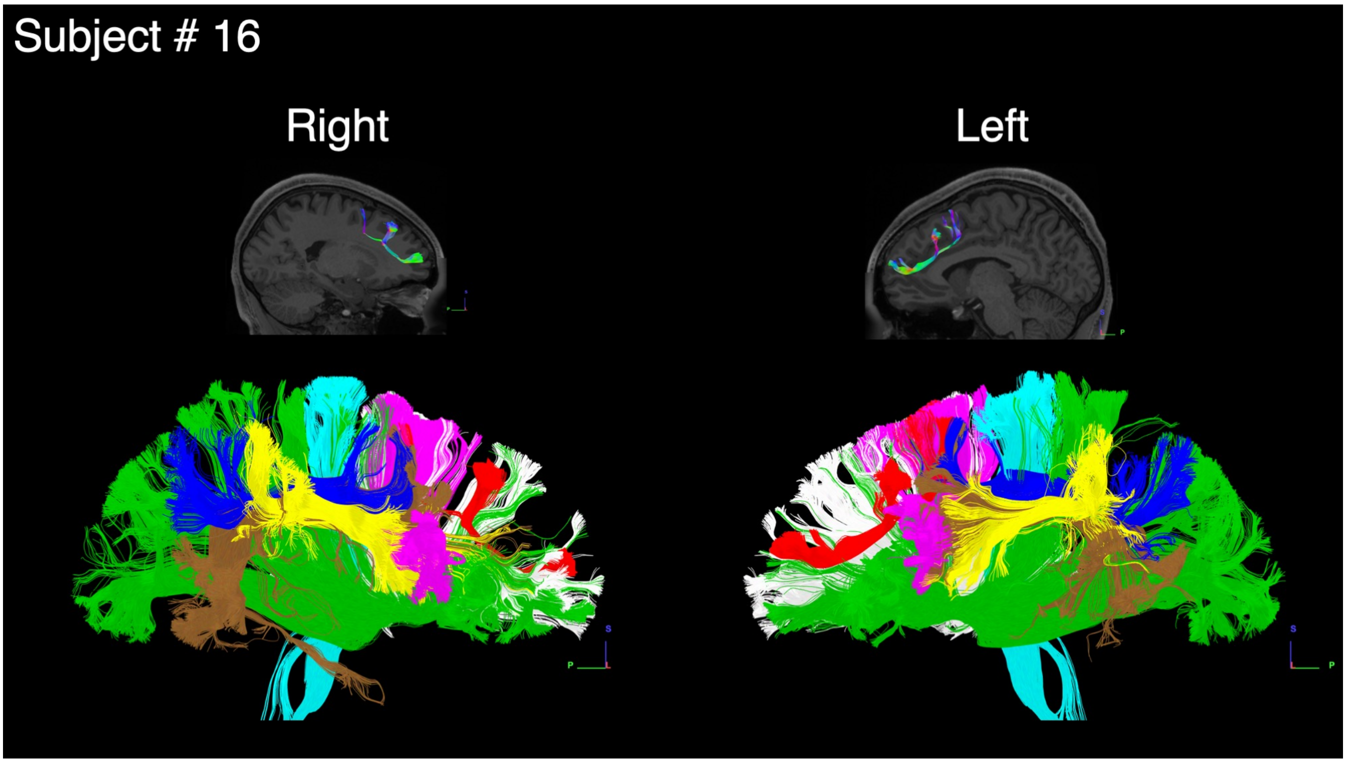
Subject # 16
Right
Left

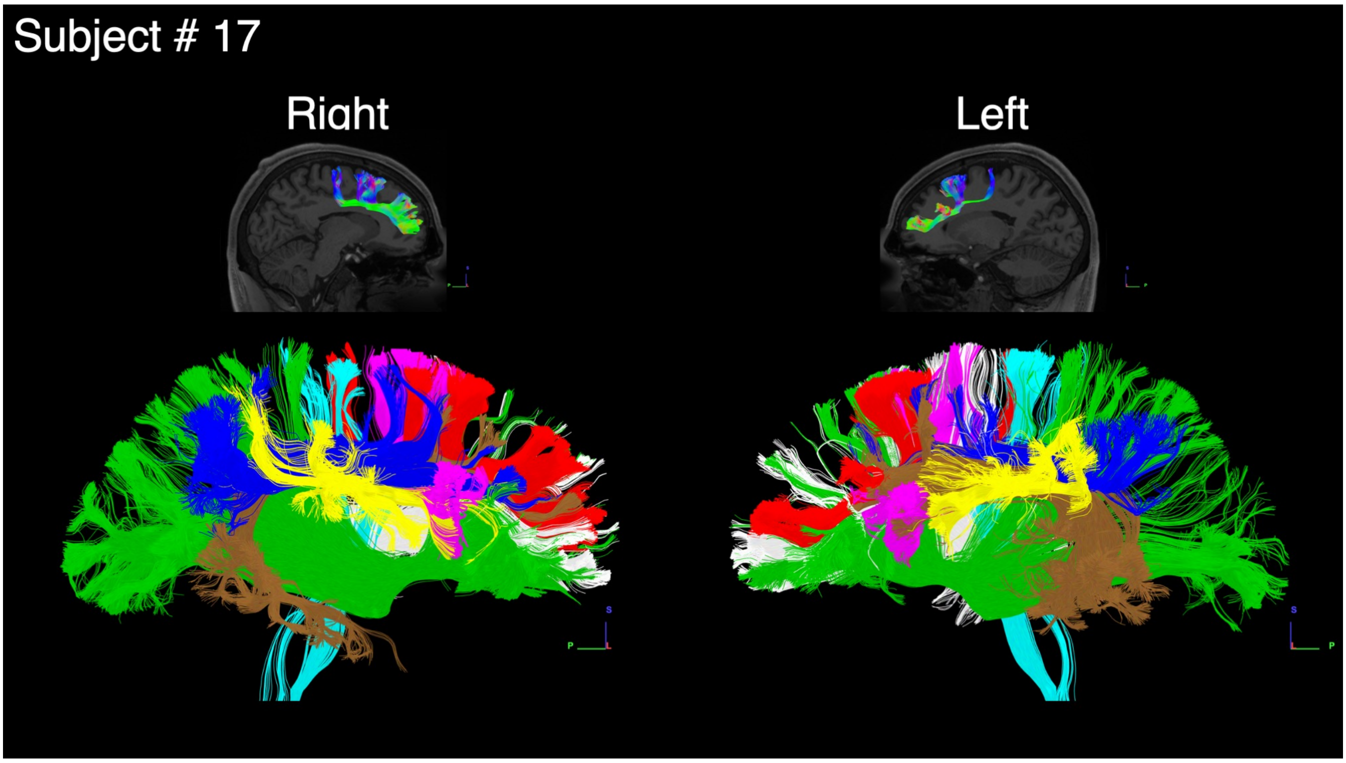
Subject # 17
Right
Left

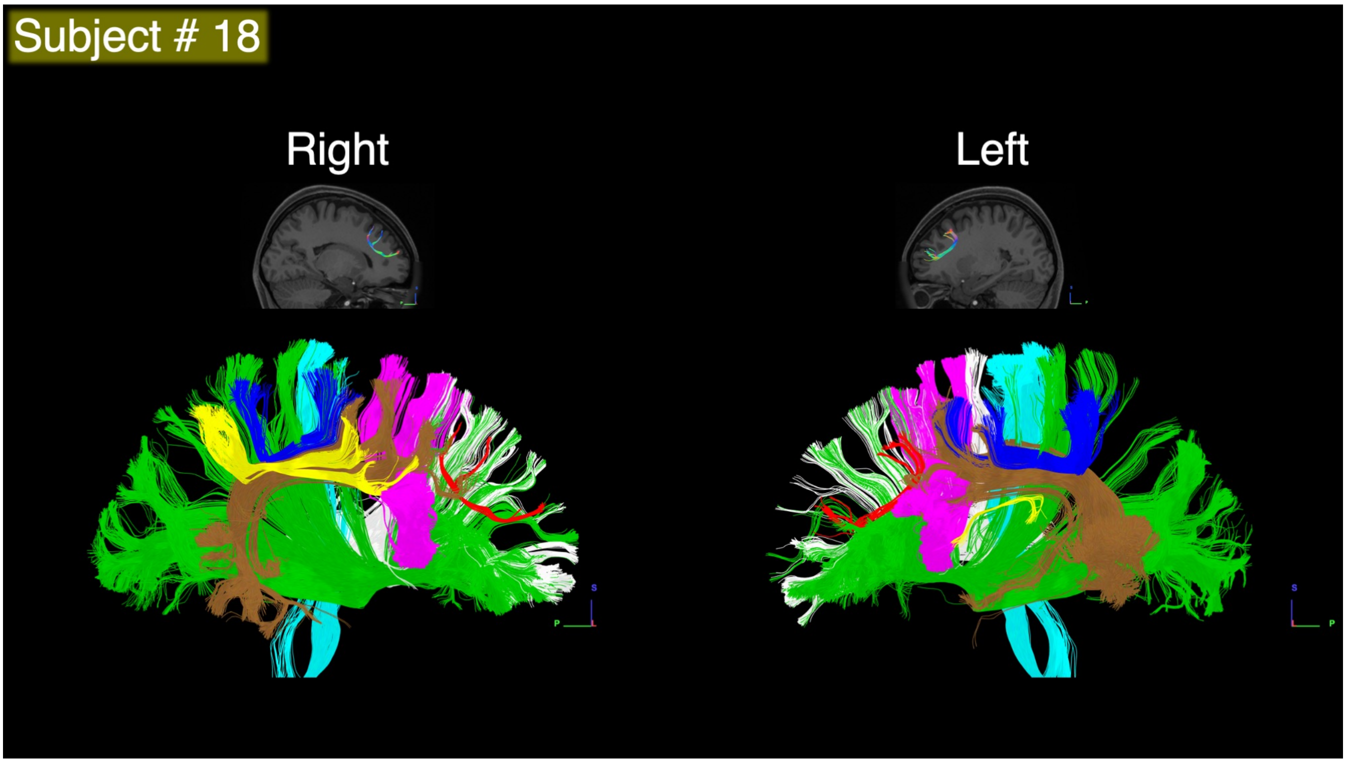
Subject # 18
Right
Left

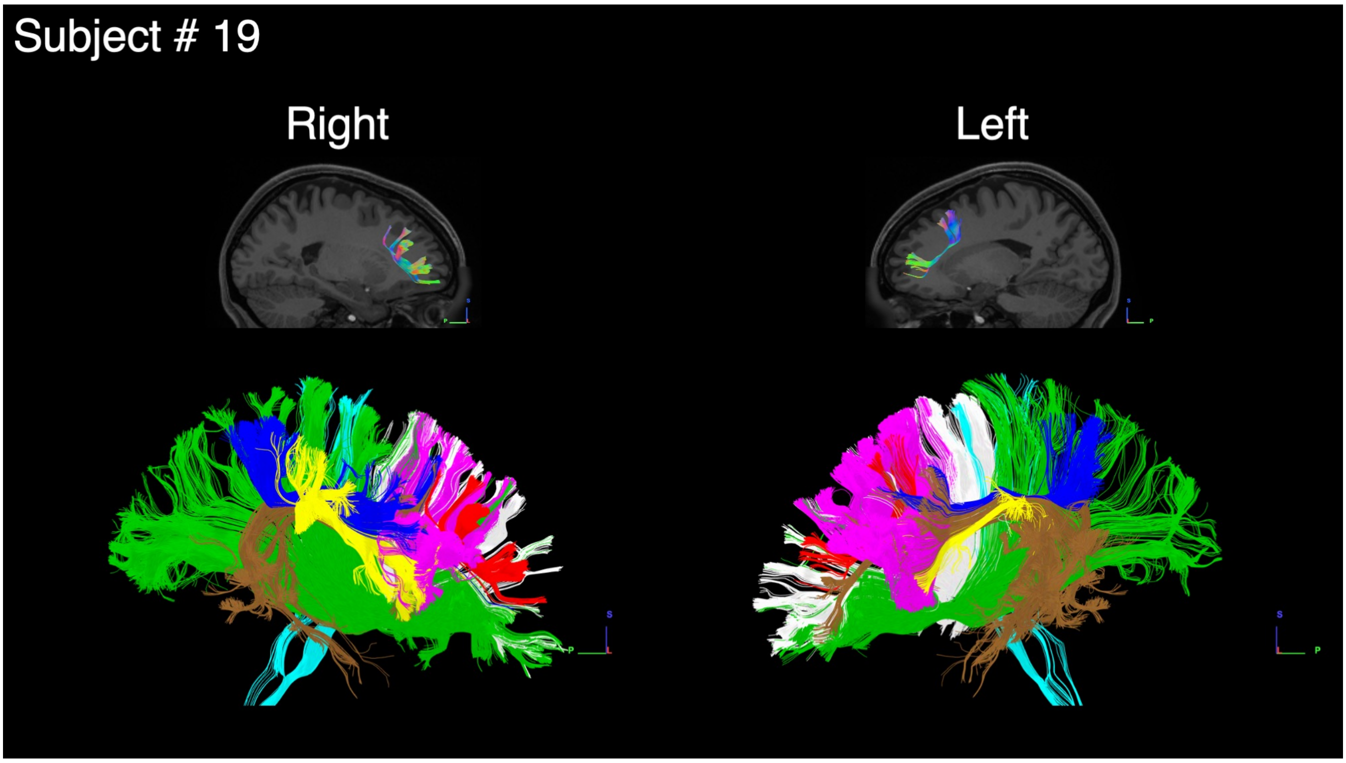
Subject # 19
Right
Left

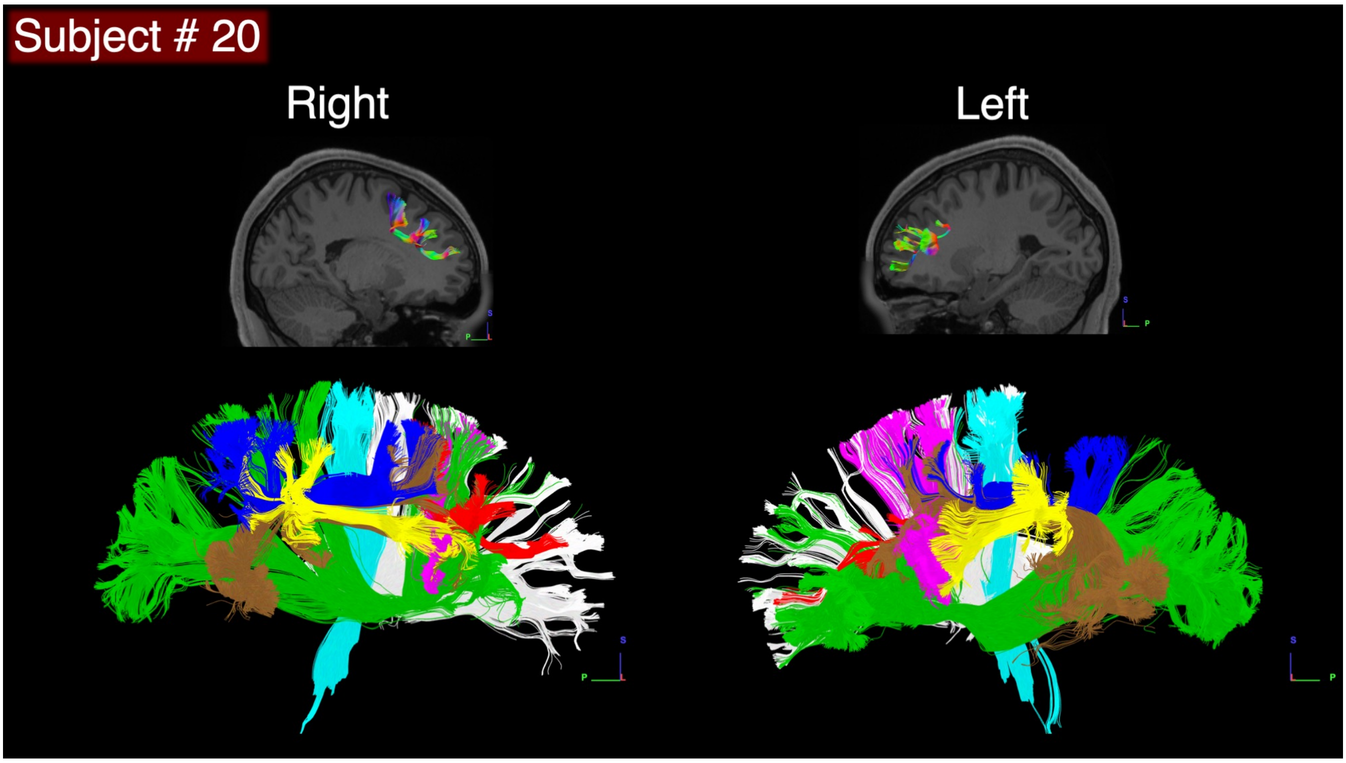
Subject # 20
Right
Left

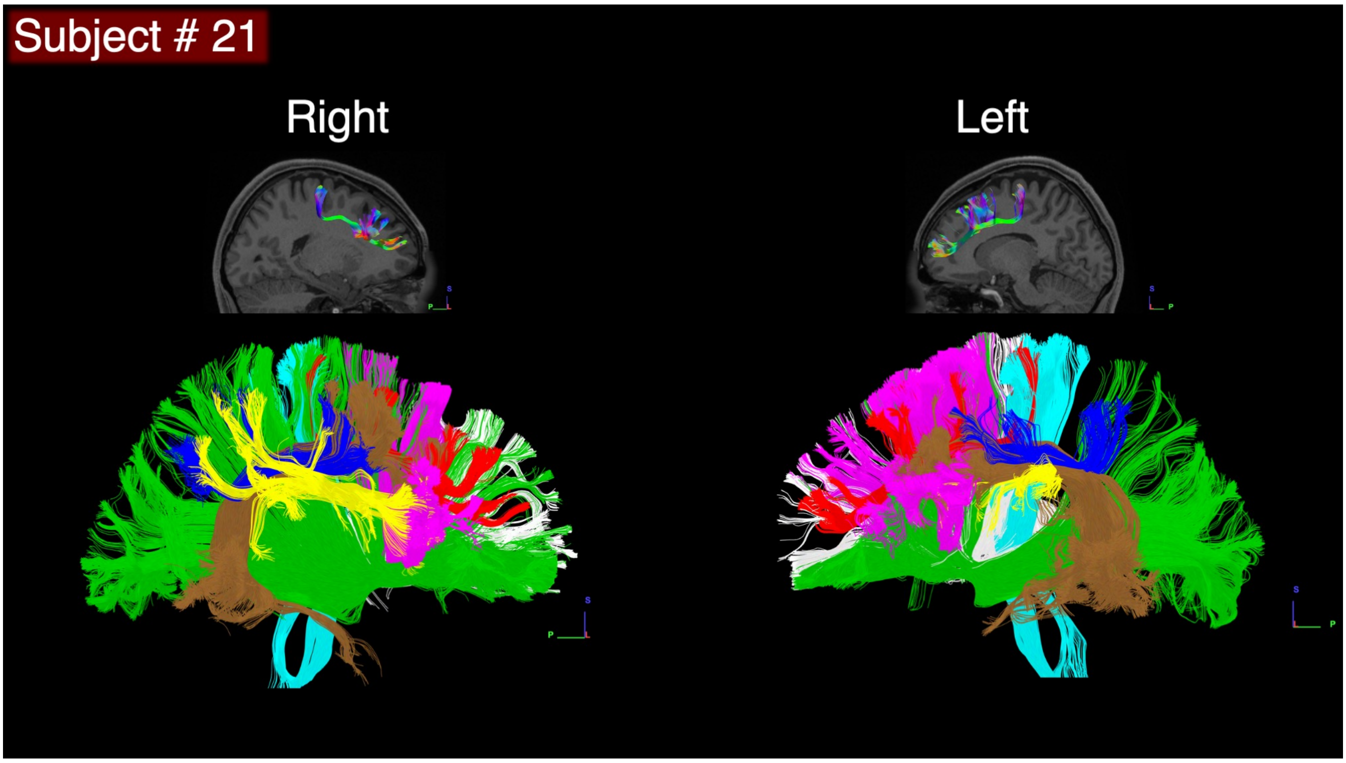
Subject # 21
Right
Left

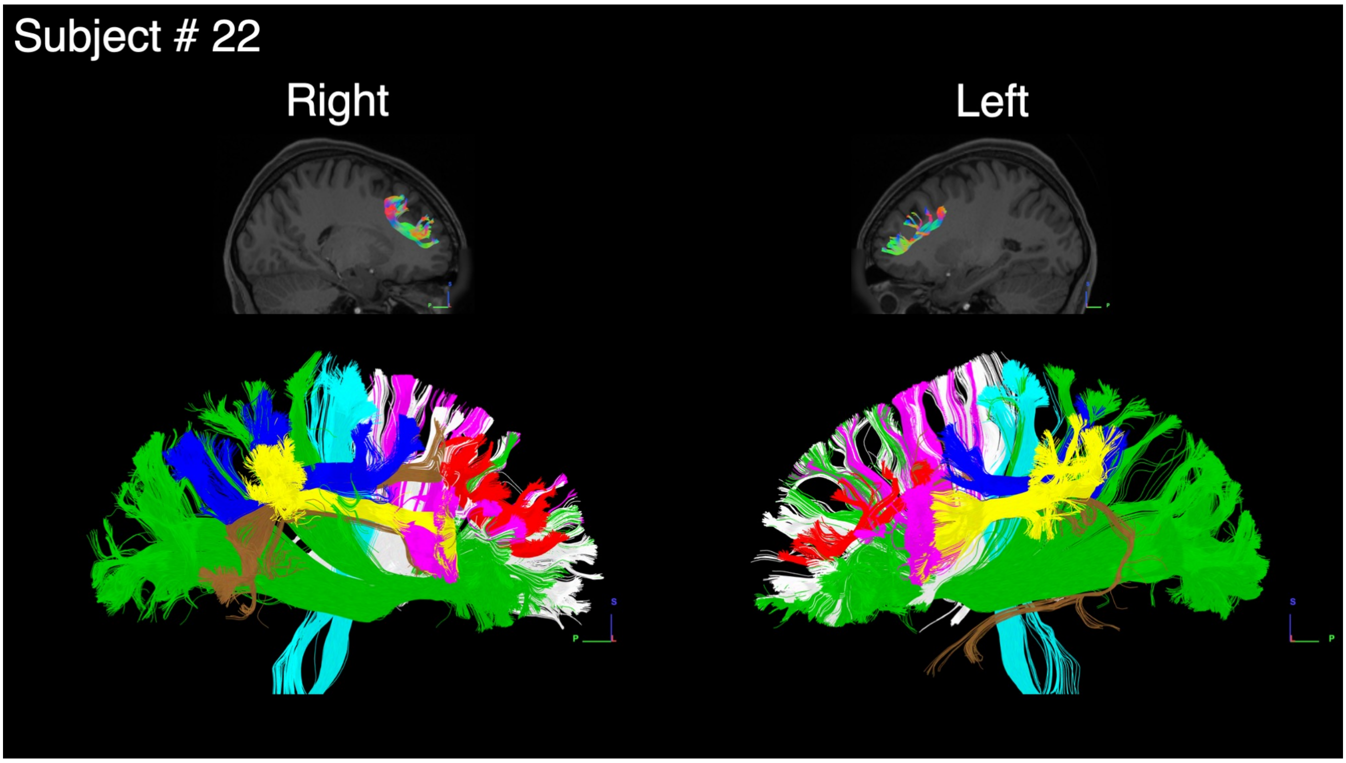
Subject # 22
Right
Left

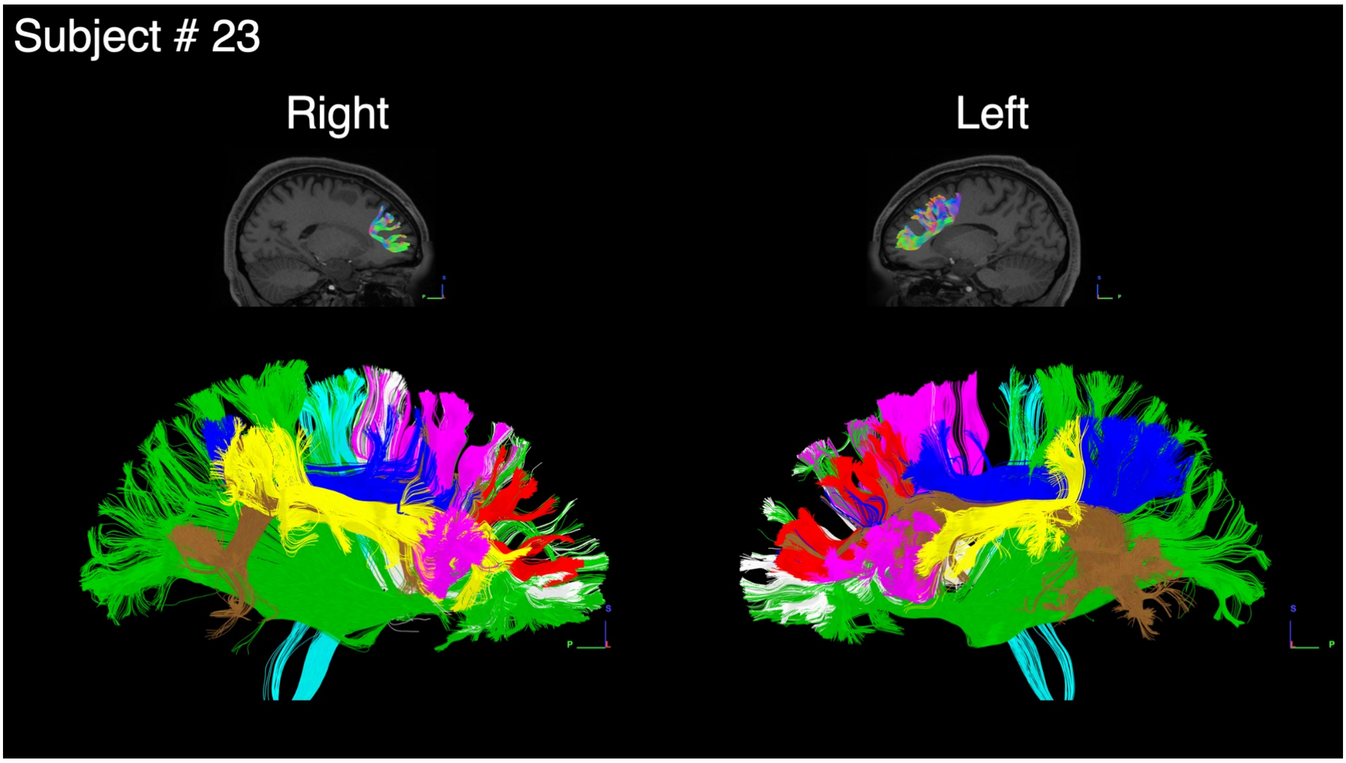
Subject # 23
Right
Left

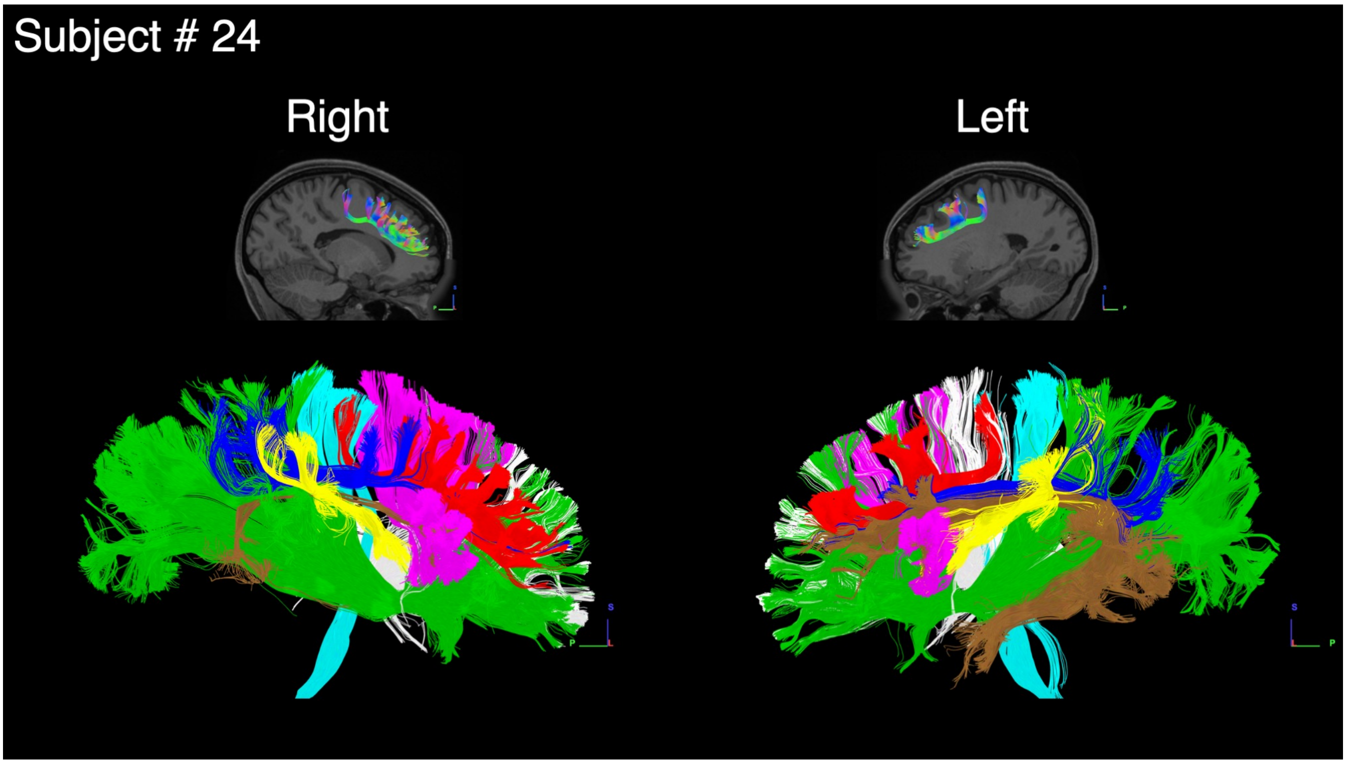
Subject # 24
Right
Left

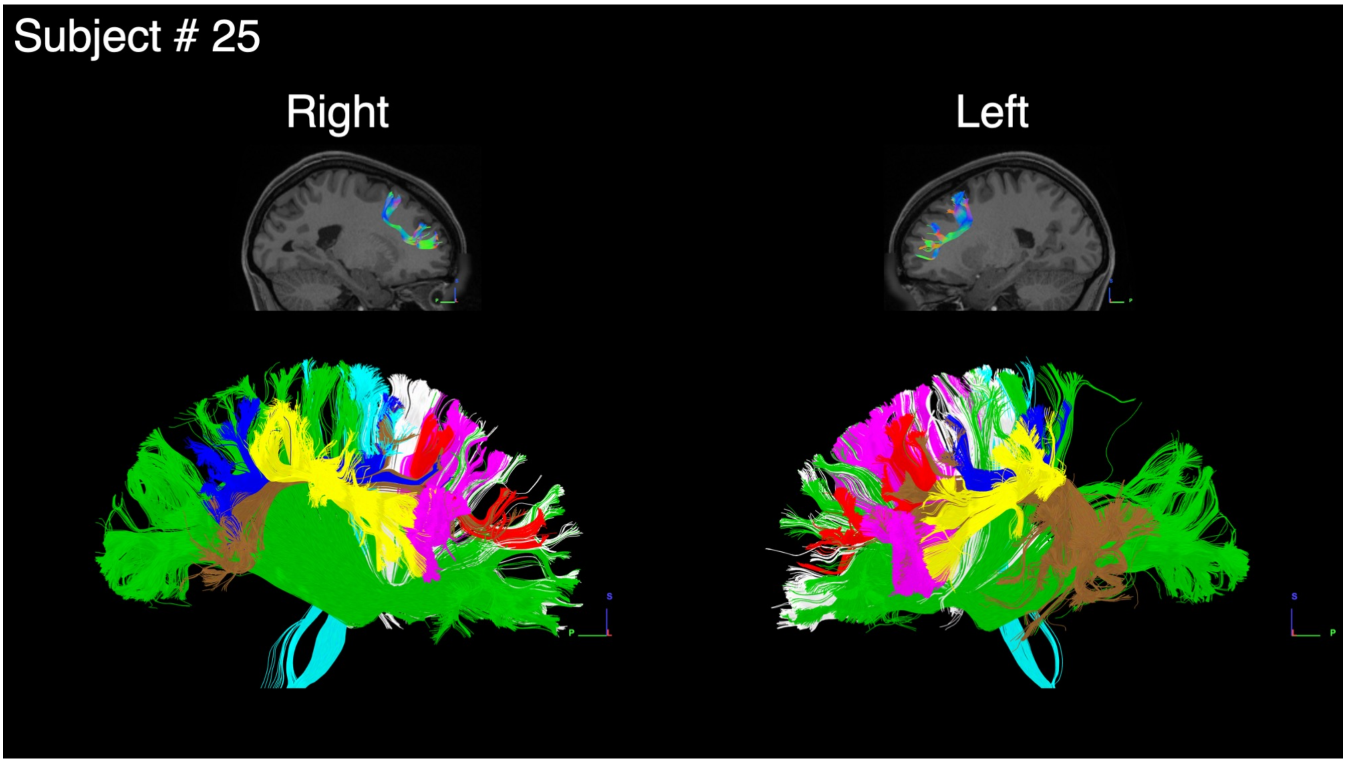
Subject # 25
Right
Left

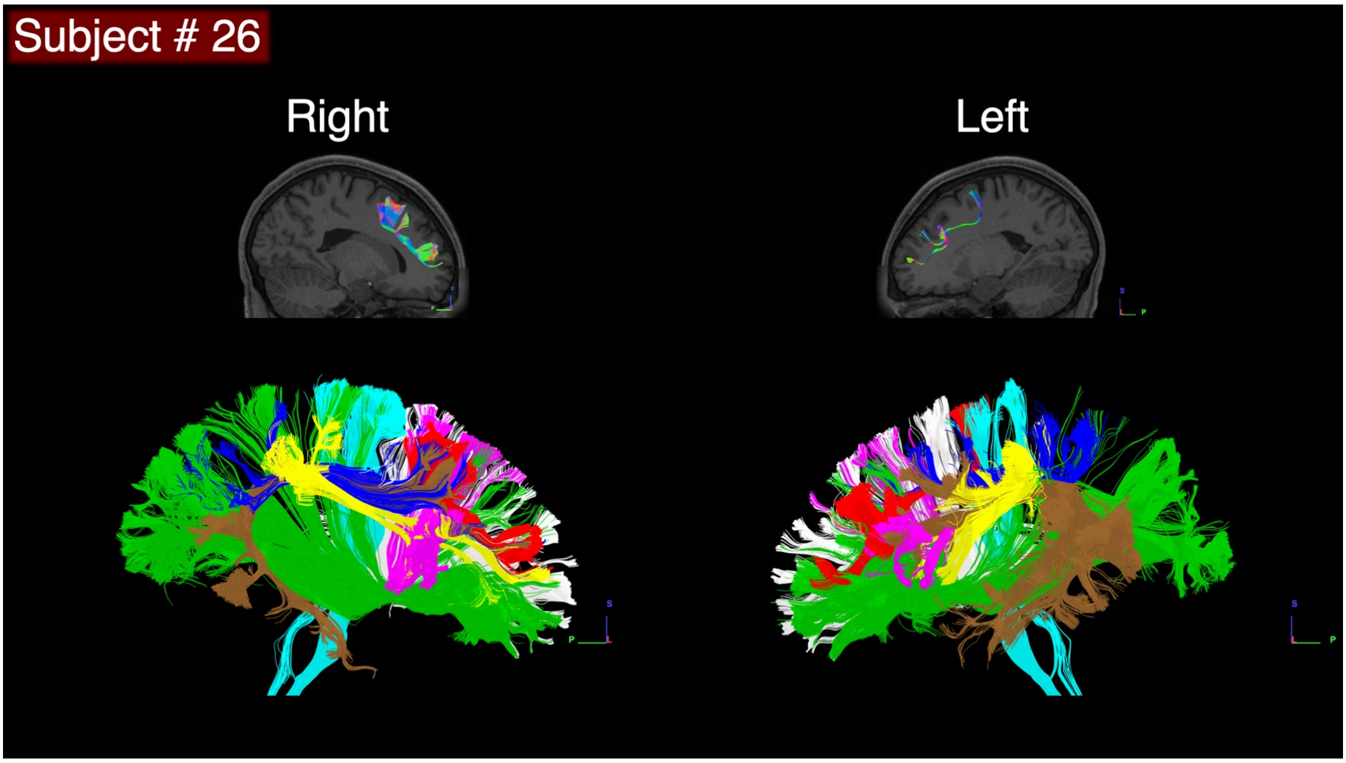
Subject # 26
Right
Left

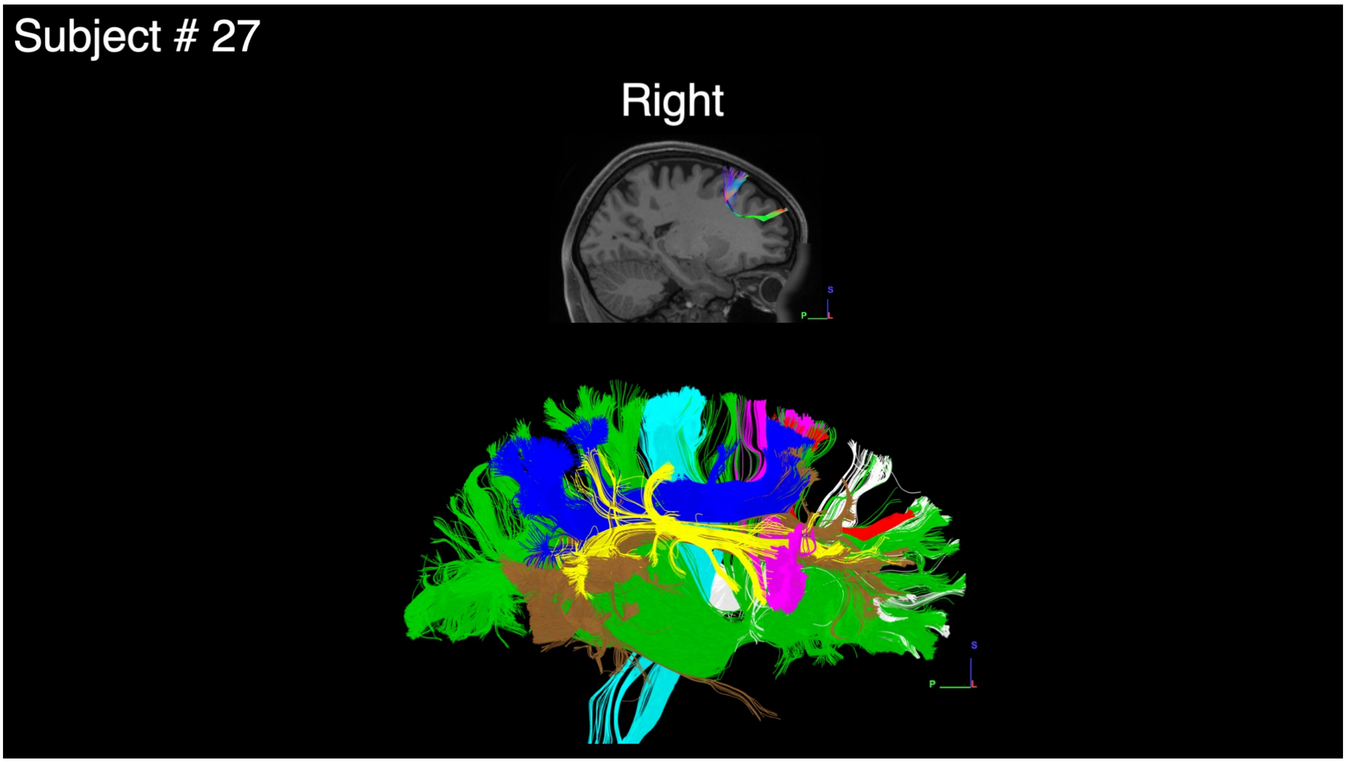
Subject # 27
Right

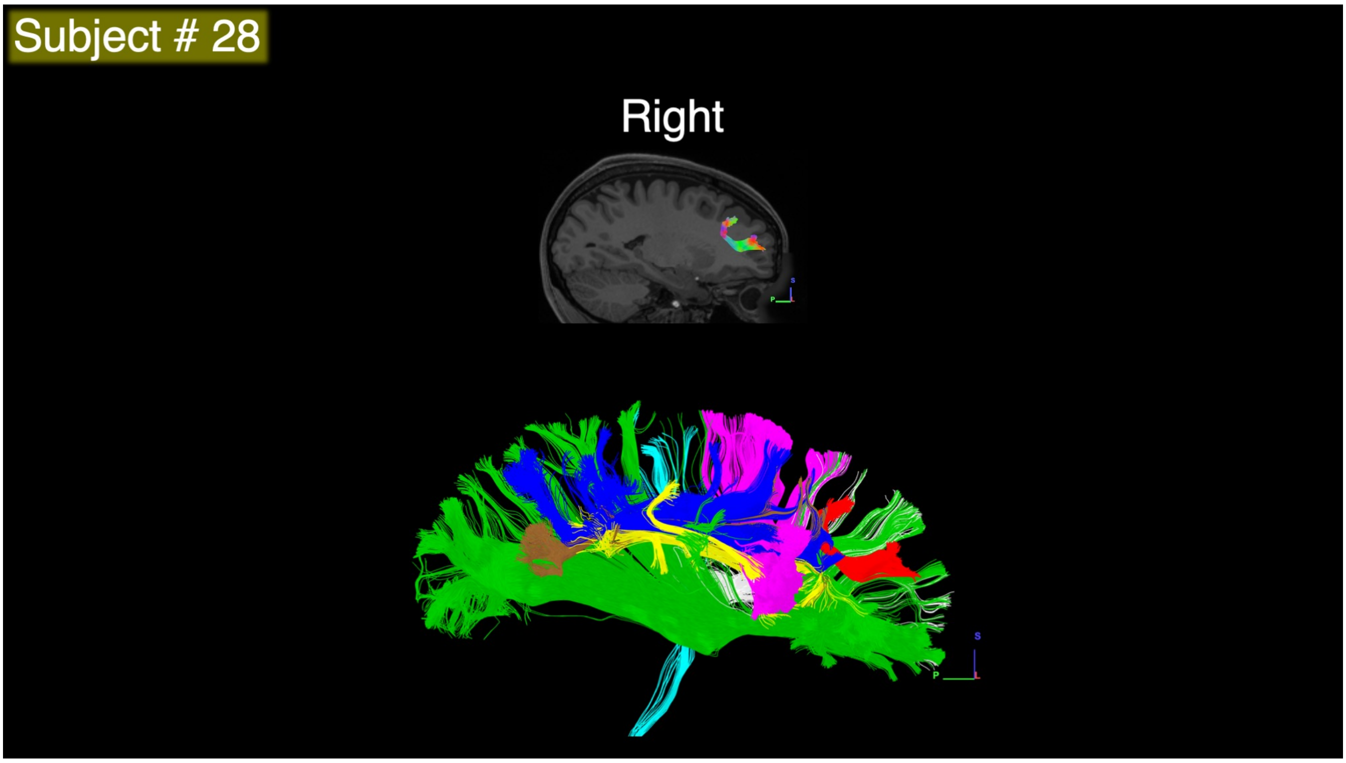
Subject # 28
Right

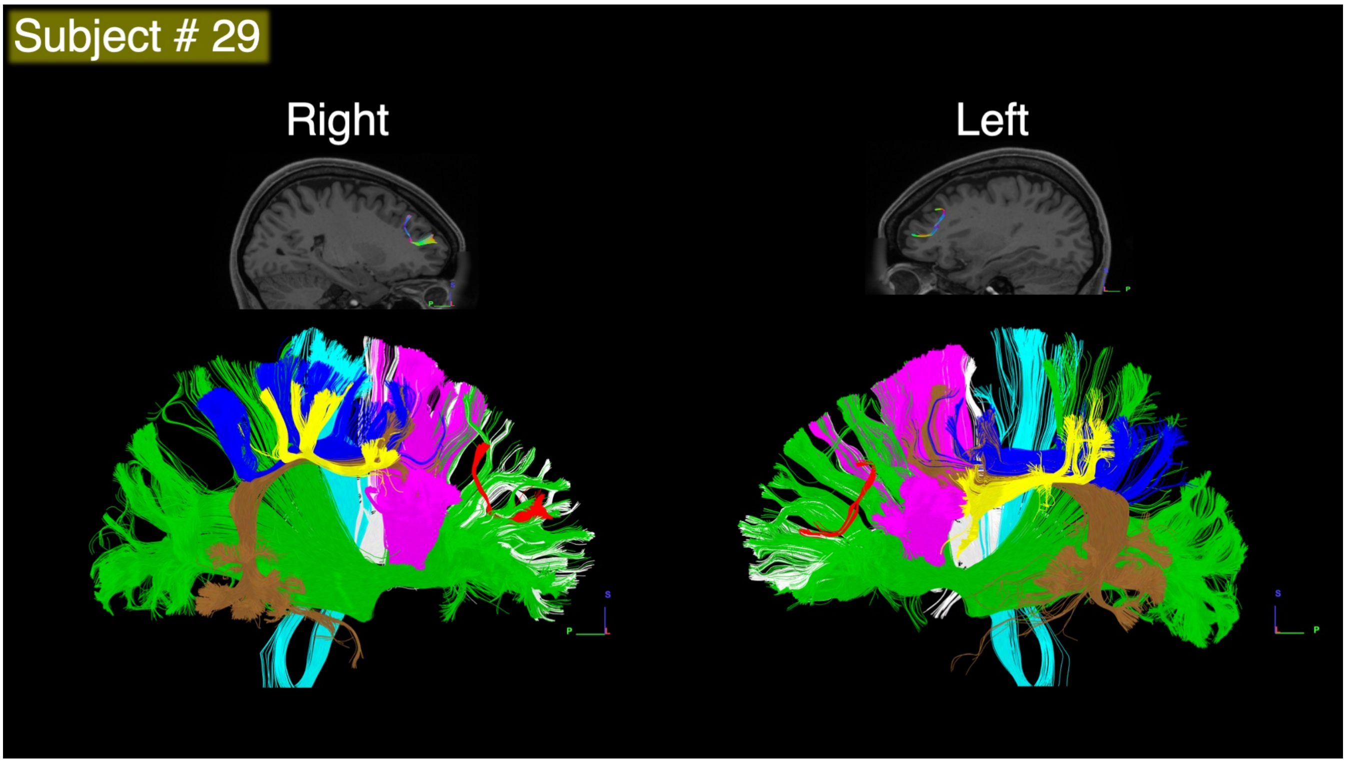
Subject # 29
Right
Left

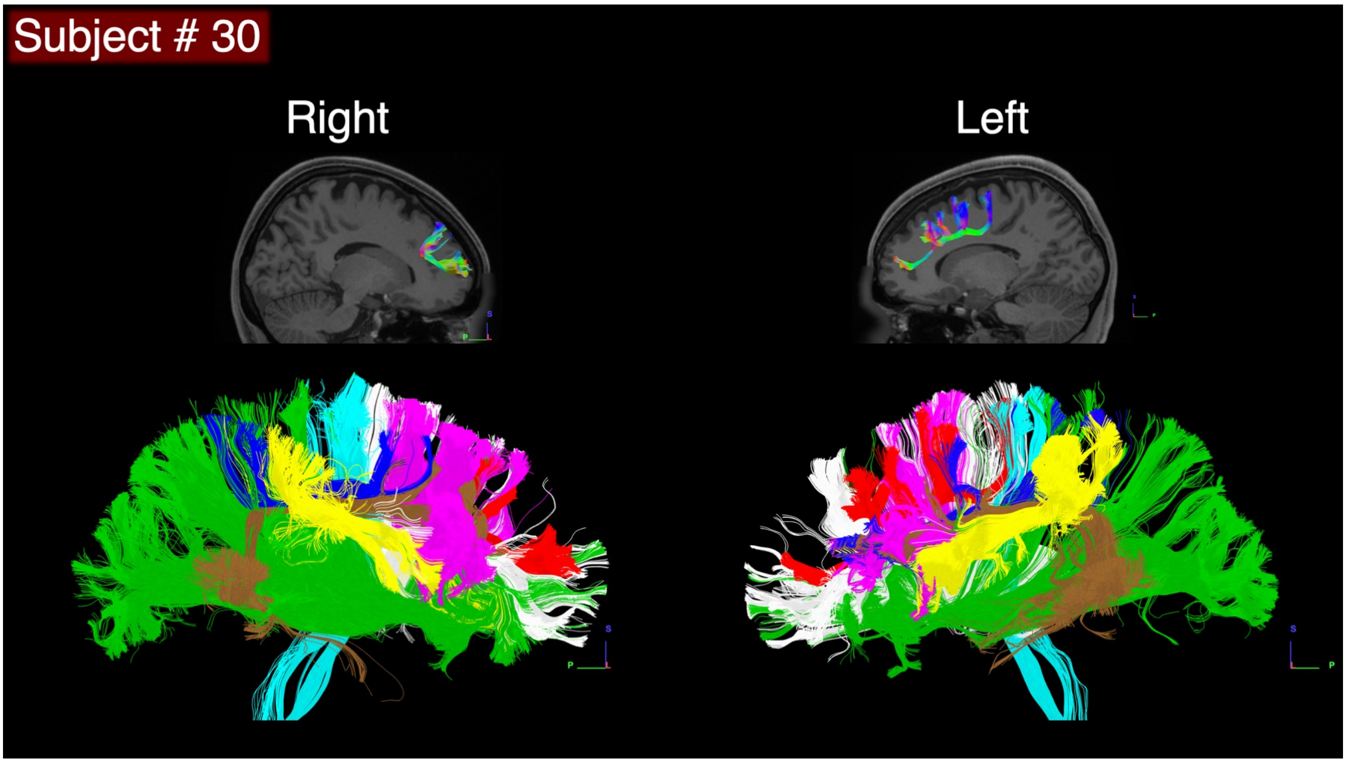
Subject # 30
Right
Left

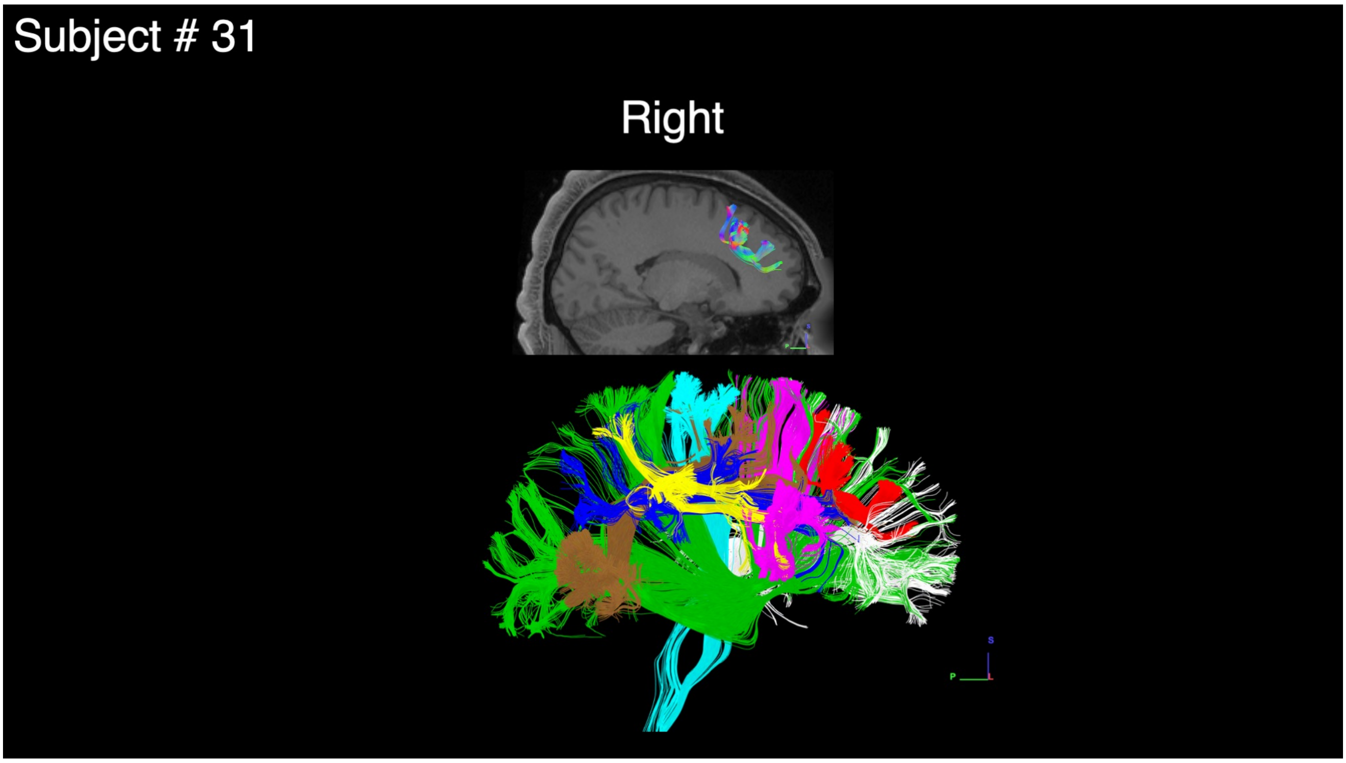
Subject # 31
Right

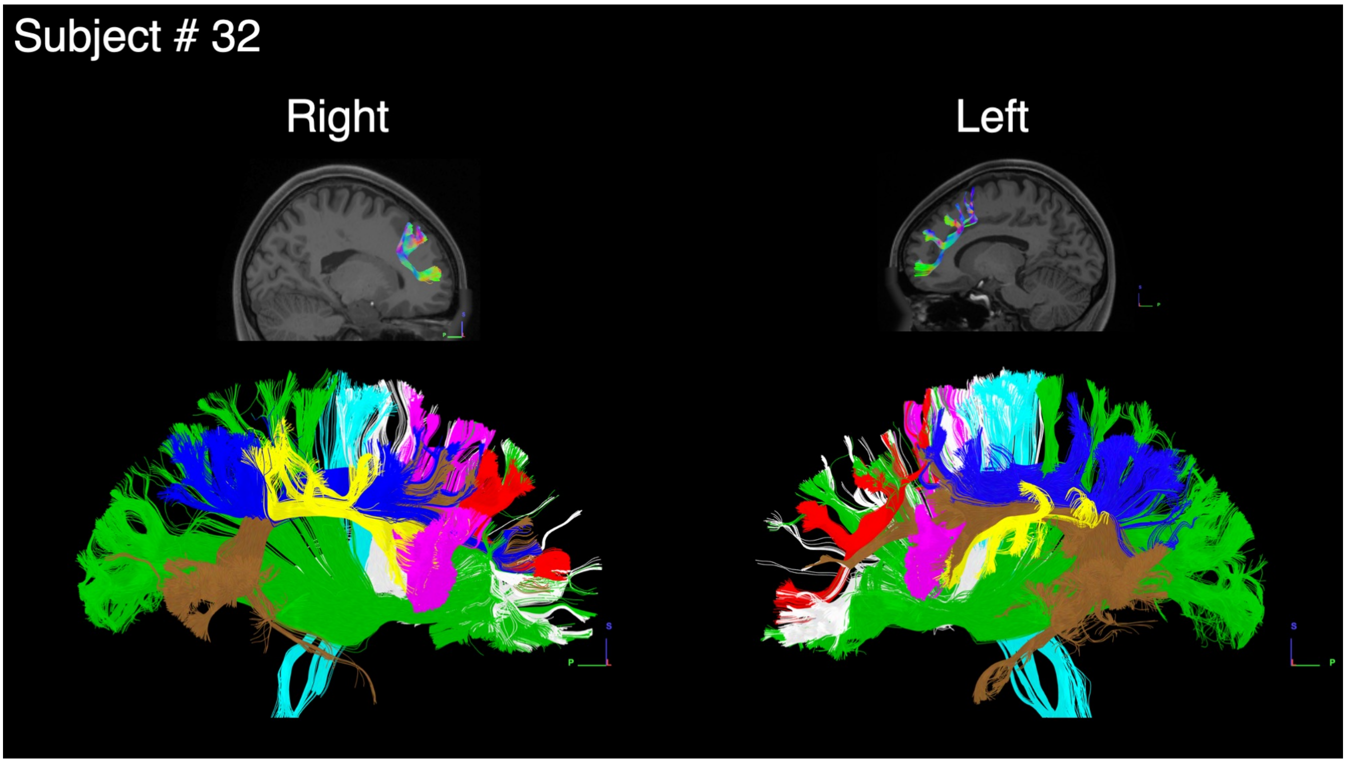
Subject # 32
Right
Left

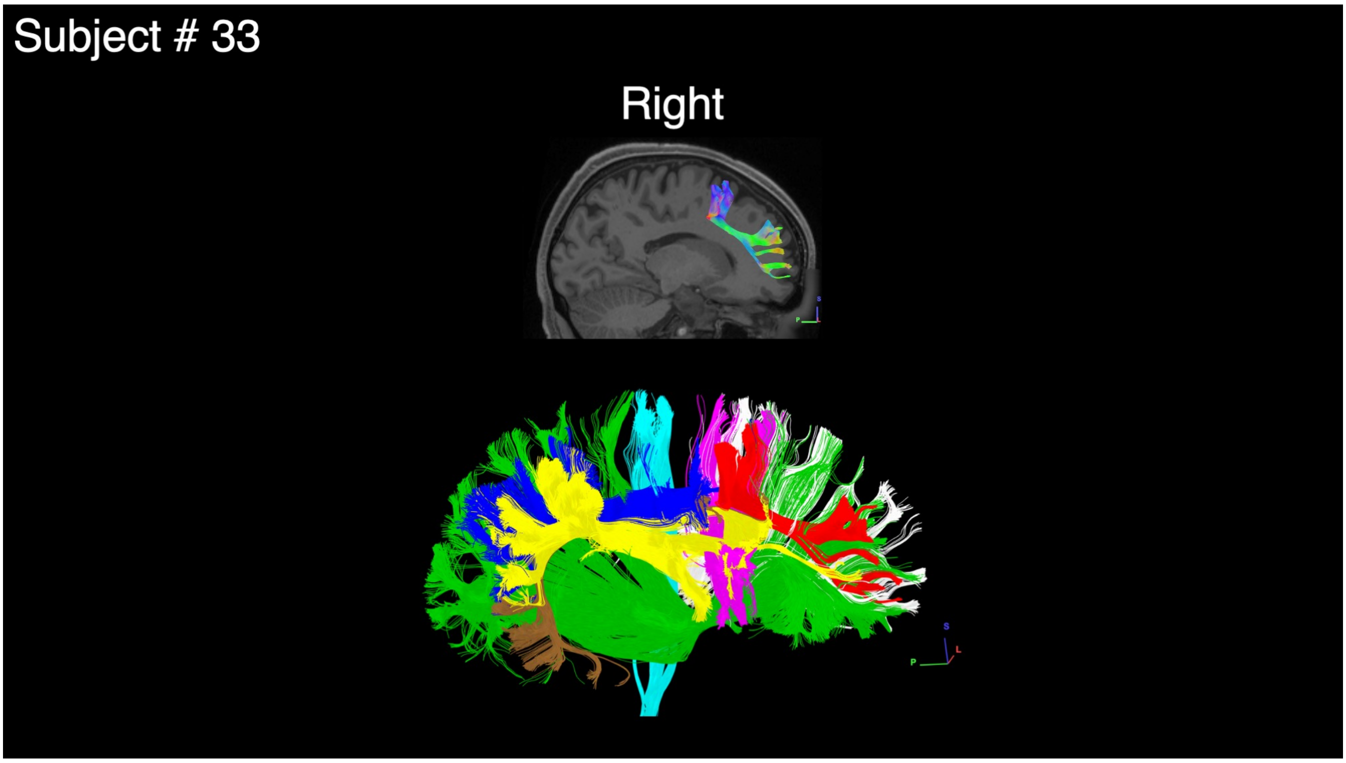
Subject # 33
Right

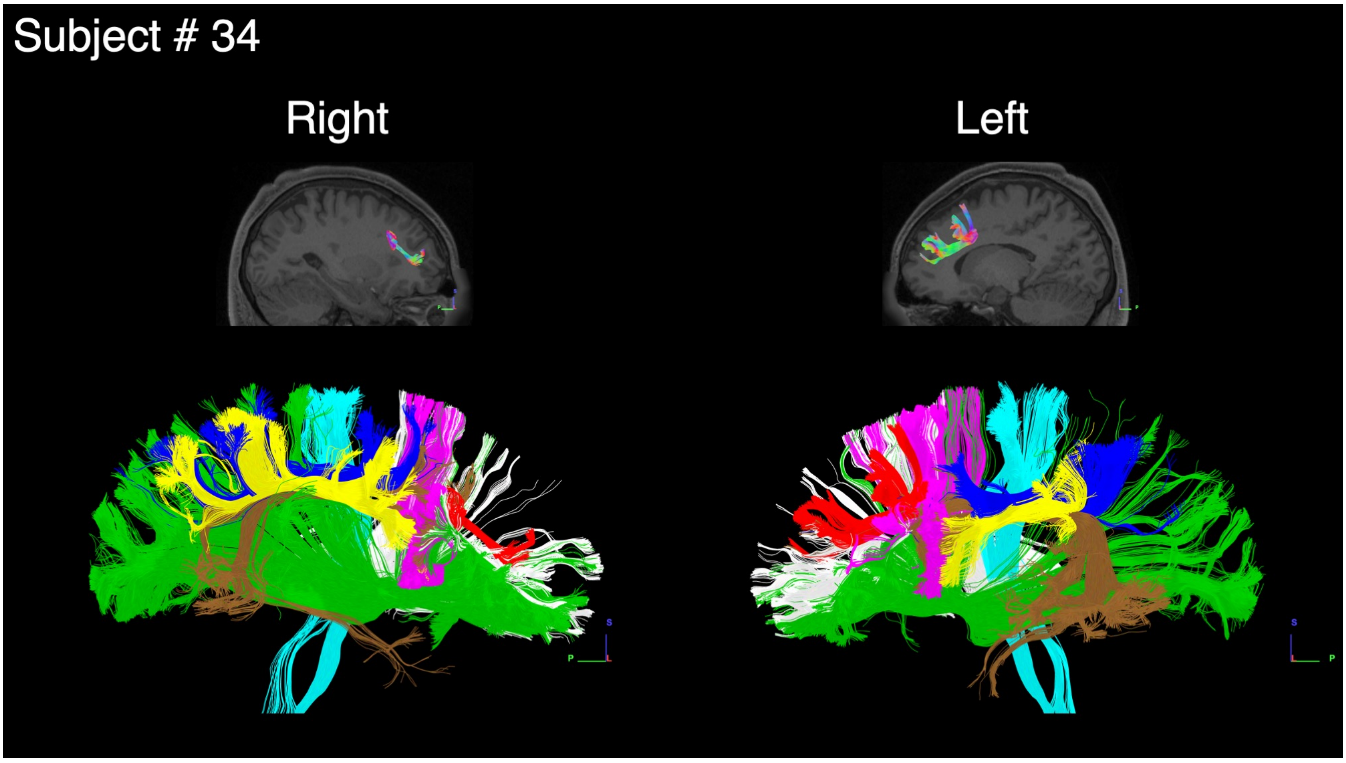
Subject # 34
Right
Left

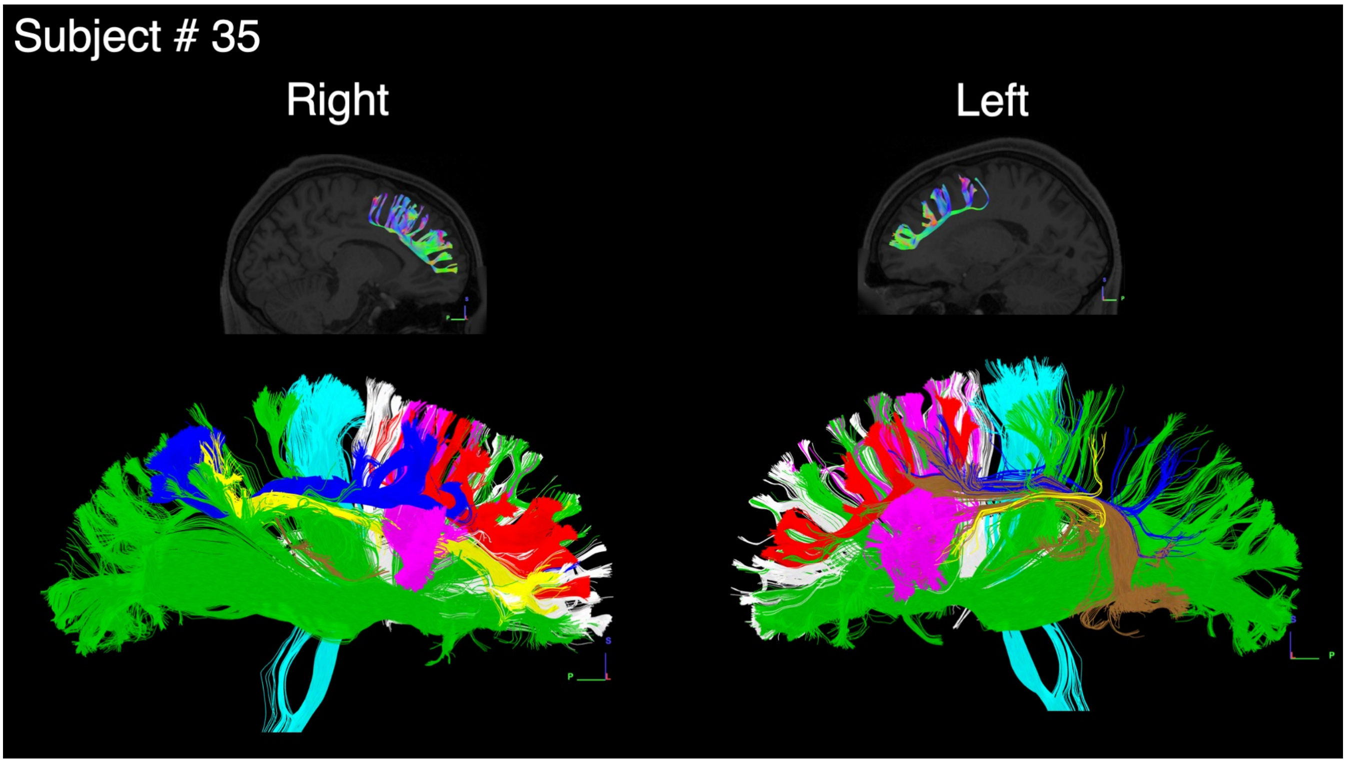
Subject # 35
Right
Left

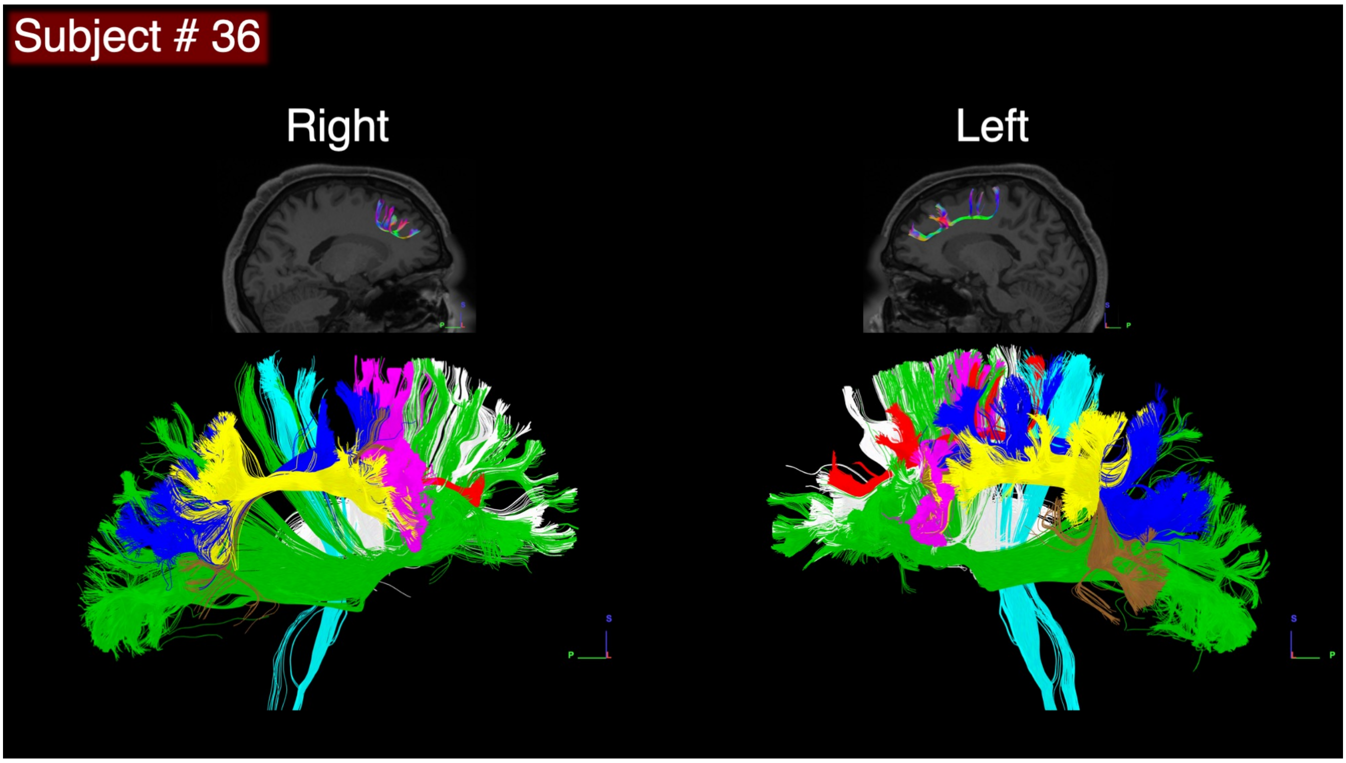
Subject # 36
Right
Left

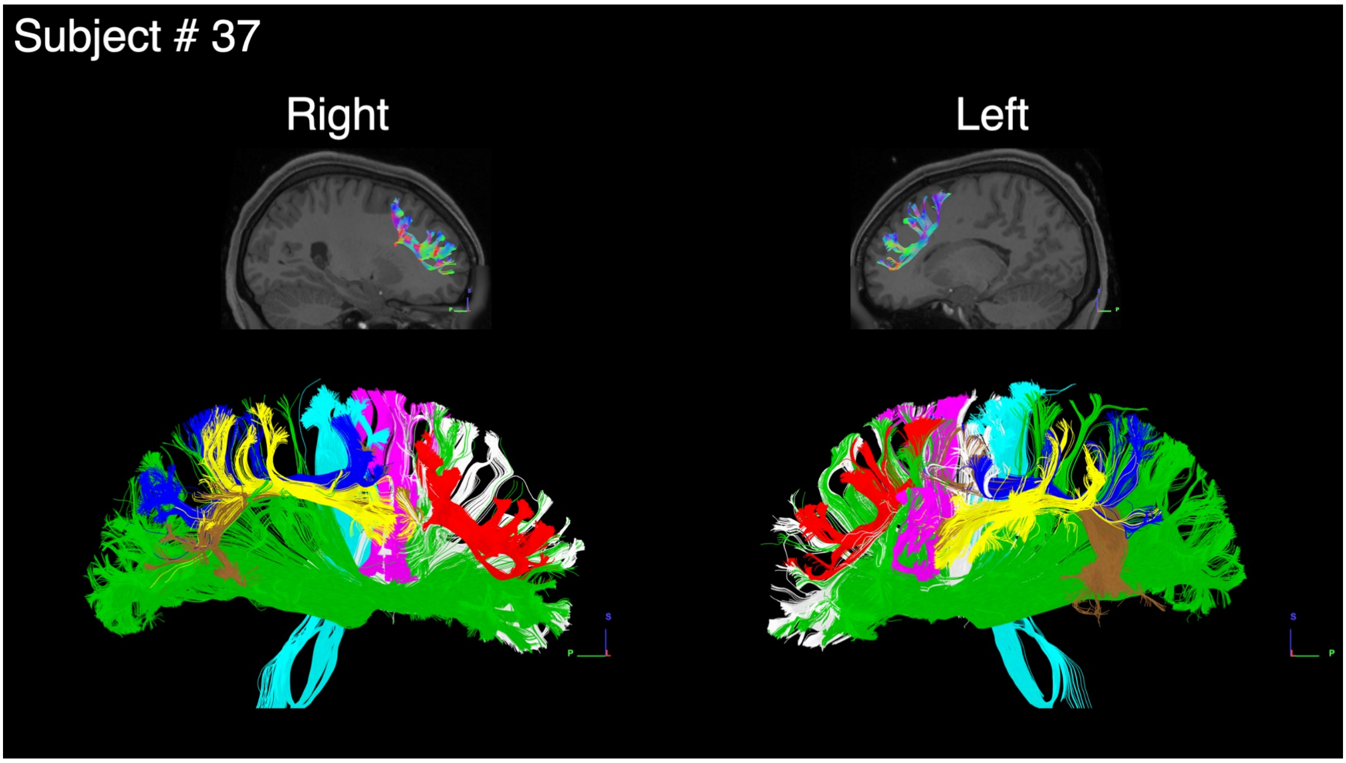

Subject # 37
Right
Left

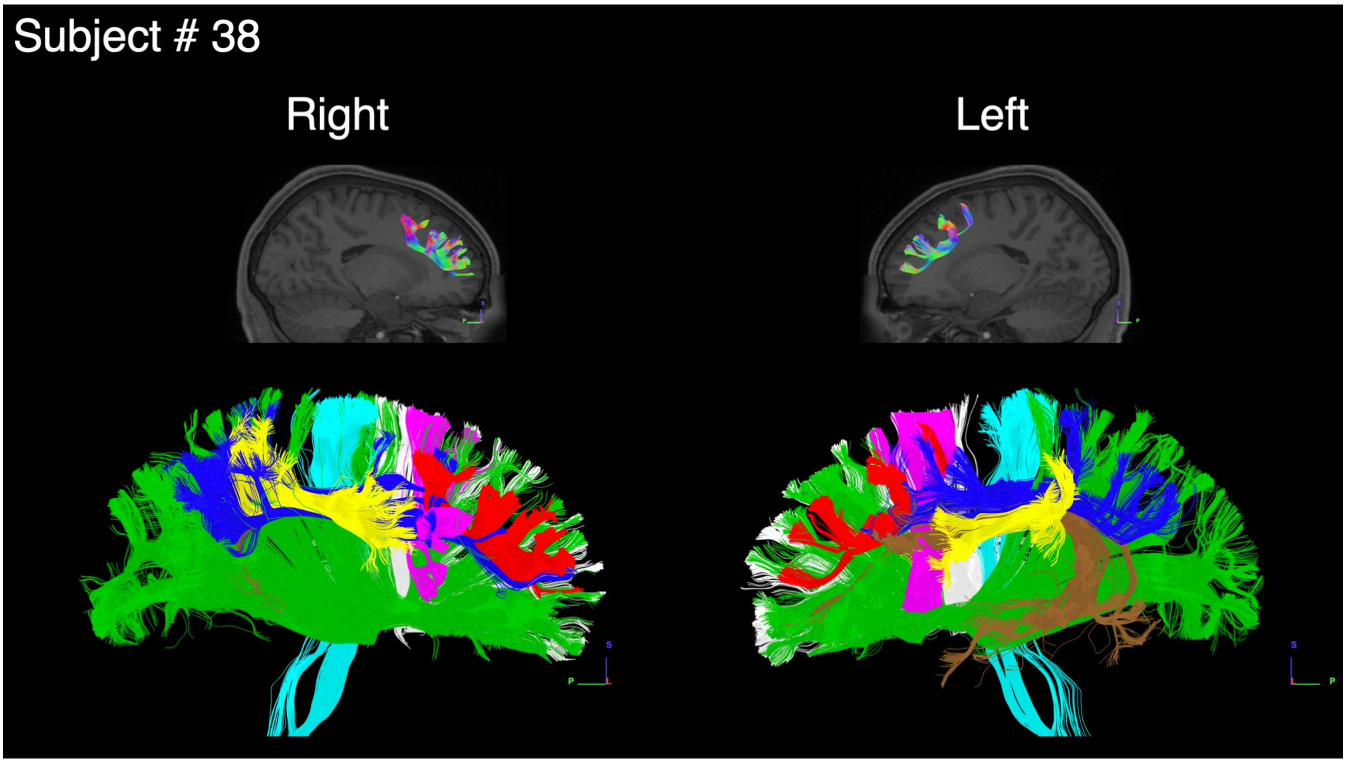

Subject # 38
Right
Left

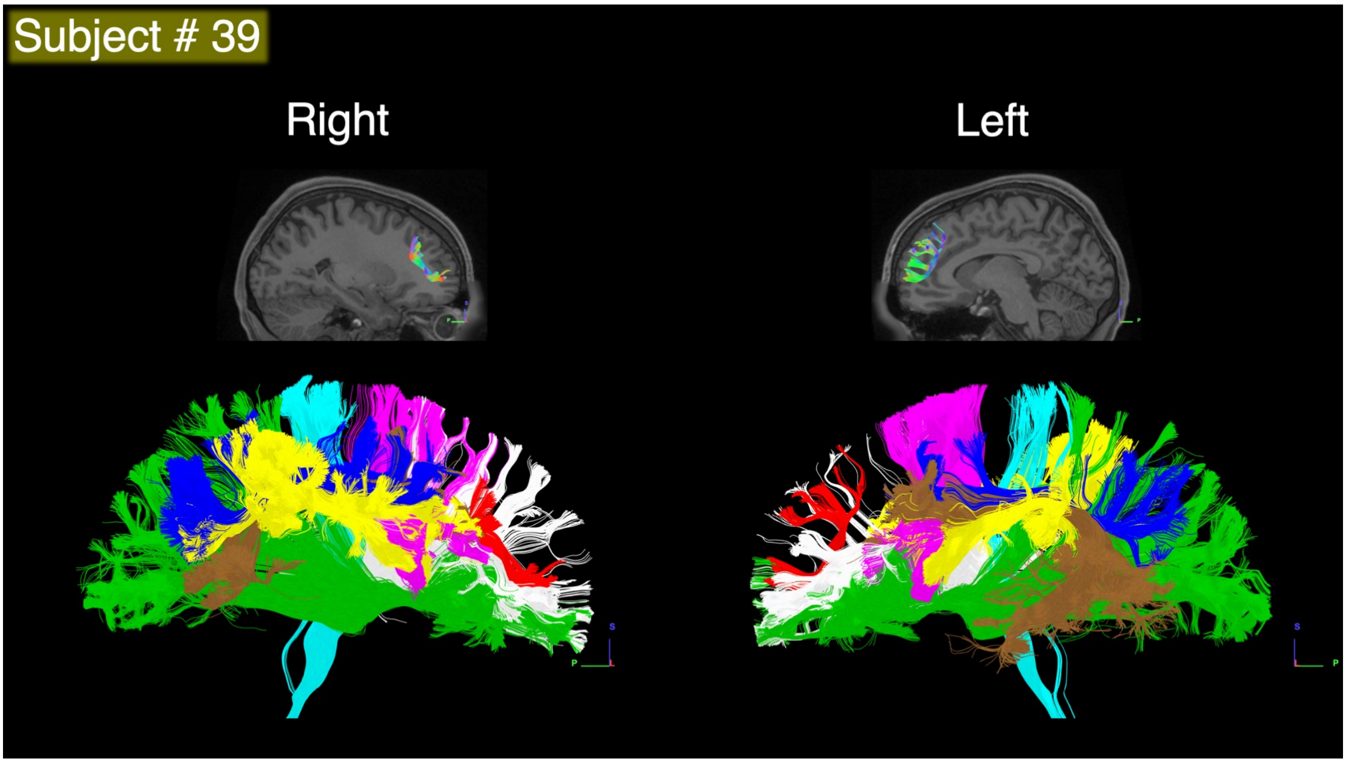
Subject # 39
Right
Left

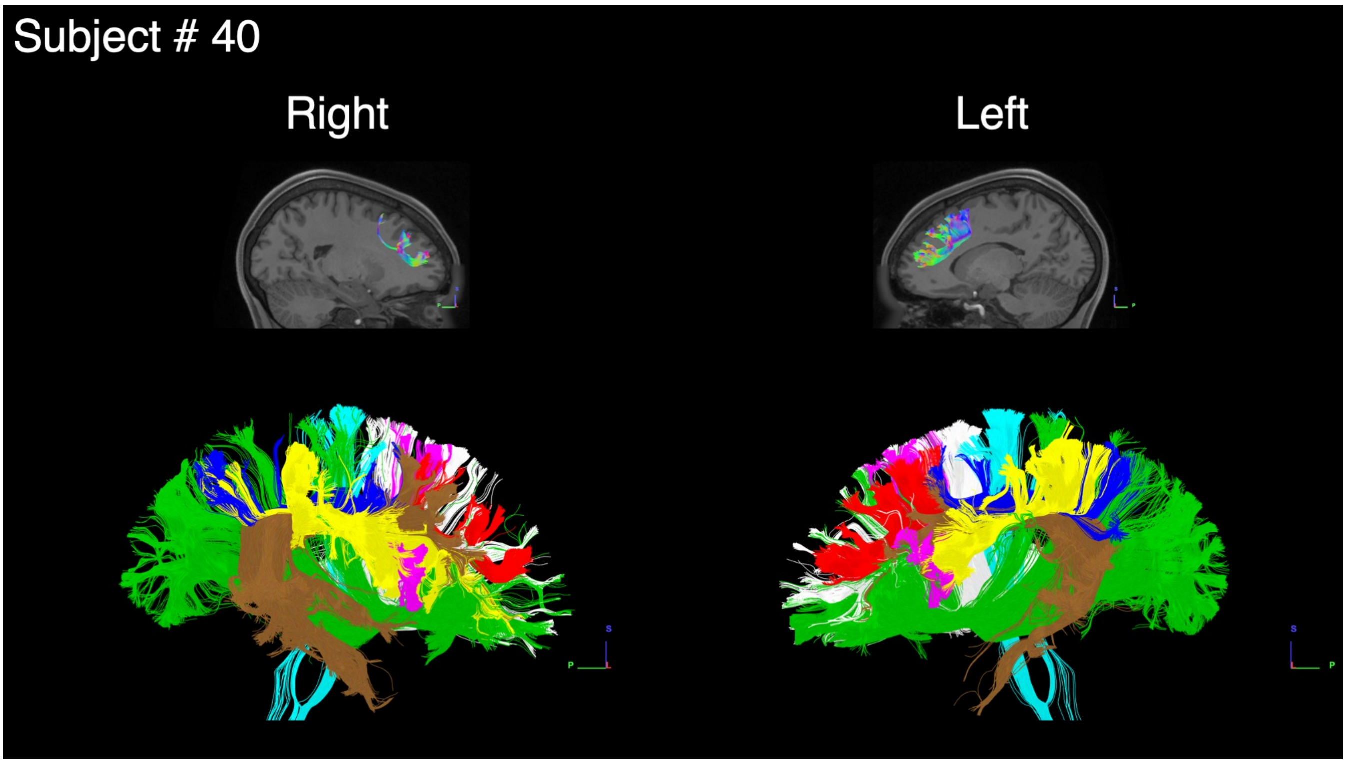
Subject # 40
Right
Left

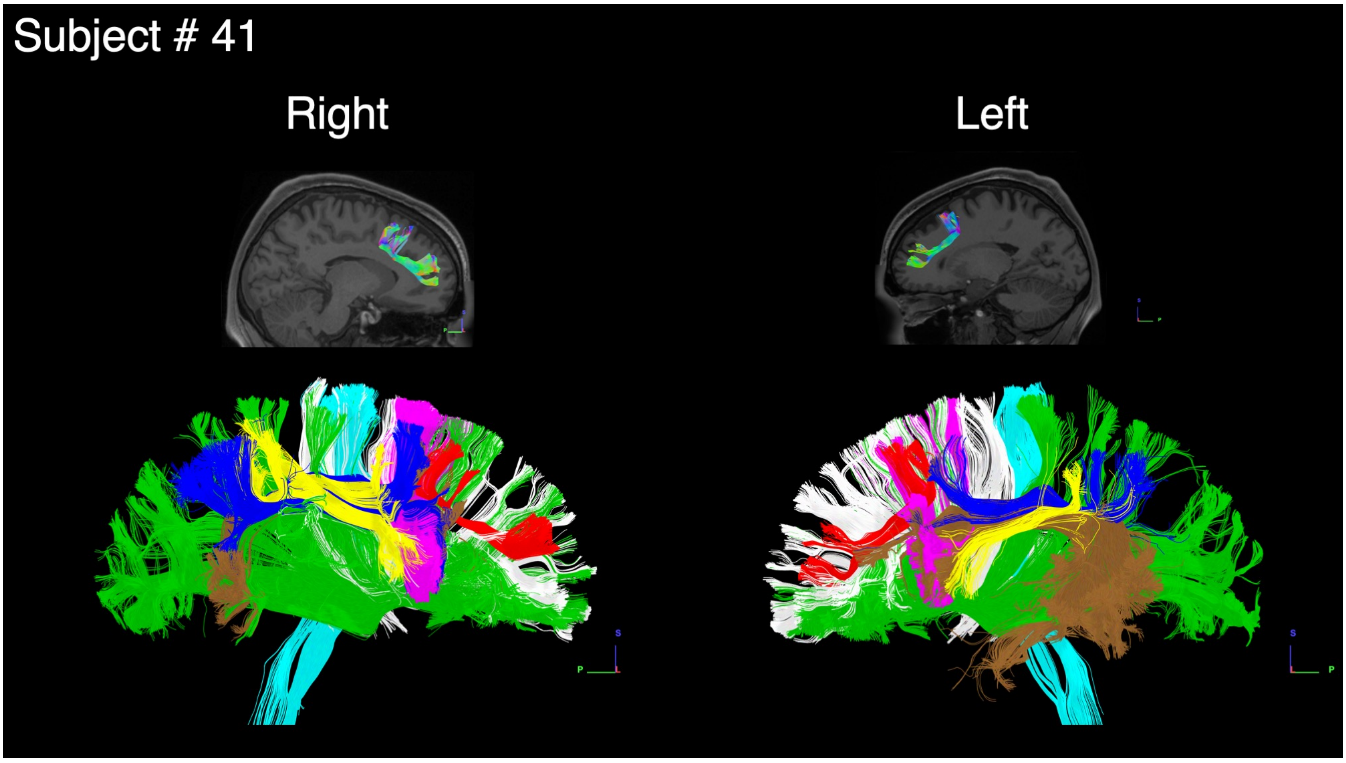
Subject # 41
Right
Left

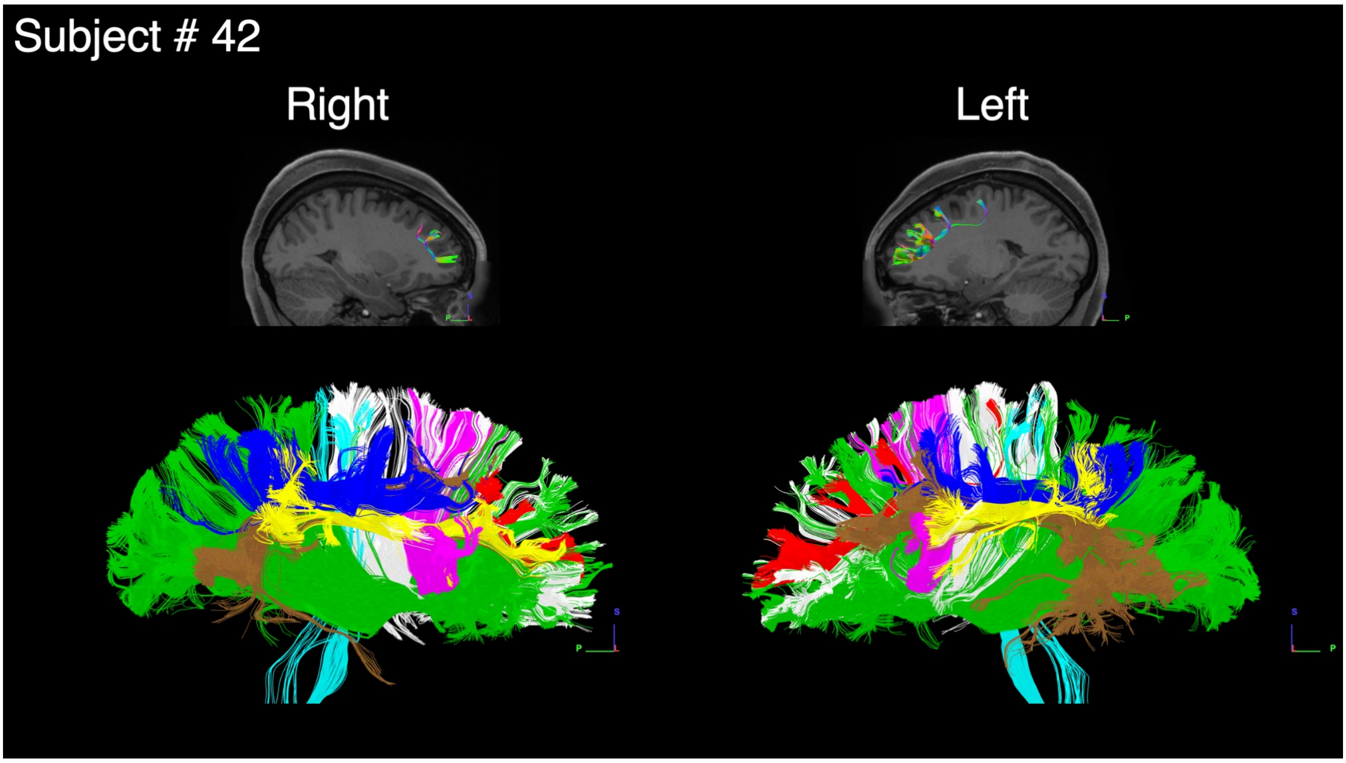
Subject # 42
Right
Left

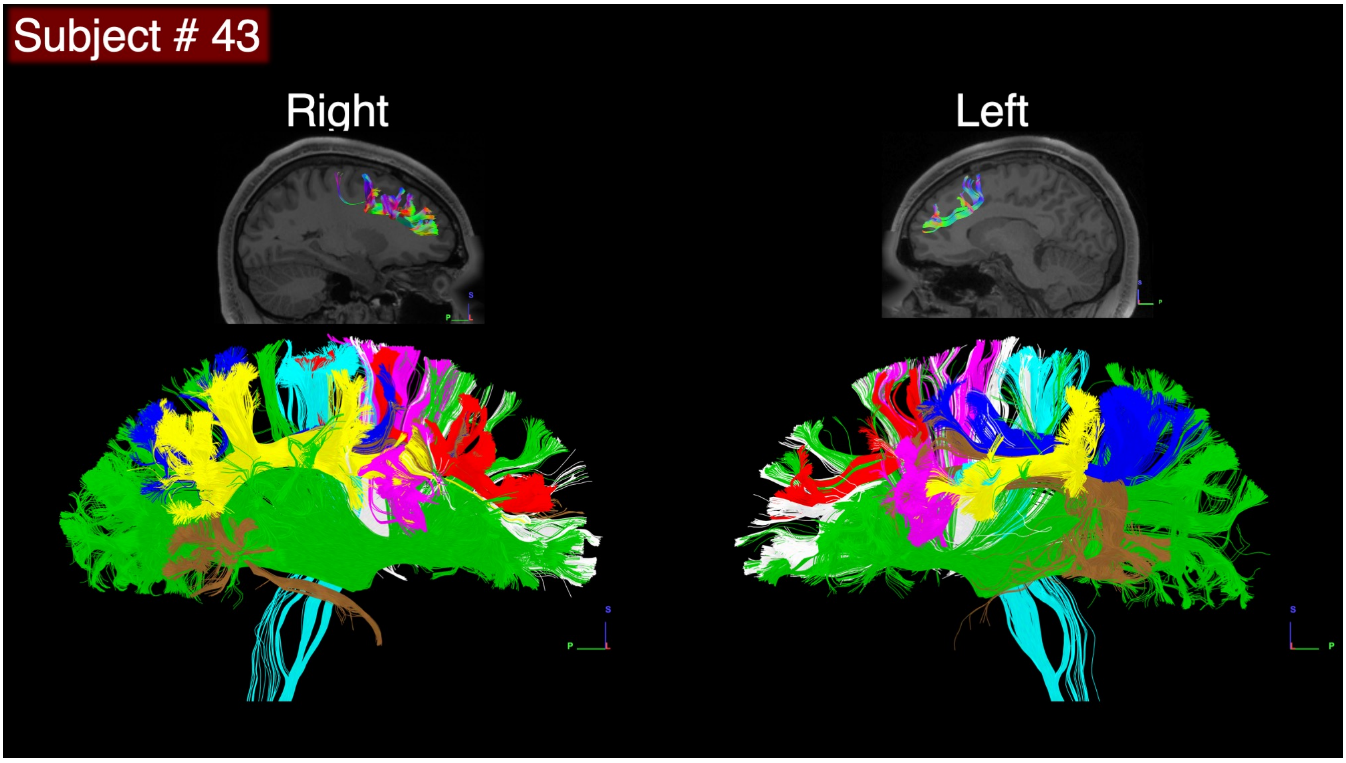
Subject # 43
Right
Left

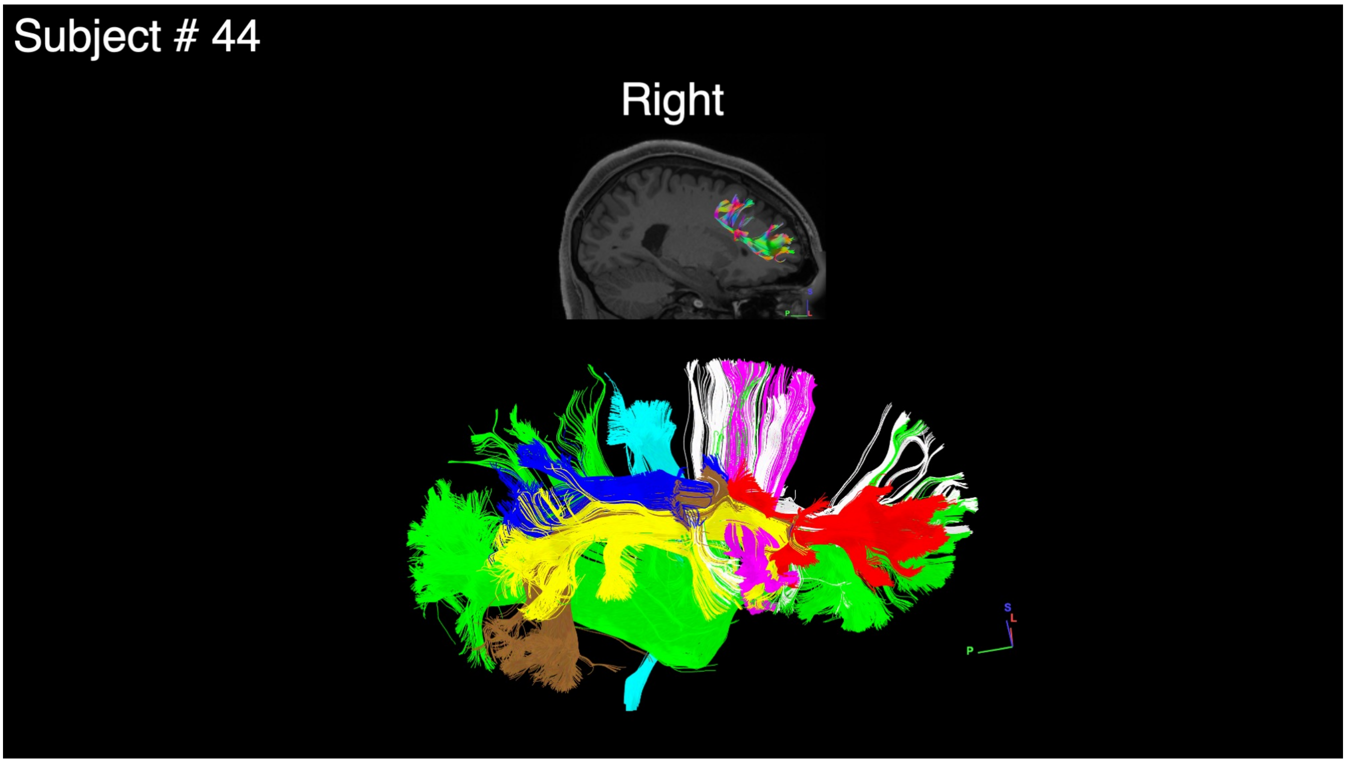
Subject # 44
Right

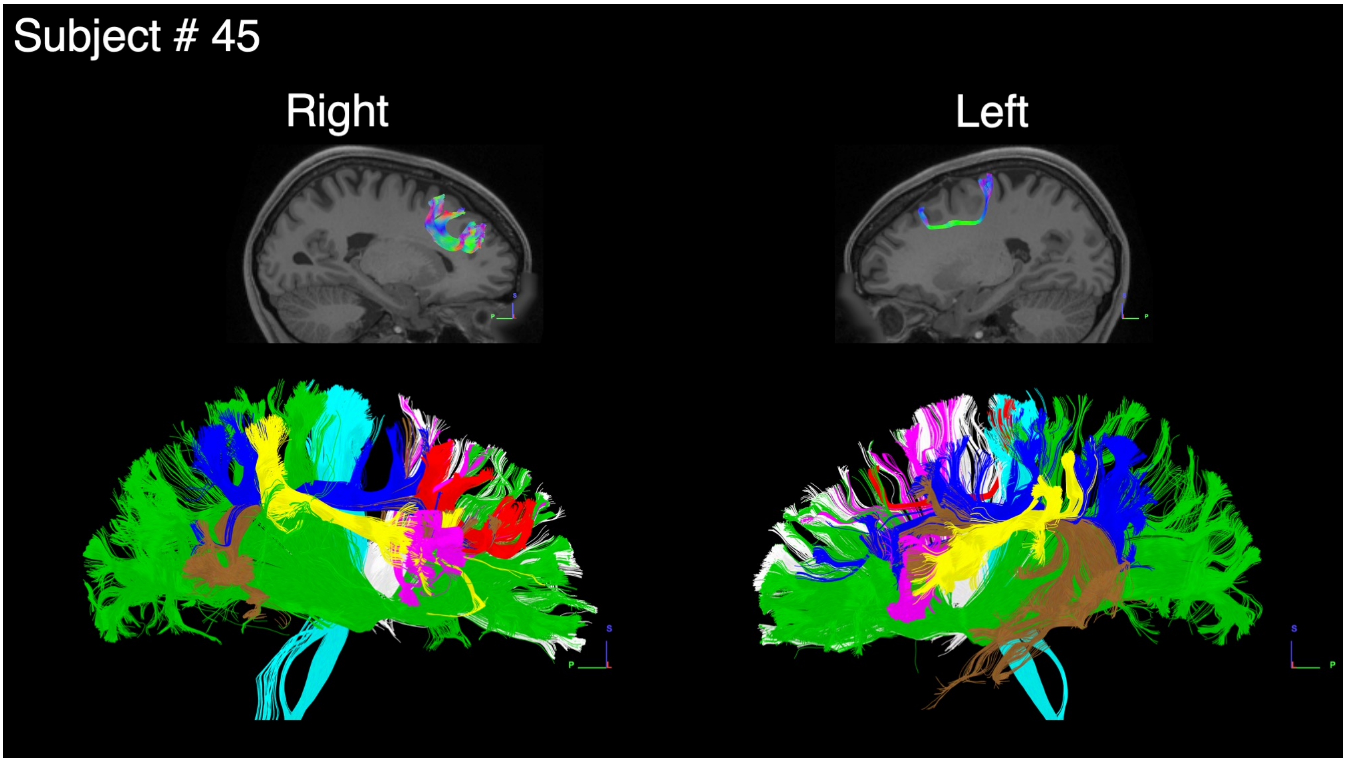
Subject # 45
Right
Left

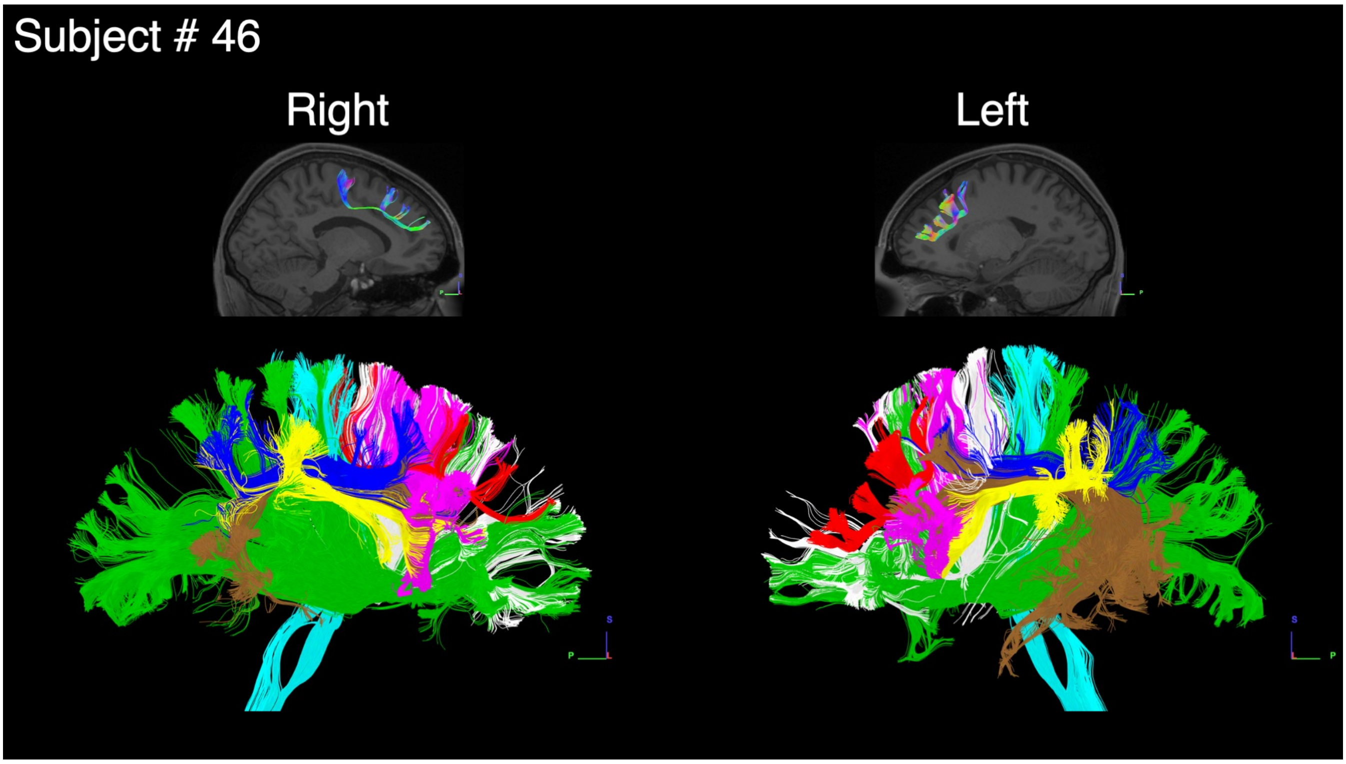
Subject # 46
Right
Left

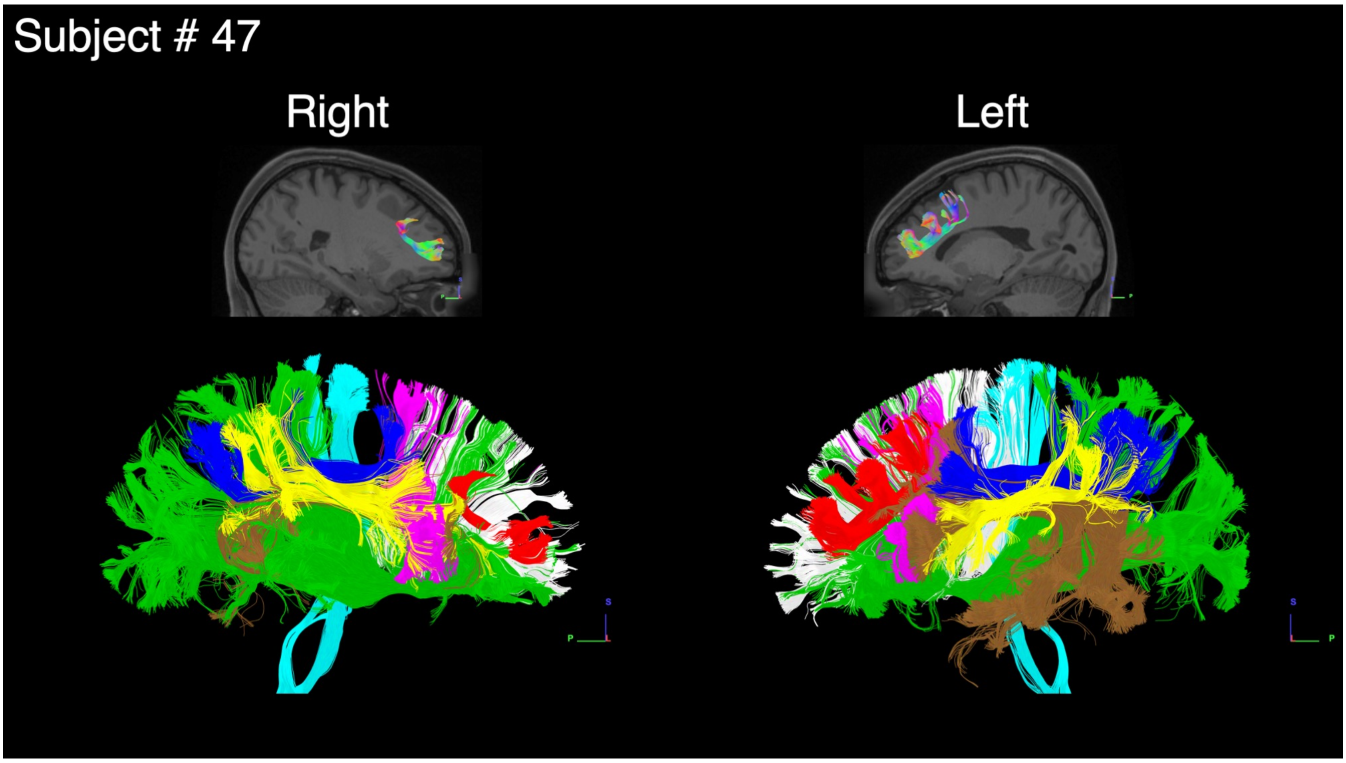
Subject # 47
Right
Left

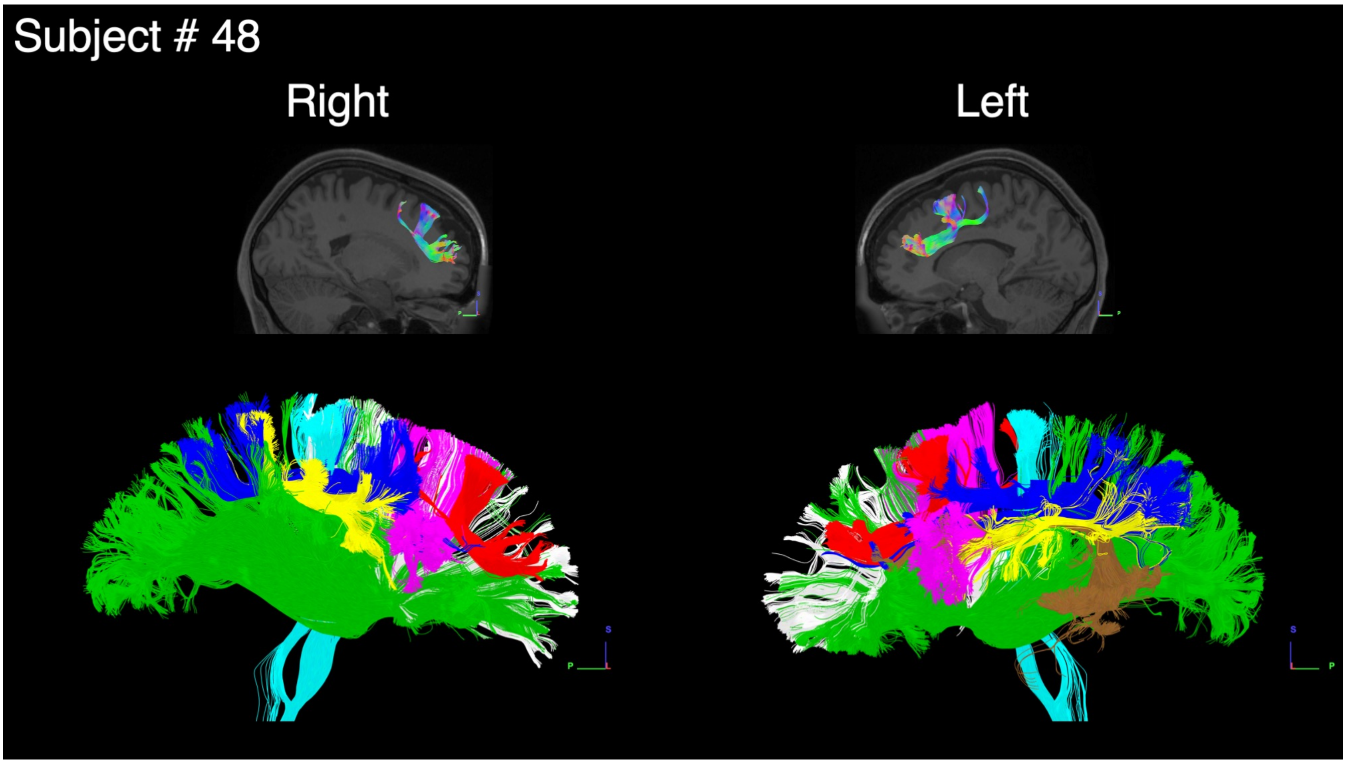
Subject # 48
Right
Left

Supplementary Fig. S2: SFLT and SLF spatial relation

GQI tractography of four subjects showing the spatial relation between the SFLT (red), SLF-II (blue) and SLF-III (green). SFLT demonstrated as an independent frontal intralobar tract and not a continuation of the SLF fibre system. The posterior terminations of SFLT and the anterior terminations of both SLFs are shown under their corresponding hemisphere. cMFG (brown) and PCG (purple).

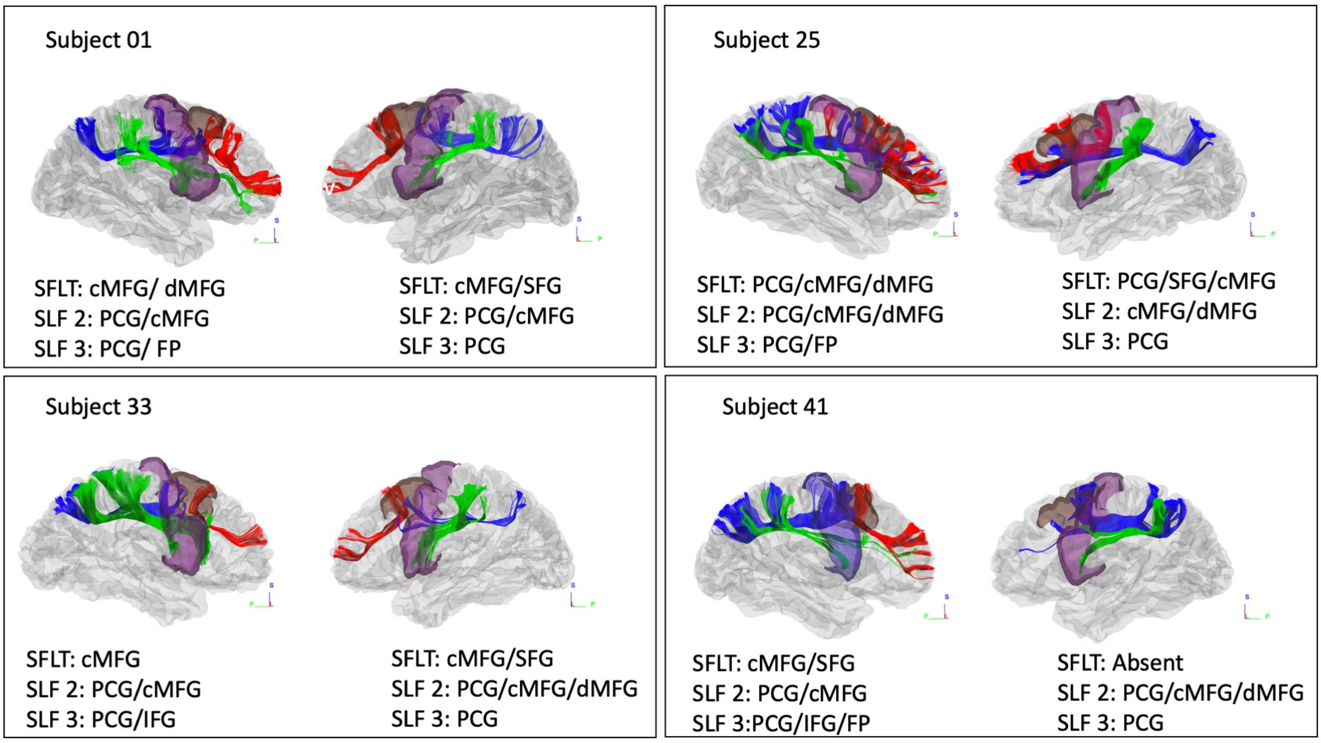

Supplementary Fig. S3: Laterality index (LI).

The laterality index distribution of the SFLT's normalised ratio (a), and fraction anisotropy (b).

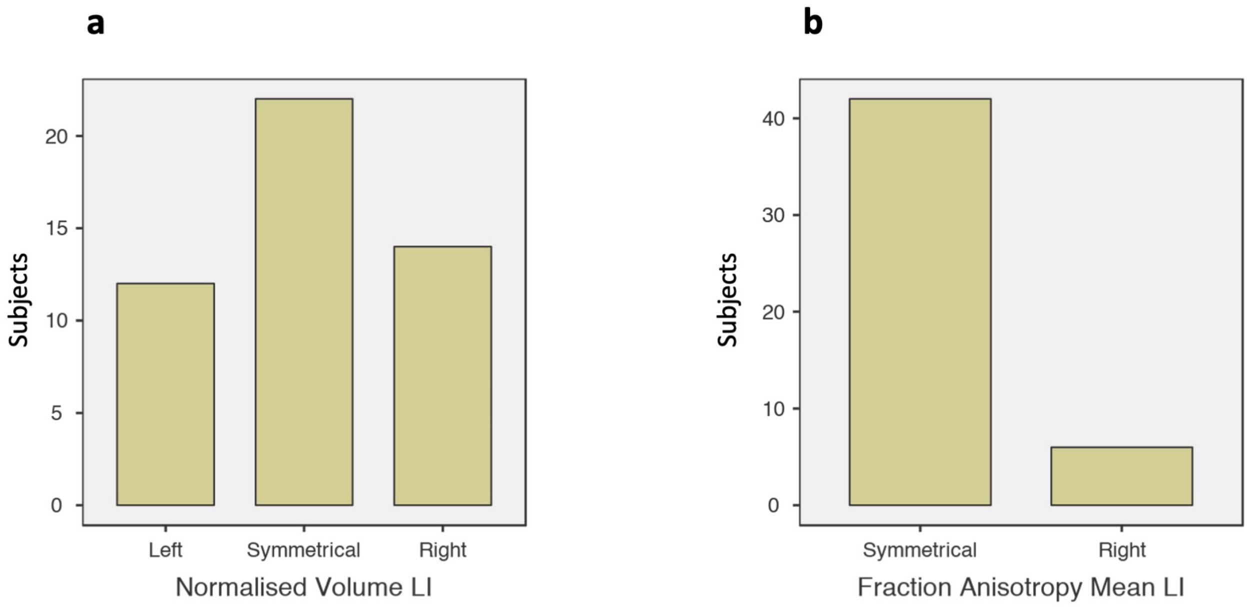

Supplementary Fig. S4: Types of SFLT based on lateralisation of its subcomponents.

The SFLT's subcomponents were grouped based on their caudal terminations, which were either a "motor segment" arising from the PCG (red) or a "premotor segment" arising from the MFG (green) and/or SFG (blue). The percentages represent the occurrence ratio within the SFLT's type in the current study sample. (+) Present, (-) Absent, BA Brodmann area.

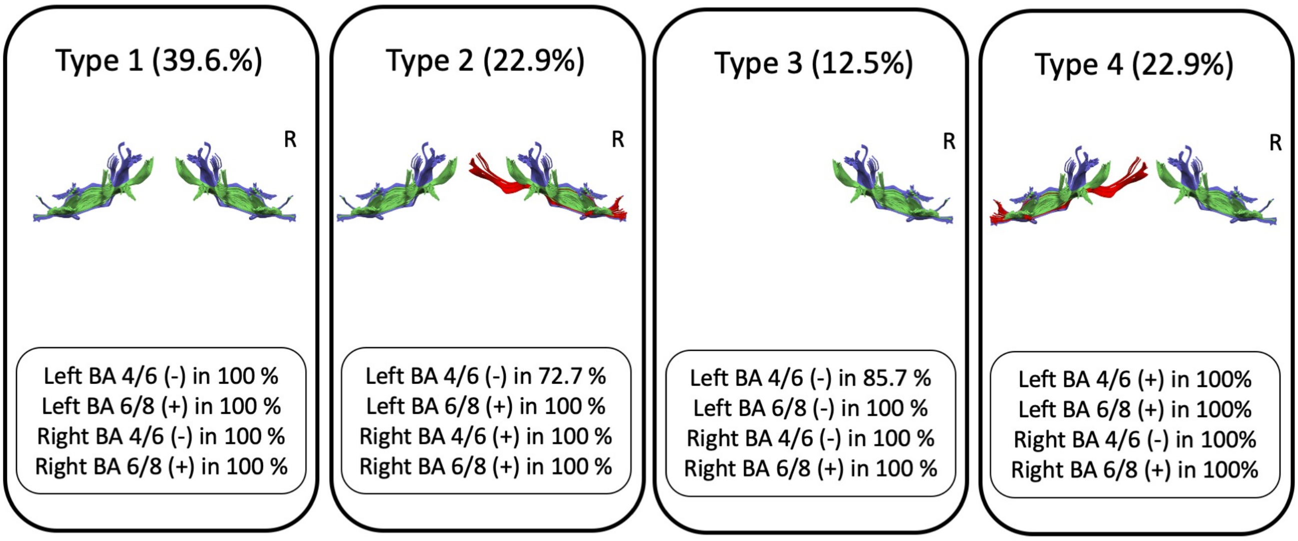

Supplement: Supplementary file 1 — Supplementary Figures. [file 41598_2020_73001_MOESM1_ESM.pdf]
