# Supplementary material for: The superior frontal longitudinal tract: a connection between the dorsal premotor and the dorsolateral prefrontal cortices
Source: Sci Rep. 2020 Sep 28;10:15855. doi: 10.1038/s41598-020-73001-7 (PMC7522085; doi:10.1038/s41598-020-73001-7)
Supplement: Supplementary file 2 — Supplementary Methods. [file 41598_2020_73001_MOESM2_ESM.pdf]

The superior frontal longitudinal tract: a connection between the dorsal premotor and the dorsolateral prefrontal cortices

Supplementary methods

Mudathir Bakhit[1*], Masazumi Fujii[1], Ryo Hiruta[1], Masayuki Yamada[1], Kenichiro Iwami[2] Taku Sato[1], Kiyoshi Saito[1]

[1]Department of Neurosurgery, Fukushima Medical University, 1 Hikarigaoka, Fukushima, 960-1295, Japan.

[2]Department of Neurosurgery, Aichi Medical University, 1-1 Yazakokarimata, Nagakute, Aichi, 480-1195, Japan.

***Corresponding author:**

Mudathir Bakhit

Department of Neurosurgery

Fukushima Medical University

Fukushima city- Japan

Tel: +81-24-547-1268

Fax: +81-24-548-1803

Email: m-bakhit@fmu.ac.jp

Here, we will conduct a virtual dissection of the left SFLT in subject # 1. When setting the configuration for the tractography process (see the methods section in the main text), in the following example, we will not include a streamline minimum length setting. Thus, all short streamlines will be reconstructed. The purpose is to demonstrate how to virtually dissect the SFLT in the presence of a mass of other short streamlines. However, in typical situations, the minimum length setting does save the examiner a significant amount of time.

The white matter and the merged rMFG/FP masks are selected as the 'seed' and 'end' regions, respectively (Fig. S1 a); then, the process of tractography can be initiated. After the reconstruction finishes, the created streamlines will represent the projection, association, and commissural streamlines terminating in the rMFG/FP area of the same hemisphere (Fig. S1 b-d).

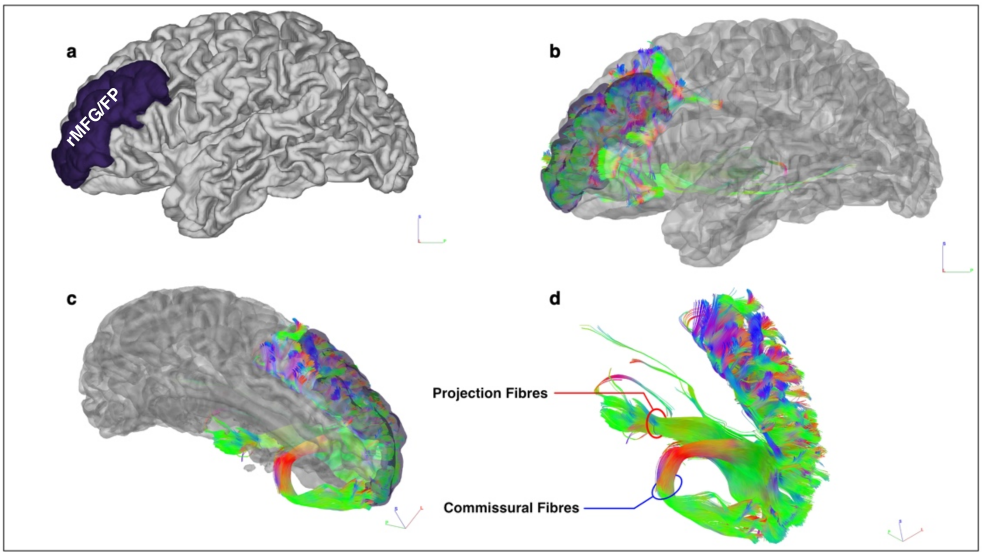

Fig. S1. The process of tractography. The white matter mask is selected as the seed region and the merged rMFG/FP mask as the end region (a). By reducing the opacity of the masks, the underneath streamlines can be seen from the lateral (b) and medial (c) aspects. The masks are removed, and the whole rendered streamlines are visualised (d).

In Figure S2, after removing the white matter masks, we can see the reconstructed streamlines beneath the rMFG/FP mask from the lateral surface. Here, it is mainly a mass of U-shape fibres over the whole superficial surface. Next, the commissural and projection fibres are discarded (Fig. S2 b).

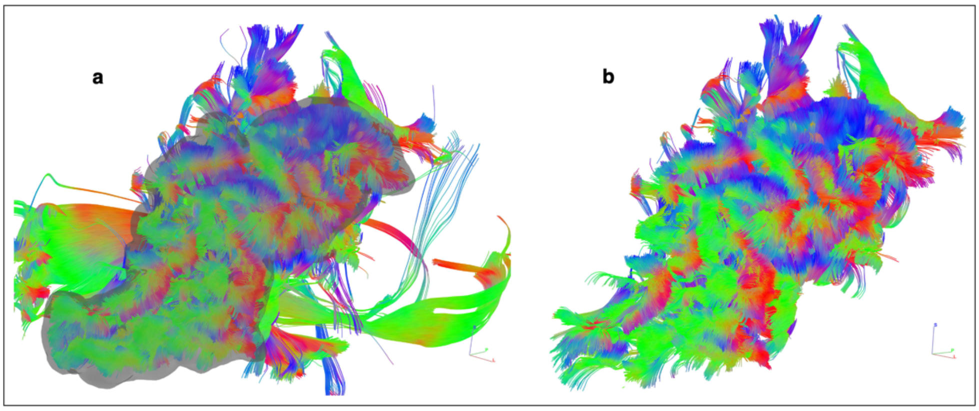

Fig. S2. The lateral view of the rMFG/FP region. (a) The rendered streamlines beneath the rMFG/FP mask, including the projection and commissural fibres. (b) The rMFG/FP mask, the projection, and the commissural fibres are discarded, and the remaining streamlines are the association fibres terminating in the rMFG/FP area.

Then, we shift the view to the ventro-medial aspect to inspect the deep layer of the association fibres, searching for rostro-caudal longitudinal streamlines and eliminating the short U-shape fibres (Fig. S3 left). In the example attached, we can see a group of streamlines that possess a longitudinal pattern with a rostro-caudal stream (below the dotted lines a-c). These streamlines view is obliterated from the lateral surface because they are covered by the superficial U-shape streamlines (Fig. S2). Using the DSI studio's selecting tool, in this example, the streamlines below the dotted line (c) were selected. The result was a group of streamlines representing different bundles (Fig. S3 right).

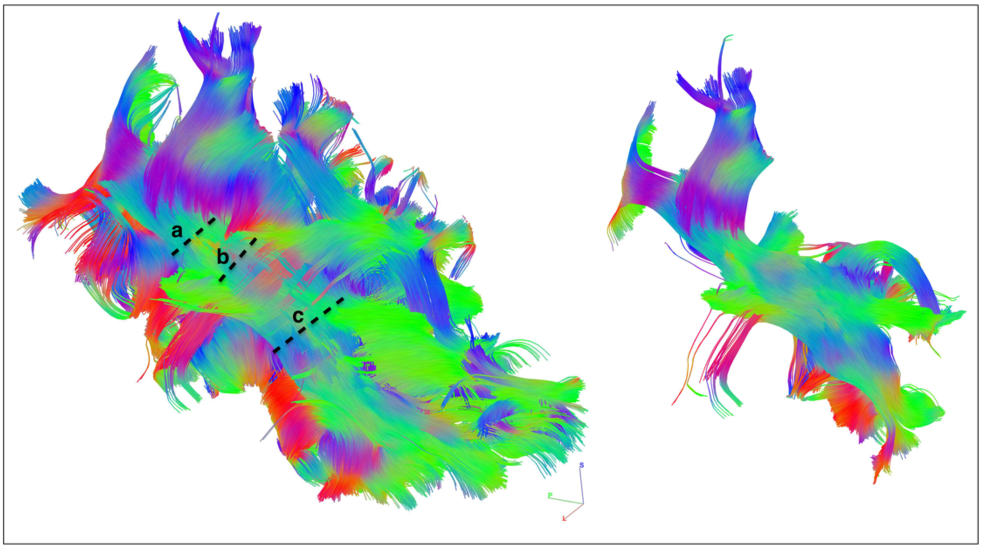

Fig. S3. The ventro-medial aspect of the association tracts. (*left*) The association streamlines terminating in the rMFG/FP area are of various lengths and shapes. In the deepest layers, a group of streamlines with a rostro-caudal longitudinal stream can be seen (perpendicular to the dotted lines a-c). (*right*) By selecting the group of streamlines below the dotted line (c), a rostro-caudal longitudinal bundle with a mixture of various lengths is revealed.

Then, the selected streamlines are viewed from the lateral surface of the hemisphere with the cortical masks (PCG, SFG, cMFG) (Figure S4). This group of streamlines have no terminations in the PCG. Moreover, the most posterior termination points are in the anterior part of cMFG, and the middle third of the SFG. The streamlines still include a mixture of short U-shape and longitudinal streamlines. By selecting the streamlines that terminate in the cMFG, and SFG (below the red dotted line in Figure S4 left), the result will be an isolated SFLT.

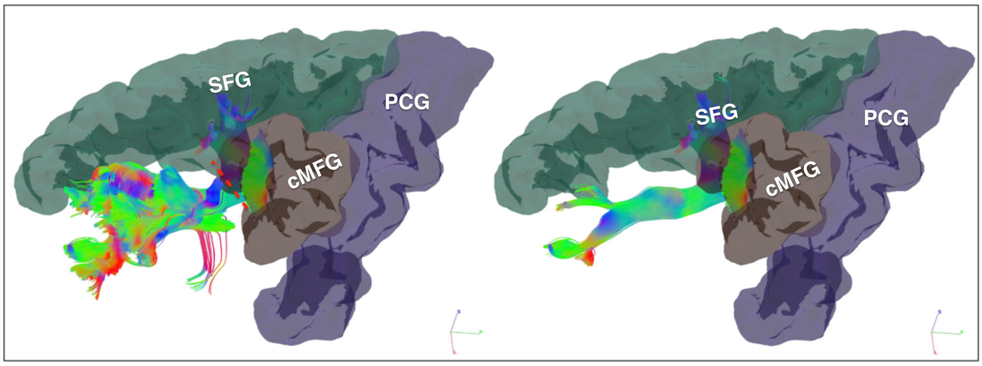

Fig. S4. The selected streamlines with the cortical masks, lateral view. (left) The streamlines selected in Figure S3 are displayed from the lateral surface with the cortical masks of the PCG, SFG, and cMFG. Selection of streamlines terminating in the SFG, and cMFG (under the red dotted line) will reveal the SFLT's streamlines (right).

After removing all the cortical masks, the SFLT's whole structure can be seen from the lateral and the ventro-medial aspects (Fig. S5).

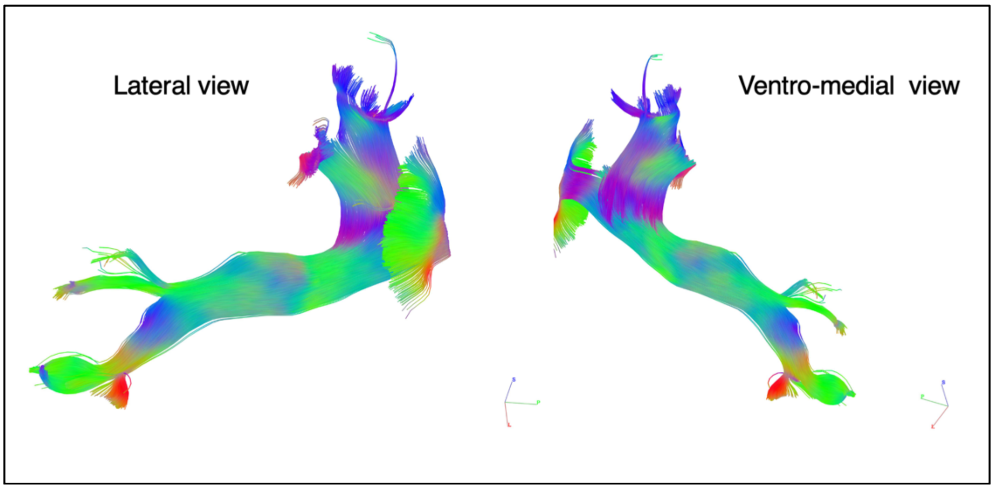

Fig. S5. The rendered SFLT's streamlines. The SFLT from the lateral (left), and the ventro-medial (right) surface of the left hemisphere.

The previously eliminated association fibres can be reloaded for further inspection and search for other rostro-caudal longitudinal streamlines(Fig. S6). In case other SFLT's streamlines are found they can be merged with the previously rendered ones.

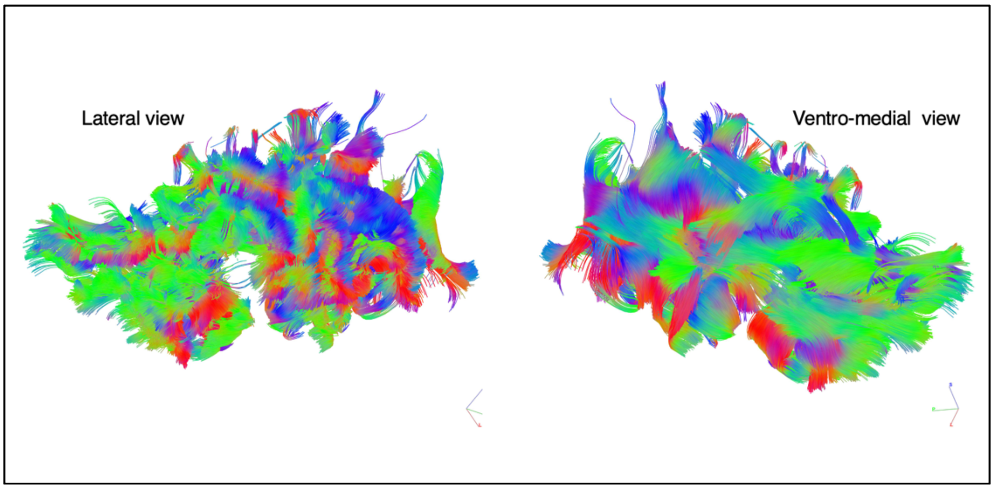

Fig. S6. The remaining streamlines. The previously eliminated association streamlines in previous steps are reloaded and inspected from the lateral (left) and ventro-medial (right) aspects.

By inspection, a rostro-caudal stream-like can be observed from the ventro-medial view (Figure S6 right). In repetition to the previous steps, the tracts below the dotted lines in Figure S7 were selected. In contrast to SFLT, the result was two separate short U-shape bundles. One bundle has its posterior termination in the cMFG, the other located in the substance of the rMFG. No other long bundles to add to the SFLT were found. The remaining short streamlines were discarded.

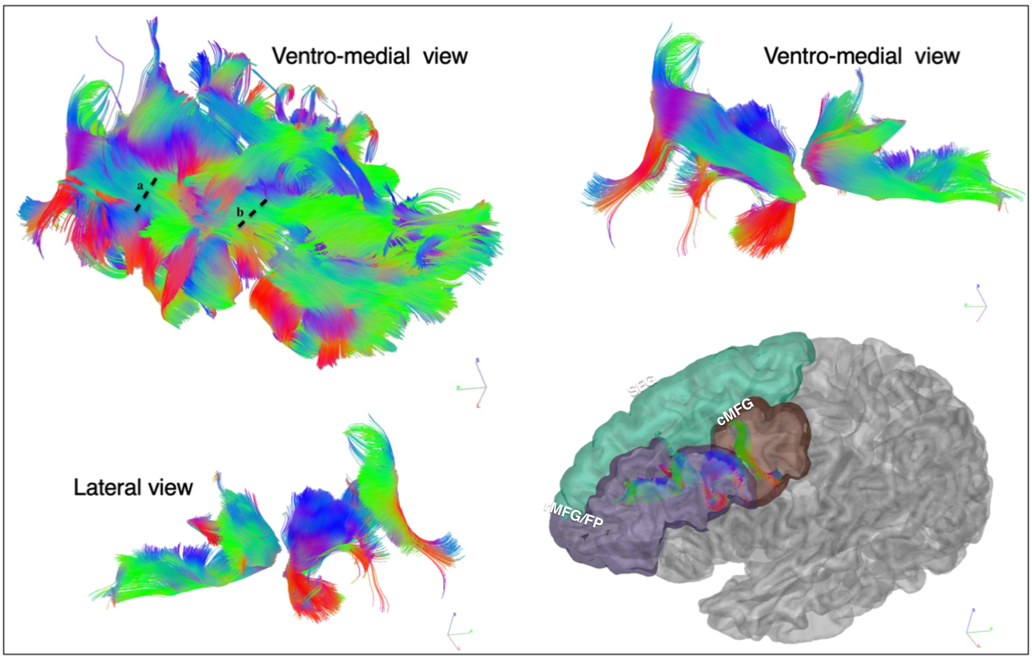

Fig. S7. The short streamlines. After selecting the streamlines below the dotted lines (a and b), the result was two short U-shape bundles.

DSI Studio allows the measurement of the mean length of the reconstructed tracts. The groups of streamlines selected at the dotted line (a) and (b) in Figure S7, had means length of 48 mm (green), and 42 mm (blue), respectively (Fig. S8). On the other hand, the SFLT had a mean length of 83 mm. The SFLT's mean length was almost double the short segments, although it lacks a PCG subcomponent. We assume these short streamlines to be the sFLS described by Catani et al.

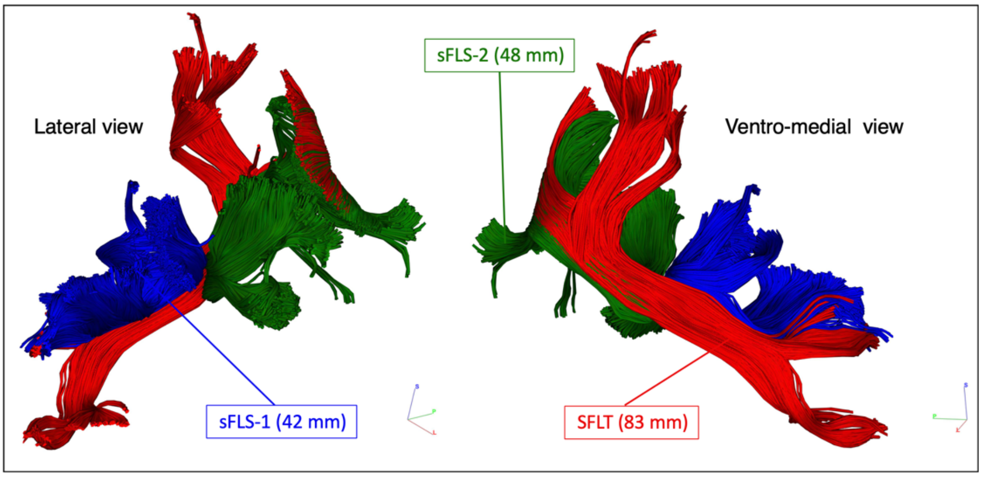

Fig. S8. The SFLT and sFLS. The SFLT is displayed in association with the sFLS from the lateral (left), and ventro-medial (right) aspects. The mean lengths of the SFLT and both sFLS bundles were 83 mm, 48 mm, and 42 mm, respectively.

Moreover, the MFG and SFG subcomponents can be inspected separately (Fig. S9 a, b) or combined (Fig. S9 c). Both subcomponents appear as 'tract-like' structures. They share the same body and the anterior terminations but have different posterior terminations (Fig. S9 c). By visual inspection, we can realise that both subcomponents share a similar BA at the posterior terminations (BA 6-rostral/8).

This step concludes the virtual dissection process and the rendered SFLT's volume and means FA can be obtained for further analysis.

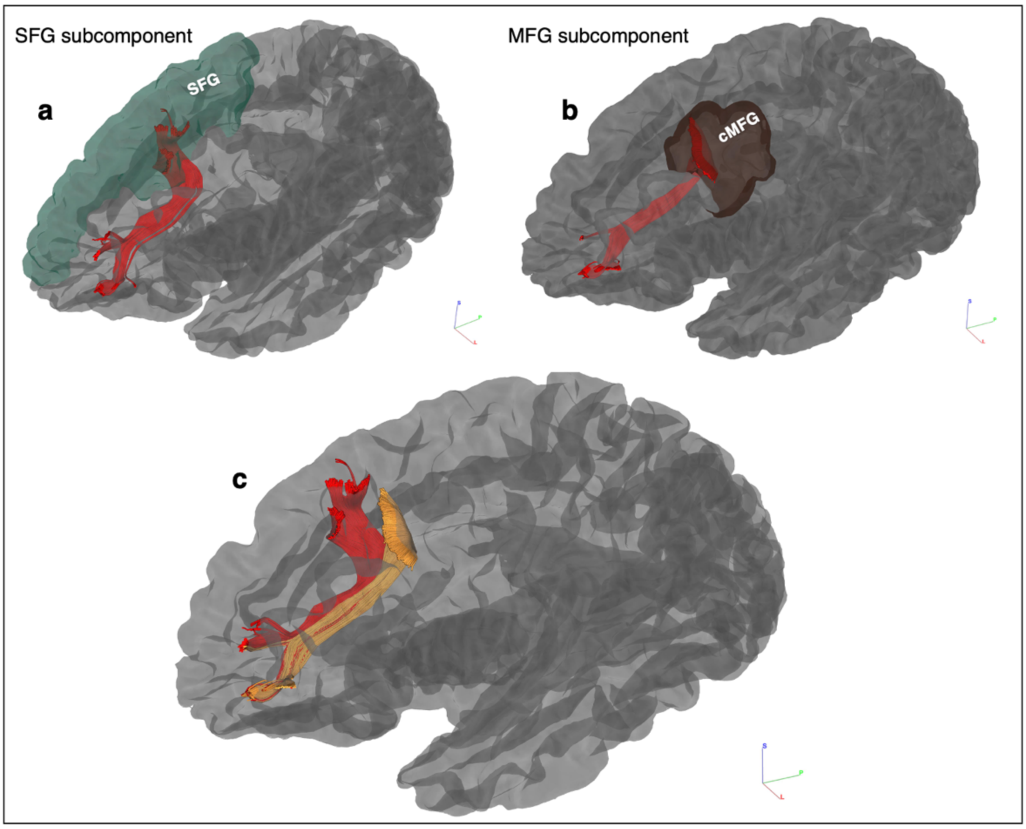

Fig. S9. The SFLT's subcomponents. In subject #1, the SFLT was made of two subcomponents, the SFG (a), and cMFG (b). Both subcomponents shared the same body and anterior terminations but had different posterior terminations (c).

In the current example, we included an additional task. For comparison, the SFLT was mapped against the ipsilateral frontoparietal association bundles, the SLF-II and SLF-III (Fig. S10). The purpose of this task is to evaluate if the SFLT's subcomponents can be viewed as 'tract-like' structures. Although the current sample lacks a PCG segment, both subcomponents' morphology and lengths were comparable to the frontoparietal bundles. The means length of the SLF-II and SLF-III were 89 mm, and 63 mm, respectively. On the other hand, the SFG and MFG subcomponents were 82 mm, and 84 mm, respectively. Both subcomponents were longer than the SLF-III and slightly shorter than the SLF-II.

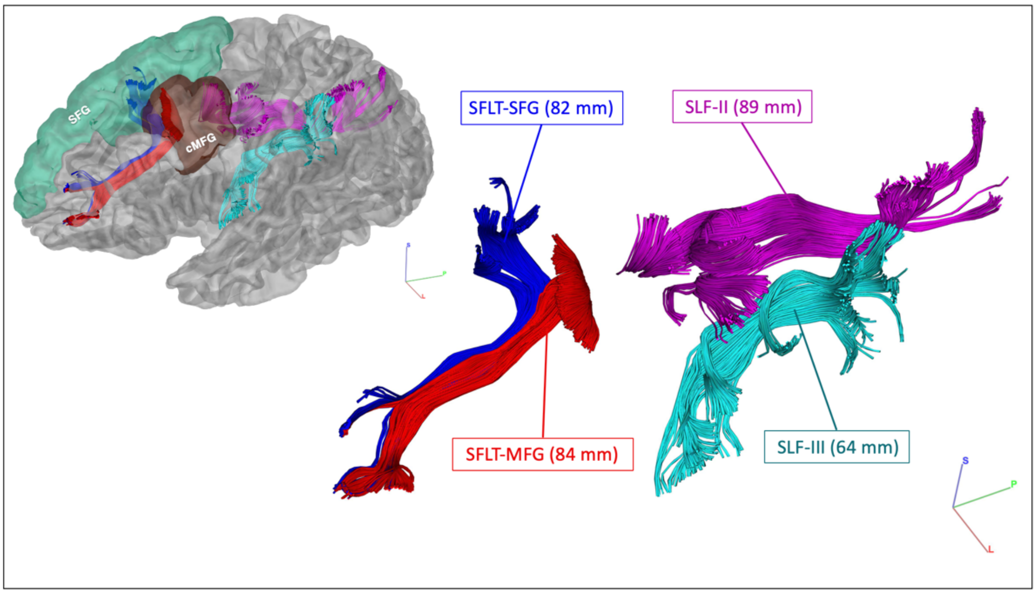

Fig. S10. The SFLT's subcomponents and the frontoparietal bundles. The SFLT's SFG (blue), and MFG (red) subcomponents exhibited 'tract-like' structures and their lengths were comparable to the SLF-II (purple) and longer than the SLF-III (cyan).
