# Supplementary material for: The superior frontal longitudinal tract: a connection between the dorsal premotor and the dorsolateral prefrontal cortices
Source: Sci Rep. 2020 Sep 28;10:15855. doi: 10.1038/s41598-020-73001-7 (PMC7522085; doi:10.1038/s41598-020-73001-7)
Supplement: Supplementary file 3 — Supplementary Tables. [file 41598_2020_73001_MOESM3_ESM.pdf]

The superior frontal longitudinal tract: a connection between the dorsal premotor and the dorsolateral prefrontal cortices

Supplementary tables

Mudathir Bakhit[1*], Masazumi Fujii[1], Ryo Hiruta[1], Masayuki Yamada[1], Kenichiro Iwami[2] Taku Sato[1], Kiyoshi Saito[1]

[1]Department of Neurosurgery, Fukushima Medical University, 1 Hikarigaoka, Fukushima, 960-1295, Japan.

[2]Department of Neurosurgery, Aichi Medical University, 1-1 Yazakokarimata, Nagakute, Aichi, 480-1195, Japan.

***Corresponding author:**

Mudathir Bakhit

Department of Neurosurgery

Fukushima Medical University

Fukushima city- Japan

Tel: +81-24-547-1268

Fax: +81-24-548-1803

Email: m-bakhit@fmu.ac.jp

Supplementary Table S1. SFLT and its subcomponents: a descriptive analysis.

	Subjects (%)	Mean ± SD
Left SFLT	42 (88%)	
Normalised volume %		0.63 ± 0.42
FA mean		0.55 ± 0.22
Right SFLT	48 (100%)	
Normalised volume %		0.66 ± 0.39
FA mean		0.62 ± 0.07
Left MFG subcomponent	38 (79%)	
Normalised volume %		0.38 ± 0.31
FA mean		0.50 ± 0.27
Right MFG subcomponent	45 (94%)	
Normalised volume %		0.50 ± 0.39
FA mean		0.58 ± 0.17
Left PCG subcomponent	15 (31%)	
Normalised volume %		0.07 ± 0.14
FA mean		0.21 ± 0.31
Right PCG subcomponent	11 (23%)	
Normalised volume %		0.05 ± 0.11
FA mean		0.15 ± 0.27
Left SFG subcomponent	29 (60%)	
Normalised volume %		0.22 ± 0.25
FA mean		0.37 ± 0.31
Right SFG subcomponent	21 (44%)	
Normalised volume %		0.15 ± 0.19
FA mean		0.25 ± 0.31

Supplementary Table S2. LI pattern distribution and correlation with gender and handedness.

Tract	LI Pattern	Subjects	Gender			Handedness		
		Total (%)	Female	Male	*p*	Right	Left	*p*
SFLT								
LI (Vol)	Left	12 (25 %)	6	6	0.53*	7	5	0.40*
	Symmetrical	22 (46 %)	12	10		8	14	
	Right	14 (29 %)	5	9		5	9	
LI (FA)	Left	-	-	-	0.67†	-	-	1.0†
	Symmetrical	42 (88 %)	21	21		18	24	
	Right	6 (13 %)	2	4		2	4	
MFG subcomponent								
LI (Vol)	Left	10 (21 %)	5	5	0.99*	5	5	0.58*
	Symmetrical	17 (35 %)	8	9		8	9	
	Right	21 (44 %)	10	11		7	14	
LI (FA)	Left	3 (6 %)	1	2	1.0†	1	2	1.0†
	Symmetrical	35 (73 %)	17	18		15	20	
	Right	10 (21 %)	5	5		4	6	
PCG subcomponent								
LI (Vol)	Left	13 (27 %)	8	5	0.32*	6	7	0.92*
	Symmetrical	25 (52 %)	12	13		10	15	
	Right	10 (21 %)	3	7		4	6	
LI (FA)	Left	12 (25 %)	7	5	0.73†	6	6	0.66†
	Symmetrical	28 (58 %)	13	15		10	18	
	Right	8 (17 %)	3	5		4	4	
SFG subcomponent								
LI (Vol)	Left	19 (40 %)	8	11	0.79†	8	11	0.80†
	Symmetrical	22 (46 %)	11	11		10	12	
	Right	7 (15 %)	4	3		2	5	
LI (FA)	Left	15 (31 %)	7	8	1.0†	5	10	0.64†
	Symmetrical	27 (56 %)	13	14		13	14	
	Right	6 (13 %)	3	3		2	4	

*Chi-squared test, †Fisher's exact probability test

LI (Vol): Normalised volume LI, LI (FA): FA mean LI.

Supplementary Table S3. SFLT BA subcomponent frequency.

	Current study	Komaitis et al. (2019)
Left BA 4/6-caudal	15 (31%) subjects	6 (86%) left hemispheres
Left BA 6-rostral/8	41 (85%) subjects	7 (100%) left hemispheres
Right BA 4/6-caudal	11 (23%) subjects	4 (50%) right hemispheres
Right BA 6-rostral/8	48 (100%) subjects	8 (100%) right hemispheres

Supplementary Table S4. SFLT type distribution and correlation with gender and handedness groups.

SFLT Type	Count	Gender			Handedness		
	Total (%)	Female	Male	*p*	Right	Left	*p*
Type 1	19 (39.6%)	10	9	0.86†	8	11	1.0†
Type 2	11 (22.9%)	4	7		4	7	
Type 3	7 (12.5%)	3	4		3	4	
Type 4	11 (22.9%)	6	5		5	6	

†Fisher's exact probability test

Description of the SFLT's types is available in Supplementary Fig. S4.
